# Structural Basis for Native Agonist and Synthetic Inhibitor Recognition by the *Pseudomonas aeruginosa* Quorum Sensing Regulator PqsR (MvfR)

**DOI:** 10.1371/journal.ppat.1003508

**Published:** 2013-07-25

**Authors:** Aravindan Ilangovan, Matthew Fletcher, Giordano Rampioni, Christian Pustelny, Kendra Rumbaugh, Stephan Heeb, Miguel Cámara, Alex Truman, Siri Ram Chhabra, Jonas Emsley, Paul Williams

**Affiliations:** 1 Centre for Biomolecular Sciences, University of Nottingham, University Park, Nottingham, United Kingdom; 2 School of Molecular Medical Sciences, University of Nottingham, University Park, Nottingham, United Kingdom; 3 School of Pharmacy, University of Nottingham, University Park, Nottingham, United Kingdom; 4 Department of Surgery, University of Texas, Lubbock, Texas, United States of America; Massachusetts General Hospital, Harvard Medical School, United States of America

## Abstract

Bacterial populations co-ordinate gene expression collectively through quorum sensing (QS), a cell-to-cell communication mechanism employing diffusible signal molecules. The LysR-type transcriptional regulator (LTTR) protein PqsR (MvfR) is a key component of alkyl-quinolone (AQ)-dependent QS in *Pseudomonas aeruginosa*. PqsR is activated by 2-alkyl-4-quinolones including the *Pseudomonas* quinolone signal (PQS; 2-heptyl-3-hydroxy-4(1*H*)-quinolone), its precursor 2-heptyl-4-hydroxyquinoline (HHQ) and their C9 congeners, 2-nonyl-3-hydroxy-4(1*H*)-quinolone (C9-PQS) and 2-nonyl-4-hydroxyquinoline (NHQ). These drive the autoinduction of AQ biosynthesis and the up-regulation of key virulence determinants as a function of bacterial population density. Consequently, PqsR constitutes a potential target for novel antibacterial agents which attenuate infection through the blockade of virulence. Here we present the crystal structures of the PqsR co-inducer binding domain (CBD) and a complex with the native agonist NHQ. We show that the structure of the PqsR CBD has an unusually large ligand-binding pocket in which a native AQ agonist is stabilized entirely by hydrophobic interactions. Through a ligand-based design strategy we synthesized and evaluated a series of 50 AQ and novel quinazolinone (QZN) analogues and measured the impact on AQ biosynthesis, virulence gene expression and biofilm development. The simple exchange of two isosteres (OH for NH_2_) switches a QZN agonist to an antagonist with a concomitant impact on the induction of bacterial virulence factor production. We also determined the complex crystal structure of a QZN antagonist bound to PqsR revealing a similar orientation in the ligand binding pocket to the native agonist NHQ. This structure represents the first description of an LTTR-antagonist complex. Overall these studies present novel insights into LTTR ligand binding and ligand-based drug design and provide a chemical scaffold for further anti-*P. aeruginosa* virulence drug development by targeting the AQ receptor PqsR.

## Author Summary

Populations of bacterial cells collectively co-ordinate their activities through cell-to-cell communication via the production and sensing of signal molecules. This is called quorum sensing (QS) and in many bacteria, QS controls the expression of virulence genes, the products of which damage host tissues. Consequently, QS systems are potential targets for antimicrobial agents which do not kill bacteria but instead block their ability to cause disease. *Pseudomonas aeruginosa* causes a wide range of human infections and produces an armoury of virulence factors. Since many of these are controlled by alkylquinolone (AQ)-dependent QS, we determined the crystal structure of the AQ receptor (PqsR) in order to visualize the shape of the AQ-binding site and better design PqsR inhibitors which compete for the AQ binding site and so block QS. This work in conjunction with the chemical synthesis of AQ analogues resulted in the discovery of potent quinazolinone inhibitors of PqsR. These blocked AQ and virulence factor production in *P. aeruginosa* as well as biofilm development. Our studies present novel insights into the structure of PqsR and create further opportunities for target-based antibacterial drug development.

## Introduction

Bacterial cells communicate with each other through quorum sensing (QS), a mechanism for co-ordinating gene expression at the population level via the release and detection of self-generated signalling molecules [1]. Once a critical threshold concentration of QS signal has been attained, a change in collective behavior ensues through the activation of a sensor or regulator protein. In general, QS facilitates the coordination of population behavior to enhance access to nutrients, provide collective defense against other competitor organisms or to encourage community escape where population survival is at risk [1]. QS signal molecules are chemically diverse and include both small peptides and organic molecules such as the *N*-acylhomoserine lactones (AHLs) and 2-alkyl-4(1*H*)-quinolones (AQs). In addition, many bacteria possess several interacting QS modules organized into regulatory hierarchies employing multiple signal molecules from the same or different chemical classes. Such QS hierarchies regulate motility and biofilm development, secondary metabolite production, bioluminescence and virulence [1]. With respect to the latter, the global emergence of multi-antibiotic resistant bacteria and the paucity of new clinically effective antibiotics have renewed interest in the development of agents which control infection through the attenuation of bacterial virulence rather than inhibition of growth [2], [3]. In this context, the QS-dependent regulation of virulence offers an attractive suite of potential targets which include the QS signal synthase, the response regulator and the QS signal molecule itself [4], [3].

**Figure 1:**
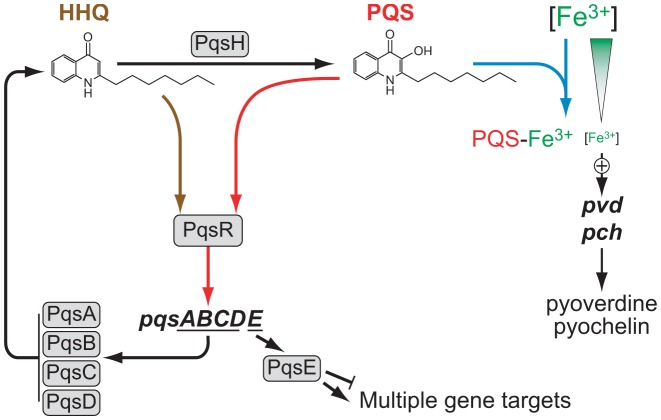
Diagrammatic representation of the AQ-dependent QS network in *Pseudomonas aeruginosa*. The PqsABCD proteins synthesize HHQ, which is converted to PQS by PqsH. Autoinduction occurs when either HHQ or PQS binds to PqsR and amplifies expression of the *pqsABCDE* operon. The terminal output of this regulatory network is PqsE, a putative metallohydrolase protein of unknown enzymatic function which positively regulates virulence genes, secondary metabolites and biofilm development when expressed in the absence of HHQ and PQS. The PqsE regulatory pathway also downregulates *pqsA* and AQ biosynthesis. The conversion of HHQ to PQS confers additional functionalities since PQS unlike HHQ induces microvesicle formation and is a potent iron chelator which induces expression of the pyoverdin and pyochelin high affinity iron transport systems. AQ-dependent QS is closely linked to the AHL-dependent *las* and *rhl* QS systems. The *las* system positively regulates the transcription of *pqsR*, *pqsABCDE* and *pqsH* while *rhl* exerts a negative effect on the AQ system, although it is itself positively regulated by AQs. Filled arrows and blunted lines represent positive and negative regulation, respectively.

While there have been extensive attempts to unravel the molecular basis for AHL-dependent QS and to develop inhibitors directed against LuxR-type transcriptional regulators, there is relatively little structural information on the recognition and mechanism of action or inhibition of AQ-type QS signals. AQs are produced by pathogens such as *Burkholderia pseudomallei* and *Pseudomonas aeruginosa* [5], [6]. *P. aeruginosa* thrives in diverse ecological niches and causes both acute and chronic infections in humans, animals, plants and insects. Multi-antibiotic resistant strains have emerged globally as a major cause of hospital-acquired infections for which current therapeutic options are very limited [7]. *P. aeruginosa* produces diverse exotoxin virulence determinants and secondary metabolites including cyanide, readily forms biofilms and is naturally resistant to many antimicrobial agents. Since many of these virulence genes are controlled by QS [8], *P. aeruginosa* has emerged as a paradigm pathogen since it employs a sophisticated multi-signal QS system incorporating both AHL/LuxR type and AQ-dependent gene regulatory systems [8] (**Figure 1**). With respect to the AQs, *P. aeruginosa* produces over 50 different congeners which were originally identified via their antimicrobial properties but are now known to possess QS, immune modulatory, cytochrome inhibitory, metal chelating, membrane vesicle-stimulating and oxidant activities (reviewed in [9]).

2-Heptyl-3-hydroxy-4(1*H*)-quinolone (the ‘Pseudomonas Quinolone Signal’, PQS) and its immediate precursor, 2-heptyl-4-hydroxyquinoline (HHQ) are frequently considered to be the primary AQs involved in QS although other active AQ analogues, notably the C9 congeners, 2-nonyl-3-hydroxy-4(1*H*)-quinolone (C9-PQS) and 2-nonyl-4-hydroxyquinoline (NHQ) are produced by *P. aeruginosa* in similar concentrations [10], [11]. The synthesis and action of PQS and HHQ and related congeners depends on the *pqsABCDE* operon, which is positively controlled by the transcriptional regulator PqsR (MvfR) [12], [13]. The first four gene products of this operon are required for AQ biosynthesis [9]. HHQ is released into the extracellular milieu where it is internalized via adjacent cells [14] and oxidized to PQS via the action of the mono-oxygenase PqsH [5], [13], [15]. The function of the *pqsE* gene product, a putative metallohydrolase, is not currently understood. Although it does not contribute to AQ biosynthesis, it is required for swarming motility biofilm development and virulence and is involved in the negative regulation of the *pqsABCDE* operon [15], [16]. Strains with mutations in *pqsR* and *pqsA* are severely attenuated in experimental animal infection models highlighting the important contribution made by AQ signalling to pathogenicity [16], [12]. Furthermore the presence of AQs in the sputum and broncho-alveolar lavage fluid of cystic fibrosis patients chronically infected with *P. aeruginosa* provides evidence of their importance in human infection [17], [18].

AQ synthesis and *pqsE* expression are subject to a positive feedback loop which involves the activation of PqsR by HHQ and PQS and their C9 congeners to drive the expression of the *pqsABCDE* operon [14], [19], [20], [21], [22]. In whole *P. aeruginosa* cell assays, HHQ and PQS exhibited EC_50_s in the low micromolar range for the PqsR-dependent activation of *pqsA* [23]. Activation of PqsR depends on the AQ alkyl chain length [23], PQS congeners with C1, C3, C5 or C11 alkyl chains exhibit only weak activities compared with the C7 compounds. However, the C9 congeners, 3-hydroxy-2-nonyl-4(*1H*)-quinolone (C9-PQS) and 2-nonyl(*1H*)-4-hydroxyquinoline (NHQ) are highly active [23]. Although both HHQ and PQS can activate PqsR, the PqsH-mediated introduction of a hydroxyl group at the 3 position of the quinolone ring confers additional physicochemical functionality to PQS over HHQ including iron chelation [14] and microvesicle formation [24].

Attempts to attenuate AQ signaling and the virulence of *P. aeruginosa* without perturbing bacterial growth have so far mainly focused on enzymes which inactivate PQS [25] and methylated or halogenated derivatives of the AQ precursor anthranilate such as 2-amino-4-chorobenzoic acid (4-CABA) which inhibits AQ biosynthesis probably at the level of PqsA by competing with anthranilate for the enzyme active site [26], [27]. This approach has recently shown promising results in limiting the systemic proliferation of *P. aeruginosa* infection in mice although the concentrations required to inhibit AQ production were high (millimolar range; [27]) and so unlikely to be clinically useful. More recently a number of PqsD inhibitors have been identified [28].

**Figure 2:**
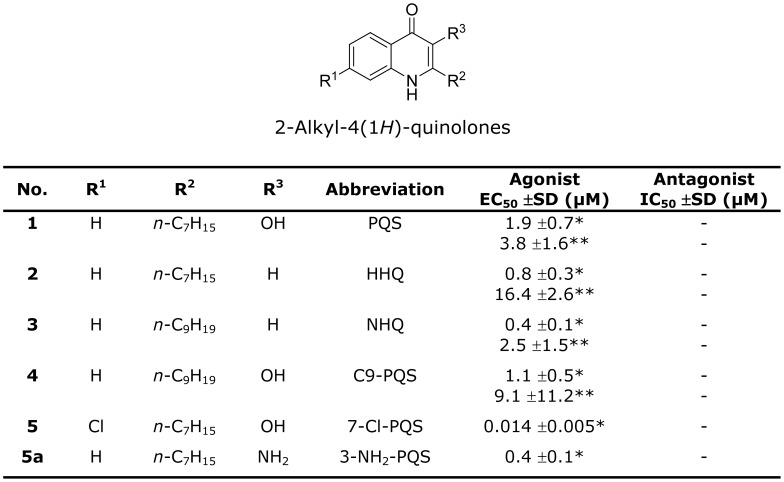
Activation and inhibition of PqsR in *P. aeruginosa* by the 2-alkyl-4(1*H*)-quinolones. *EC_50_ determined in a *P. aeruginosa* Δ*pqsA* CTX::*pqsA'-lux* strain; **EC_50_ determined in a *P. aeruginosa* Δ*pqsAH* CTX::*pqsA'*-lux strain; – no activity.

An alternative approach for blocking AQ biosynthesis and signalling and hence virulence would be to target the response regulator PqsR. Although the structures of the PqsR-activating ligands PQS and HHQ are known (**Figure 2**), there is no information on the PqsR ligand binding site. PqsR belongs to the LysR family of transcriptional regulators (LTTRs) which are widespread in bacteria [29] among which *P. aeruginosa* appears to have one of the largest repertoires [30]. LTTR proteins generally possess a highly conserved *N*-terminal helix-turn-helix (HTH) DNA-binding domain but a poorly conserved *C*-terminal ligand-binding domain usually termed the co-inducer binding domain (CBD) where the co-inducer is a low molecular weight ligand [29]. LTTRs function as either activators or repressors in feedback loops in which the co-inducer ligand is required for transcriptional control. LTTR proteins are generally thought to function as tetramers recognizing multiple binding sites within the promoter/operator region of the target gene(s) which include a regulatory binding site (RBS) incorporating the LTTR box (general consensus T-N_11_-A) and an activation binding site (ABS). Occupation of these sites results in DNA bending and contact with RNA polymerase to initiate transcription [29]. Structural studies of LTTRs have been mostly restricted to the C-terminal CBD because of the insolubility associated with the HTH domain [29]. LTTR CBDs typically have two α/β sub-domains (CBDI and CBDII) connected by an anti-parallel β-sheet known as the hinge region [31], [32]. Well characterised ligands for LTTRs include catechols and chlorinated aromatics which are co-inducers for CatM and BenM respectively binding into a pocket between the CBD subdomains [29].

With respect to PqsR, how this unusual LTTR [30] recognizes and responds to the larger hydrophobic ligands associated with AQ-dependent QS is not known. The structural basis for the recognition of AQs by PqsR has not been elucidated and there is consequently a lack of molecular detail to facilitate the development of PqsR inhibitors as novel therapeutics. Through a multidisciplinary effort we provide new insights into the structure of the PqsR co-inducer binding domain (PqsR^CBD^) in complex with both a native AQ agonist and a potent quinazolinone (QZN) antagonist. The QZN scaffold was evolved through ‘ligand based design’ and we show that in *P. aeruginosa* a very simple isosteric replacement is sufficient to switch a QZN from potent agonist to potent antagonist turning QS-dependent virulence gene expression on or off respectively. Although other LTTR agonist complex structures have been described this, to our knowledge, is the first description of an LTTR-antagonist complex crystal structure.

## Results

### Structure of the PqsR Co-inducer Binding Domain

**Figure 3:**
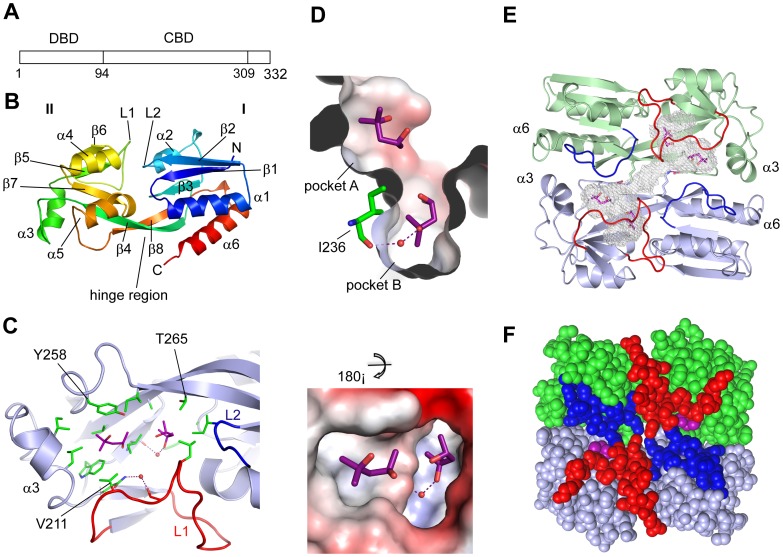
Crystal Structure of the PqsR^CBD^. (**A**) Diagrammatic representation of the linear PqsR protein showing the positions of the DNA-binding (DBD) and co-inducer-binding (CBD) domains; (**B**) Topology diagram of the PqsR^CBD^ monomer consisting of two sub-domains and a hinge region; (**C**) PqsR^CBD^ hydrophobic ligand-binding pocket with two bound MPD molecules shown as purple sticks; (**D**) Charged surface representation of the A and B pockets shown as two views related by a 180° rotation. Hydrogen bonds are shown as dotted lines and water molecules as red spheres; (**E**) The PqsR^CBD^ dimer shown as a topology diagram with bound MPD molecules shown as sticks. Loops forming a lid are coloured red and blue. The Lys266 and Glu259 residues are shown as sticks with the salt bridge shown as a purple dotted line. The pocket shape is shown as a white mesh. (**F**) The PqsR^CBD^ dimer shown as solid spheres with the same colour scheme.

To investigate the molecular basis of PqsR ligand recognition we first sought to determine the crystal structure. The full length PqsR receptor containing both the DNA-binding domain and CBD was insoluble when expressed in *Escherichia coli*. We thus focused on a construct spanning *C*-terminal residues 94-332 incorporating the CBD (**Figure 3A**). This was soluble and was utilised for initial crystallisations together with a truncated version which removed the 23 amino acid C-terminal tail. Crystals were obtained only in conditions where the precipitant, 2-methyl-2,4-pentanediol (MPD) was present and the structure was determined using SAD and SIR phases in spacegroup P6_5_22 to 2.5 Å resolution (**Table 1**). Crystallisation of the PqsR^CBD^ has been reported previously by two groups at 5 Å [33] and 3.25 Å resolution respectively [34]. The latter P6_5_22 crystal form has similar cell dimensions to the crystal form reported here although the crystallisation conditions differ and MPD is not present. The construct reported in Xu *et al.* [34] has different domain boundaries spanning residues 91–319 compared to residues 94–294 and 94–332 reported here.

**Table 1:** Crystallographic data collection and refinement statistics.

Sample	PqsR native MPD	PqsR – K_2_PtCl_4_	PqsR – Se-Met	PqsR – NHQ	PqsR – 3NH_2_- 7Cl-C_9_QZN
Space group Unit Cell (Å)	P6_5_22	P6_5_22	P6_5_22	P6_5_22	P6_5_22
*a*	119.7	118.95	119.33	120.43	118.4
*b*	119.7	118.95	119.33	120.43	118.4
*c*	115.8	115.63	115.81	115.43	115.4
Wavelength	0.9770	1.07112	0.97930	0.97630	0.97625
Unique reflections	17339	6586	6560	11482	11028
Resolution (Å)	2.5	3.48	3.5	2.9	2.95
I/Sigma*	22.4 (2.0)	20.1 (6.9)	20.5 (3.4)	17.6 (2.5)	16.0 (2.0)
Rmerge^a^	0.044 (0.740)	0.067 (0.219)	0.071 (0.238)	0.065 (0.830)	0.069 (0.780)
Completeness (%)	99.2 (99.9)	99.9 (100)	99.8 (100)	99.7 (100)	99.4 (100)
Multiplicity	7.9 (8.1)	10.2 (9.0)	20.5 (21.4)	10.4 (10.9)	7.1 (7.5)
CC 1/2^b^	1 (0.88)	-	-	1 (0.914)	0.999 (0.894)
Refinement					
Rcryst^c^	22.1	-	-	20.5	21.3
Rfree^c^	26.7	-	-	26.5	27.3
Overall B factor (A^2^)	60.8	-	-	80.1	57.1
RMS deviation					
Bond Length (Å)	0.016	-	-	0.021	0.019
Bond Angles (°)	2.170	-	-	2.201	2.197
PDB code	4JVC	-	-	4JVD	4JVI
Ramachandran plot					
Most favoured	197	-	-	197	196
Allowed	4	-	-	4	5

* Values in parentheses are for highest-resolution shell.

a R_merge_ = Σh Σi|*I*i(h)−<*I*(h)>/|Σh Σi *I*i(h), where *I* is the observed intensity and <*I*> is the average intensity of multiple observations from symmetry-related reflections calculated with XDS.

b Correlation co-efficient value calculated using XDS to determine the resolution cutoff.

c All values calculated using REFMAC. R_work_ = Σh||Fo|h−|Fc|h|/Σh|Fo|h, where Fo and Fc are the observed and calculated structure factors, respectively. R_free_ computed as in R_work_, but only for (5%) randomly selected reflections, which were omitted in refinement.

The PqsR^CBD^ structure has one molecule in the crystallographic asymmetric unit. Analytic gel filtration was performed with a calibrated Superdex 75 10/300 column revealing the recombinant PqsR^CBD^ eluted close to the 43 kDa marker (ovalbumin) and above the 29 kDa marker (carbonic anhydrase) (**Figure S1A**). As the calculated monomer molecular weight for PqsR^CBD^ is 22.7 kDa this indicates a dimeric species is present in solution. This is in agreement with the gel filtration data for the PqsR^CBD^ reported by Xu *et al.* [34].

The topology of the PqsR^CBD^ structure is illustrated in **Figure 3B** showing two subdomains (CBDs I and II) connected by an antiparallel β-sheet termed the hinge region as observed in related LTTR structures of BenM and CatM [35], [36]. Overall, CBDI is similar to other LTTRs with the exception of helix α2 which is shorter in PqsR. In CBDII a number of large changes are observed as a helix present in BenM between strands β5 and β6 is absent and replaced by an irregular loop structure (L1) lying at the junction of the two subdomains. The hinge region also adopts a different conformation in PqsR^CBD^ and the β4, β8 antiparallel strands are repositioned closer to the C-terminal helix. These two changes of a helix removal and the repositioning of the hinge region effectively open up the CBDII structure to form a large hydrophobic pocket occupied by two MPD molecules (**Figures 3C and 3D**).

MPD molecule 1 (MPD1) is highly buried, surrounded by aliphatic residues (Leu, Ile, Ala) from both sub-domains and a water mediated interaction with the carbonyl group of Ile236 at the bottom of the pocket (termed the B pocket, **Figure 3D**). MPD2 is predisposed closer to the surface in CBDII interacting with the side chains of Tyr258 and Val211 (the A pocket, **Figures 3C and 3D**).

We examined the crystal lattice and a large dimer interface was observed with a buried surface area of 1232 Å^2^ (topology shown in **Figure 3E**). A feature of the dimer is that it presents the two CBDII pockets on the same surface by aligning the two hinge regions which interact at either end of strands β4 and β8 forming contacts. At the centre, the β4 side chain Lys266 is fully extended forming a salt bridge to Glu259 close to the dimer axis. Further interfacial contacts occur between helices α3 and α6 at either end of the dimer (**Figure 3E**). A second feature is the two interlocking loops which form a lid partially covering the MPD molecules (lid loops L1 and L2 shown in red and blue respectively in **Figure 3F**). The pocket in each monomer connects across the dimer interface via a narrow channel shown as mesh in **Figure 3E**.

A second crystallographic dimer interface occurs in the PqsR lattice which buries a smaller surface area (614 Å^2^). This involves an antiparallel β-sheet formed by strand β2 as well as antiparallel packing of the two N-terminal α1 helices (**Figure S1B**). One side of the interface consists of hydrogen bonds from the β-sheet and sidechain-sidechain interactions from symmetry related Ser123 side chains (**Figure S1 C and D**). The residue Ser112 side chains from helix α1 form a similar side chain hydrogen bond close to the dimer axis. In the centre, hydrophobic contacts come from residues above and below the β-sheet (**Figure S1 C and D**).

### PqsR Co-inducer Interactions

**Figure 4:**
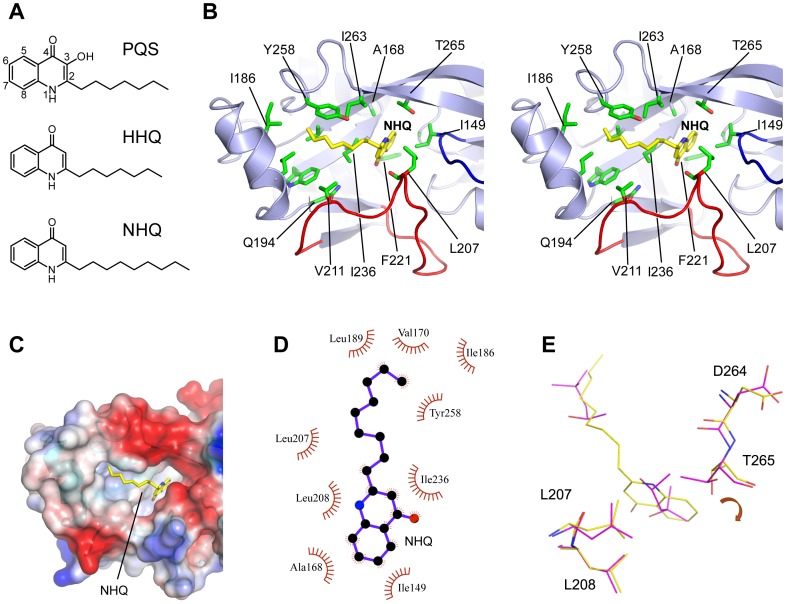
2-Alkyl-4-quinolone interactions with PqsR. (**A**) Structures of 2-heptyl-3-hydroxy-4(*1H*)-quinolone (PQS), 2-heptyl-4(*1H*)-quinolone (2-heptyl-4-hydroxyquinoline, HHQ) and 2-nonyl-4(*1H*)-quinolone (2-nonyl-4-hydroxyquinoline, NHQ); (**B**) A stereo diagram of the topology of the PqsR^CBD^-NHQ complex showing the ligand as stick (yellow). The quinolone ring is buried within the B pocket and the alkyl chain bound to the more surface accessible A pocket; (**C**) Charge surface representation of the PqsR^CBD^ showing the hydrophobic pocket occupied by NHQ. The colours represent acidic (red) and basic (blue) residues; (**D**) Ligplot schematic diagram showing PqsR hydrophobic contacts with NHQ (within a 3.9 Å radius); (**E**) Superposed PqsR^CBD^-NHQ (yellow) and PqsR^CBD^-MPD (purple) structures shown as stick.

We next sought to determine how the PqsR^CBD^ recognises a naturally occurring AQ co-inducer. As the PqsR^CBD^ lattice has a high solvent content (72%) we performed crystal soaking experiments with the most active native ligands produced by *P. aeruginosa* namely HHQ, NHQ (C9-HHQ), PQS and C9-PQS (**Figures 2** **and** **4A**) as well as shorter carbon chain length derivatives. For the majority of these experiments, a calculation of difference (Fo-Fc) maps resulted in the characteristic electron density for two MPD molecules occupying the PqsR^CBD^ pocket and no evidence of a bound ligand (**Figure S2A**). By contrast the soaking of NHQ for 24 h resulted in elongated and connected electron density spanning the two sub-pockets (**Figure S2B**). The electron density in the deeper B pocket was observed to be planar in shape and model building allowed fitting of the quinolone moiety of NHQ in one unique orientation. The alkyl chain can be modelled extending into the remaining cigar shaped electron density to occupy the A pocket. The Tyr258 side chain pins the alkyl chain down against side chains from residues Ile186, Val170, Leu189, Ile236 from the bottom and sides of the A pocket forming a comfortable fit (**Figures 4B and 4C**). The bicyclic ring structure is enclosed on either side in the B pocket by contacts from Leu207, Leu208 and Ile236. From above, contacts come from Ile149 and Ala168 and from below the sidechain of Phe221 (**Figure 4B**).

The fit of the bicyclic ring into the B pocket is not precise and beneath the carbonyl and in front of the aliphatic ring two vacant sub-pockets are present. A surprising feature of the structure is that the interactions are all hydrophobic with the absence of any hydrogen bonds or electrostatic interactions to the NHQ carbonyl oxygen or NH group of the bicyclic ring (**Figure 4D**). Although the interactions with NHQ are exclusively hydrophobic, modelling of the additional OH present in the alternative co-inducer, PQS, reveals this group could potentially form an additional contact through a hydrogen bonding interaction with the carbonyl group of Leu207.

Superposition of the PqsR^CBD^-NHQ with the PqsR^CBD^-MPD structure reveals subtle local conformational changes in the binding pocket (**Figure 4E**). The Thr265 side-chain rotates by 180° to make a direct contact with NHQ and a concomitant 0.7 Å movement of the main chain affects a similar movement in the position of the adjacent residue Asp264 in the hinge region affecting strands β4 and β8 (**Figure 4E**).

### Probing PqsR^CBD^ Ligand Binding by Site-Directed Mutagenesis

**Figure 5:**
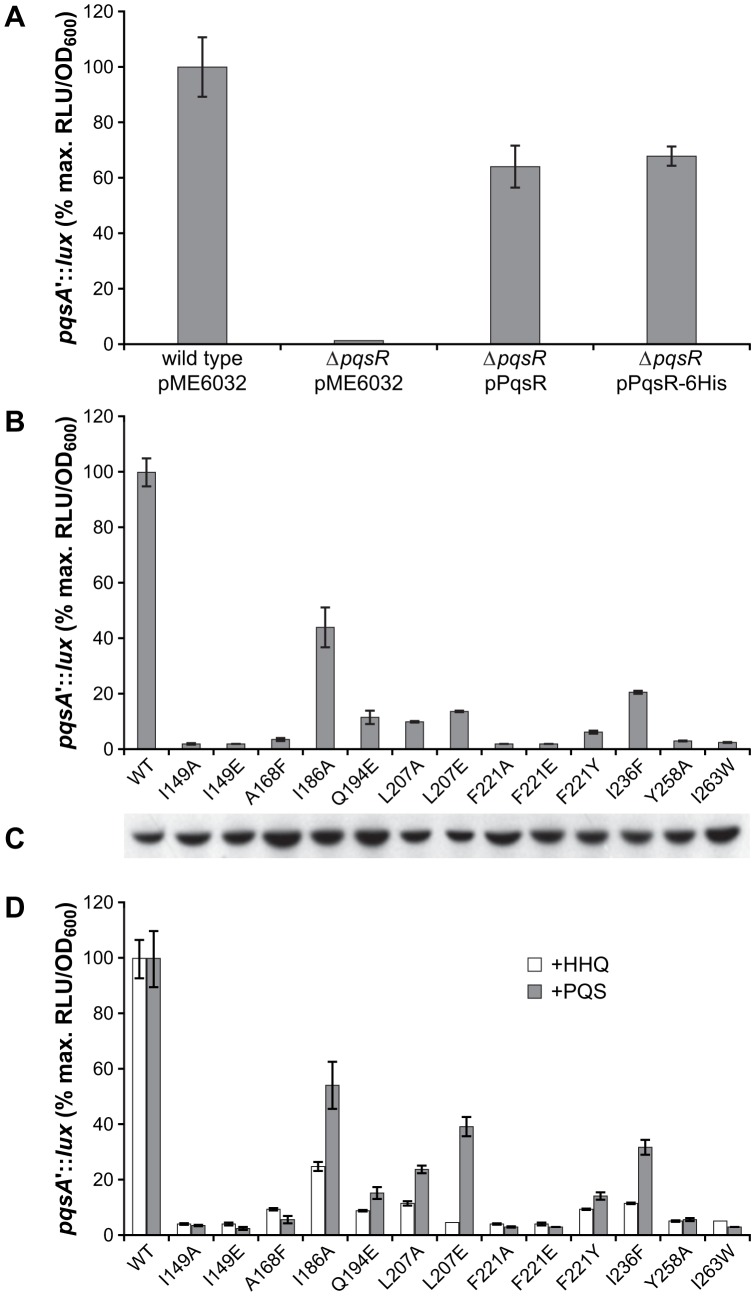
Response of PqsR^CBD^ ligand binding site mutants to AQs. (**A**) PqsR-6His is functional in *P. aeruginosa*. The *pqsR* gene with or without a 6His tag on pME6032 was introduced into a *P. aeruginosa pqsR* deletion mutant (Δ*pqsR*) containing a chromosomal miniCTX::*pqsA'-lux* reporter gene fusion. Relative PqsR activity was determined as a % of the maximum bioluminescence produced by the miniCTX::*pqsA'-lux* reporter fusion in a wild type *P. aeruginosa* PAO1 background carrying the empty pME6032 vector; (**B**) Light output from the *P. aeruginosa* Δ*pqsR* miniCTX::*pqsA'-lux* strain transformed with one of each of the 13 site-specific mutations introduced into the PqsR^CBD^ ligand-binding pocket. Bioluminescence is presented as % of *pqsA* promoter activity with respect to PAO1 Δ*pqsR* miniCTX::*pqsA'-lux* expressing the PqsR-6His protein (WT); (**C**) Western blot analysis confirming expression of each of the PqsR-6His mutant proteins; (**D**) Light output from the *P. aeruginosa* Δ*pqsA* Δ*pqsH* Δ*pqsR* miniCTX::*pqsA'-lux* strain transformed with either the gene coding for PqsR-6His or one of the 13 site-specific mutants and supplemented with either HHQ or PQS (40 µM). Bioluminescence is presented as % of *pqsA* promoter activity with respect to the *P. aeruginosa* Δ*pqsA* Δ*pqsH* Δ*pqsR* miniCTX::*pqsA'-lux* strain expressing PqsR-6His.

To probe the contribution of the key PqsR^CBD^ amino acid residues identified above (**Figure 4B**) to AQ binding, 13 site-specific substitutions were introduced into a 6His-tagged PqsR which retains activity in *P. aeruginosa* (**Figure 5A**). PqsR mutant functionality was evaluated by (i) Western blot to confirm PqsR expression (**Figure 5C**) and (ii) the ability to restore *pqsA* expression in the *P. aeruginosa* Δ*pqsR* miniCTX::*pqsA'-lux* reporter strain (**Figure 5B**). Each of the *pqsR* mutations altered *pqsA* promoter activity with mild reductions observed for three mutations Ile186Ala, Ile236Phe and Leu207Glu which exhibited 44.1%, 20.5% and 13.7% of the wild-type PqsR-6His, respectively (**Figure 5B**). In the PqsR^CBD^ structure, Ile186 is positioned at the far end of the A pocket and substitution by Ala removes the side chain atoms which contact the end of the alkyl chain; a loss of this contact would be predicted to reduce the interaction with the A pocket. Ile236 is positioned at the bottom of the A pocket lying at a boundary with the B pocket. It is fully buried by bound NHQ in the complex structure making contacts between its side chain atoms and the planar surface of the bicyclic ring. Mutation to Phe would be predicted to disrupt binding by introducing extra volume and changing the shape of the pocket. Leu207 is positioned on the side of the B pocket and interacts with NHQ at the junction between the alkyl chain and the bicyclic ring through its terminal side chain atom. The Leu207Ala and Leu207Glu mutations both result in a ∼10% response compared with wild type indicating that the altered size and charge similarly affect optimal ligand contacts. An almost complete loss of activity (<2% of that of the wild type) is observed for four mutations (Ile149, Phe221, Tyr258, Ile263). As we are unable to purify a soluble form of the full length wild type or mutant PqsR variants we could not rule out whether these four mutations have an effect by disrupting protein folding rather than ligand binding. Furthermore, we also observed that the PqsR^CBD^ protein precipitates in the presence of its highly hydrophobic ligands even at dilute protein concentrations (a finding also noted by Xiao *et al.*, [21]) making alternative biophysical approaches difficult.

Using a *P. aeruginosa* Δ*pqsA* Δ*pqsH* Δ*pqsR* triple mutant which does not produce endogenous AQs and containing a chromosomally-integrated miniCTX::*pqsA'-lux* reporter, we examined the response of the PqsR-6His variants to PQS and HHQ. The data presented in **Figure 5D** shows that the variants exhibited reduced responses to PQS and HHQ, consistent with the data in **Figure 5B**. However, the degree of response was significantly different for the two co-inducers, with PQS giving a consistently higher response, e.g. the Leu207Glu mutation responded much more poorly to HHQ (5%) than to PQS (39%). This observation is consistent with the notion that the additional 3-OH group of PQS can potentially form a hydrogen bond to the main chain carbonyl of Leu207 and thus it may not rely as heavily as HHQ/NHQ on the side chain interaction with Leu207 which is altered in this mutant.

### Synthesis and Evaluation of QZNs as PqsR Antagonists

**Figure 6:**
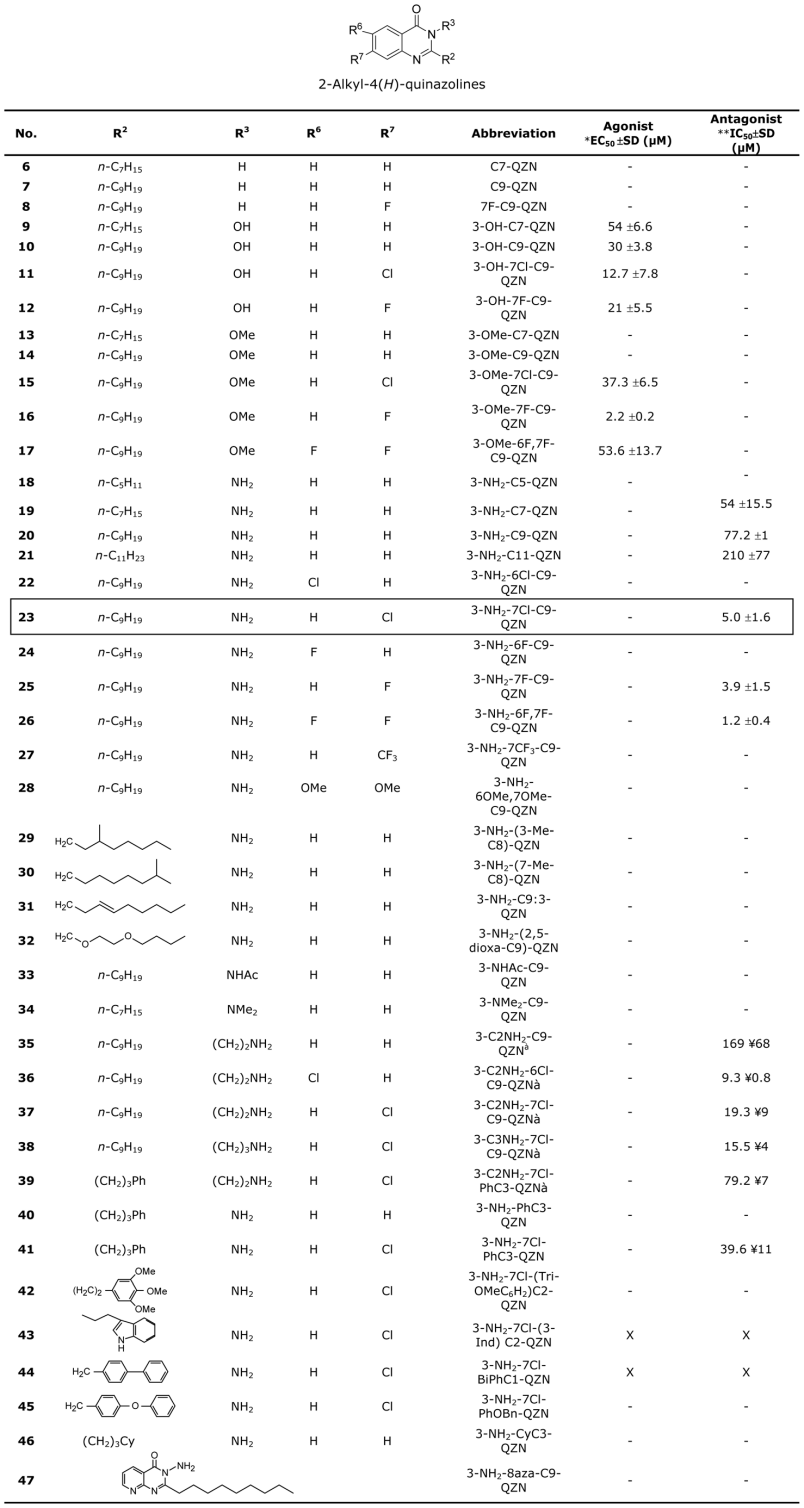
Activation and inhibition of PqsR in *P. aeruginosa* by the 2-alkyl-4(3*H*)-quinazolinones. *EC_50_ determined in a *P. aeruginosa* Δ*pqsA* miniCTX::*pqsA'-lux* strain; **IC_50_ determined in a *P. aeruginosa* wild type strain incorporating a miniCTX::*pqsA'-lux* fusion; – no activity; **^‡^**compounds exhibited growth inhibition; X compounds did not dissolve in MeOH at a workable concentration; 3-NH_2_-7Cl-PhOBn-QZN (46) is barely soluble.

To conserve the steric requirements for optimal ligand/receptor interactions, antagonists have frequently been discovered through structural modification of native agonists. Hence we used the closely related 2-alkyl-4(3*H*)-quinazolinone (QZN) system as a template to probe structure-activity relationships (SARs). We focused on QZN analogues with C7 or C9 alkyl side chains as AQ congeners with C5 or C11 have little activity ([23]). A series of 42 variously substituted QZNs (**Figure 6**) was synthesized and characterized as described in **Text S1** and the corresponding EC_50_s and IC_50_s for each compound determined via dose–response curves generated using PqsR-dependent *P. aeruginosa* miniCTX::*pqsA'-lux* reporter gene fusion assays. For comparative purposes, **Figure 2** summarizes the EC_50_ data obtained for PQS, HHQ and their corresponding C9 congeners, NHQ and C9-PQS in both *P. aeruginosa* Δ*pqsA* and *P. aeruginosa* Δ*pqsAH* mutant backgrounds since both HHQ and NHQ can be converted to the corresponding 3-hydroxy compound by the mono-oxygenase, PqsH [15]. **Figure 2** shows that the 4 co-inducers have similar EC_50_s in *P. aeruginosa*.

**Figure 7:**
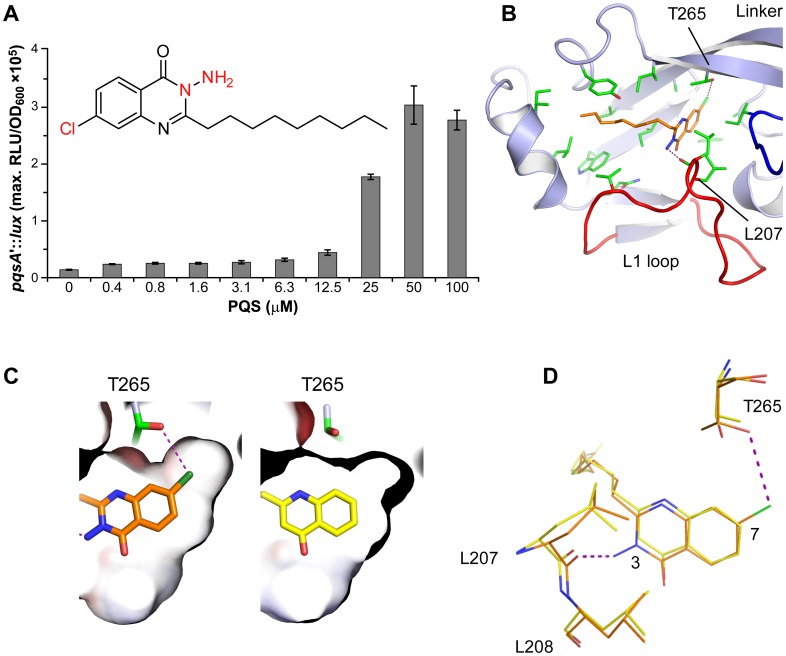
3-NH_2_-7Cl-C9-QZN is a competitive inhibitor of PqsR. (**A**) PqsR activity as reflected by the maximum bioluminescence produced by a miniCTX::*pqsA'-lux* fusion in a Δ*pqsA* mutant in the presence of 100 µM QZN (inset) and increasing concentrations of PQS; (**B**) Topology diagram of the PqsR^CBD^ ligand-binding site occupied by the QZN shown in stick (orange), with hydrogen bonds coloured purple; (**C**) Orientation of 4-quinolone ring (right panel) and 7Cl-substituted QZN ring (left panel) within the PqsR ligand-binding pocket. The 7Cl is accommodated within a crevice forming a hydrogen bond (dotted line) with Thr265 (left panel); (**D**) Superposed PqsR^CBD^-3-NH_2_-7Cl-C9-QZN and PqsR^CBD^-NHQ structures with residues shown as stick (orange and yellow respectively).

**Figure 8:**
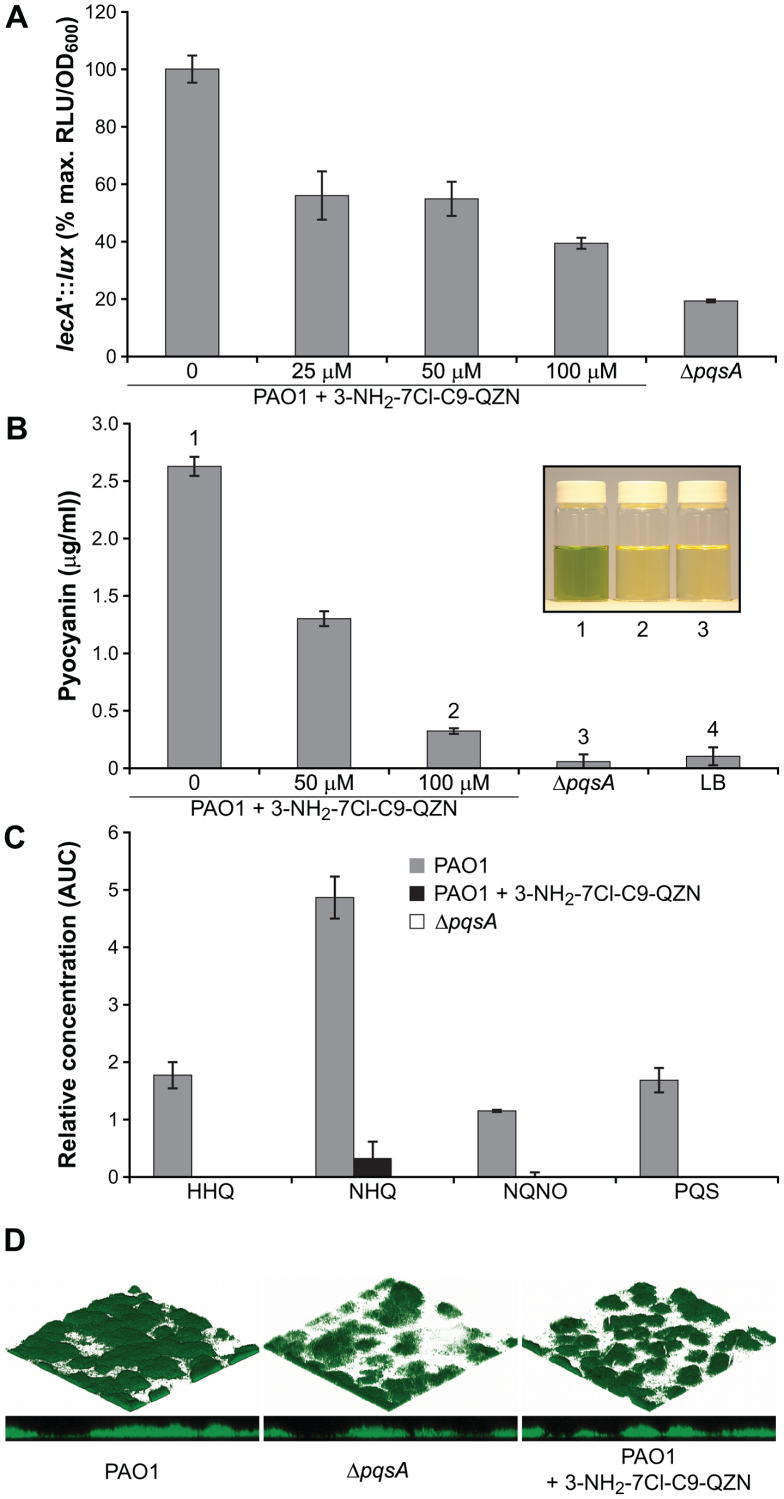
3-NH_2_-7Cl-C9-QZN inhibits AQ-dependent *P. aeruginosa* virulence factor production and biofilm development. (**A**) expression of *lecA* in *P. aeruginosa* PAO1 is inhibited by 3-NH_2_-7Cl-C9-QZN as reflected by a *lecA'-lux* chromosomal reporter fusion; (**B**) production of pyocyanin by *P. aeruginosa* PAO1 grown in the presence of 0, 50 or 100 µM 3-NH_2_-7Cl-C9-QZN and in a corresponding Δ*pqsA* mutant. The inhibition of pyocyanin production by 3-NH_2_-7Cl-C9-QZN is observed as an absence of green pigmentation in culture supernatants (inset; numbers correspond to columns); (**C**) Semi-quantitative analysis by LC-MS/MS of HHQ, NHQ, NQNO and PQS production by *P. aeruginosa* PAO1 grown in the absence or presence of 200 µM 3-NH_2_-7Cl-C9-QZN. The Δ*pqsA* mutant was used as a negative control; (**D**) Biofilm development is reduced in *P. aeruginosa* by a Δ*pqsA* mutation and following treatment of the wild type PAO1 strain with 3-NH_2_-7Cl-C9-QZN.

The SAR data for the QZNs is summarized in **Figure 6**. C7-QZN and C9-QZN (**Figure 6**, 7 and 8) which are 3-aza analogues of HHQ and NHQ respectively, and 7F-C9-QZN (**Figure 6**, 8), all of which lack a substitution in the 3-position, were devoid of agonist or antagonist activity. Hydroxylation at the 3-position of C7-QZN and C9-QZN gave compounds (**Figure 6**, 9 and 10) which were substantially weaker agonists than PQS. These agonist properties were substantially improved by the introduction of a halogen substituent at the 7-position of the carbocyclic ring as in 3-OH-7Cl-C9-QZN and 3-OH-7F-C9-QZN (**Figure 6**, 11 and 12). However the 3-methoxy variants, 3-OMe-C7-QZN and 3-OMe-C9-QZN (**Figure 6**, 13 and 14) were inactive unless halogenated at C-7 (**Figure 6**, 15 and 16). In this QZN series, 3-OMe-7F-C9-QZN (**Figure 6**, 16) was as potent an agonist as PQS. Introduction of a second fluorine to give 3-OMe-6F,7F-C9-QZN reduced potency by ∼25-fold (**Figure 6**, 17)

To explore the QZN SAR further and to identify an essential pharmacophore for antagonist activity, we replaced the 3-OH group with the isosteric 3-NH_2_ group in the above derivatives and synthesized 3NH_2_-C7-QZN (**Figure 6**, 19) and its alkyl chain altered variants (**Figure 6**, 20, 21, 29–32). None of these compounds were agonists. This was particularly interesting given that replacement of the 3-OH in PQS with 3-NH_2_ (**Figure 2**, 1 and 5a) results in a compound which is a more potent agonist than the natural ligand (EC_50_ 0.4±0.15 µM).

QZNs with branched chains (**Figure 6**, 29 and 30), unsaturation (**Figure 6**, 31) or with increased hydrophilicity (**Figure 6**, 32) were all inactive while compounds 19, 20 and 21 (**Figure 6**) were antagonists, the most potent being 3NH_2_-C7-QZN (**Figure 6**, 19; IC_50_ 54±15.5 µM). An attempt to further improve the activity via the introduction of a 6-Cl substituent in 3NH_2_-C9-QZN to yield 3NH_2_-6Cl-C9-QZN (**Figure 6**, 22) resulted in the complete loss of antagonist activity but gratifyingly, potency was greatly increased by a 7-Cl substituent (3-NH_2_-7Cl-C9-QZN; **Figure 6**, 23; IC_50_ 5±1.6 µM). Fluorine substituted derivatives (**Figure 6**, 25 and 26) were also antagonists, the most potent compound being 3-NH_2_-6F,7F-C9-QZN (IC_50_ 1.2±0.4 µM).

Introduction of an electron withdrawing group CF_3_ at C-7 as in 3-NH_2_-7CF_3_-C9-QZN or 8-aza as in 3-NH_2_-8aza-C9-QZN (**Figure 6**, 27 and 47 respectively) resulted in the complete loss of activity. The presence of electron donating methoxy substituents as in 3-NH_2_-6OMe,7OMe-C9-QZN (**Figure 6**, 28) also rendered the compound inactive. Further modification of the 3-NH_2_ group of the C9-QZNs by acetylation, dimethylation, or 2-aminoethylation (**Figure 6**, 33, 34 and 35 respectively) yielded only inactive or weak antagonists, the potency of which could be increased as before by the presence of a Cl at the 6 or 7 position of the carbocyclic ring (**Figure 6**, 36 and 37). The 3-(3-aminopropyl) compound (**Figure 6**, 38) only showed marginal improvement in activity. However these compounds (**Figure 6**, 35–38) also exhibited growth inhibitory activity and were therefore excluded from further work given that a primary objective was to obtain PqsR inhibitors which attenuate *P. aeruginosa* virulence without inhibiting growth.

Compounds 39–46 (**Figure 6**) represent further attempts to increase antagonist potency through modification of the alkyl side chain by synthesising derivatives where the alkyl chain is terminally substituted with aryl (**Figure 6**, 39–42), heteroaryl, biaryl or cyclohexyl groups (**Figure 6** compounds 43,44,45 and 46) respectively. These QZNs were all inactive apart from the phenyl substituted compounds 3-C2NH_2_-7Cl-PhC3-QZN and 3-NH_2_-7Cl-PhC3-QZN (**Figure 6**, 39 and 41) which were weak antagonists (IC_50s_ 79.2±2.7 µM and 39.6±11 µM respectively).

### Structure of the PqsR^CBD^-3-NH_2_-7Cl-C9-QZN Complex

To determine whether the QZN antagonists are competitive inhibitors which interact with the AQ-binding pocket, we first investigated whether inhibition of PqsR by 3-NH_2_-7Cl-C9-QZN could be overcome by increasing concentrations of PQS. The data are shown in **Figure 7A** which reveals that PQS above 25 µM competitively overcomes QZN-mediated PqsR inhibition. To investigate how the 3-NH_2_-7Cl-C9-QZN interacts with PqsR^CBD^ crystals were soaked in a solution of the compound. The resulting structure revealed the quinazolinone moiety is buried in the B pocket with the alkyl chain extending into the A pocket. The QZN molecule forms very similar hydrophobic interactions with the pocket noted for NHQ (**Figure 7B**). In addition, the Cl atom of the 3-NH_2_-7Cl-C9-QZN occupies the vacant sub pocket present in front of the aliphatic ring and forms a hydrogen bond with the side chain of Thr265 (**Figure 7C**). The 3-NH_2_ substituent forms a hydrogen bond to the main chain carbonyl oxygen of Leu207 (**Figure 7 B and D**). Superposition of this structure with the agonist NHQ-PqsR^CBD^ complex reveals differences resulting from the additional contacts made by the QZN (**Figure 7D**). This tilts the QZN bicyclic ring relative to NHQ affecting a subtle repositioning of the alkyl chain. The QZN interactions affect small changes in the main chain of the L1 loop and the 7-Cl atom induces a rotation of the Thr265 side chain rotamer by 90° to NHQ. In addition, the introduction of the 7Cl substituent into PQS to generate 7Cl-PQS resulted in an agonist which is ∼135 times more potent than PQS itself (**Figure 2**) providing further confirmation of the importance of the vacant sub-pocket adjacent to the Thr265 residue.

### QZN Antagonists as Anti-virulence Agents

The QZN antagonists of PqsR were identified on the basis of their inhibition of *pqsA* transcription through the competitive antagonism of the AQ-dependent activation of PqsR. To determine whether the QZNs could also inhibit the expression of target virulence genes such as *lecA* (which codes for the cytotoxic galactophilic lectin protein LecA, which also contributes to biofilm development; [37]) and *phzA1* (which codes for an enzyme involved in the biosynthesis of the redox-reactive phenazine pigment pyocyanin; [38]), we constructed *lecA-lux* and *phzA1-lux* reporter gene fusions integrated in the chromosome of wild type *P. aeruginosa* PAO1. Since lectin A and pyocyanin production depend on *pqsE* expression [16], which in turn requires the PqsR-dependent activation of the *pqsABCDE* operon, inhibitors of PqsR should result in the down-regulation of *lecA* and *phzA* expression. Compared with the control, the expression of *lecA* is reduced by 3-NH_2_-7-Cl-C9-QZN (**Figure 8A**). Similar results were obtained with the *phzA1* promoter for which 12.5 µM 3-NH_2_-7Cl-C9-QZN reduced activity by ∼50% (data not shown). In agreement with the findings for the *phzA1* promoter, pyocyanin levels were also substantially reduced in *P. aeruginosa* cultures treated with the QZN (**Figure 8B**). These data are consistent with a reduction in AQ levels and LC-MS/MS analysis of *P. aeruginosa* cultures grown in the presence of 3-NH_2_-7Cl-C9-QZN shows that the production of HHQ, NHQ, their corresponding *N*-oxides as well as PQS are reduced to very low levels (**Figure 8C**). Since AQ-dependent QS also contributes to biofilm maturation we examined the impact of 3-NH_2_-7Cl-C9-QZN on biofilm development under flow conditions using a microfluidics device. Representative confocal microscope images of the green fluorescent protein (GFP)-tagged *P. aeruginosa* wild type grown in the presence or in the absence of 3-NH_2_-7Cl-C9-QZN, and of the *P. aeruginosa* Δ*pqsA* mutant strain are shown in **Figure 8D**. In common with the Δ*pqsA* mutant, 3-NH_2_-7Cl-C9-QZN-treated wild type biofilms exhibited reduced surface area coverage. This is consistent with previous reports on reduced biofilm formation in *P. aeruginosa pqsA* mutants, primarily as a result of a reduction in the release of extracellular DNA, an important constituent of the extracellular matrix which is released via a process that requires PQS [39], [40].

## Discussion

Understanding the molecular recognition of AQs has important implications for gaining insight into the molecular basis of the PqsR receptor ligand and inhibitor interactions. We determined the crystal structure complex of the PqsR^CBD^ domain with native agonist and synthetic antagonist ligands. This revealed a core structure similar to that of other LTTR proteins incorporating two sub-domains (CBDI and CBDII). Among LTTR proteins, the CBD domains have the same overall topology despite little sequence similarity [36]. However, in contrast to other LTTRs, where a small primary ligand-binding pocket is located in the cleft between the two sub-domains [35], the PqsR ligand binding site is larger extending into CBDII as well as occupying a large B pocket between CBDI and CBDII (**Figure S3**). This pocket is partially covered by lid loops upon formation of a large dimer interface. A central antiparallel dimer organisation is common among LTTR crystal structures, however these are formed between the ‘cleft’ side of the monomer whereas the PqsR^CBD^ dimer is formed from the hinge region β-strands.

Crystal soaking experiments with NHQ revealed that this hydrophobic cavity within the PqsR^CBD^ constitutes the AQ-ligand binding site where the quinolone moiety is buried within the B pocket, the alkyl chain extending into the surface crevice of the CBDII A pocket. The PqsR^CBD^-NHQ complex is stabilised entirely by hydrophobic interactions and no electrostatic interactions are involved.

In *P. aeruginosa* reporter gene fusion assays, HHQ, PQS and their C9 congeners (NHQ and C9-PQS) each have similar EC_50_s (**Figure 2**; [23]) whereas the C11 congeners are inactive, consistent with the space constraints noted from the crystal structure. The importance of the amino acids observed to form the PqsR^CBD^ ligand binding site in the crystal structure was investigated using site-directed mutagenesis. Of the 13 residues mutated, all resulted in a major reduction in activity except for the I186A mutation which is located at the edge of the A pocket and retained ∼44% activity. Interestingly the increased A pocket space available as a consequence of the I186A replacement did not increase the activity of the C9 (i.e. NHQ) or the C11 congeners of HHQ (data not shown).

PQS and its biosynthetic precursor HHQ (as well as their C9 congeners) can both act as activating PqsR co-inducers. Using a *P. aeruginosa* Δ*pqsA* Δ*pqsH* double mutant which cannot convert exogenously supplied HHQ to PQS, we observed a ∼3-fold higher induction of the *pqsA* promoter by PQS when compared with HHQ (**Figure 2**). This is much lower than the 100-fold higher induction reported by Xiao *et al.* [21] using a different *P. aeruginosa* strain and reporter assay. PQS has also been reported to potentiate the binding of recombinant PqsR in crude *E. coli* lysates to DNA more effectively than HHQ [20], [21]. The greater efficiency of PQS over HHQ may be a consequence of the increased H-bonding opportunities with the Leu207 carbonyl afforded by the presence of the 3-OH substituent. In this context it is perhaps noteworthy that the L207A and L207E PqsR mutants are significantly more responsive to PQS than to HHQ. Although we were unable to obtain a structure for the PQS complex with PqsR^CBD^, it is anticipated that as both the QZN and NHQ PqsR^CBD^ structures superpose accurately, that their similarity in chemical structure with PQS will result in a similar binding. Nevertheless, the primary advantage of introducing a 3-OH substituent in HHQ is probably not enhancement of PqsR activation but to confer additional functionalities since PQS, unlike HHQ, induces outer membrane microvesicle formation [24] and is a potent iron chelator [14].

LTTRs assemble into oligomers (tetramers, and in one case an octamer) which is the functional DNA-binding structure affecting DNA bending and the recruitment of RNA polymerase [41], [35], [29]. The degree of DNA bending is determined by the presence or absence of the co-inducer which causes a conformation change in the LTTR resulting in a relaxation of the degree of DNA bending [29], [42]. In the redox switch LTTR protein OxyR, the reduced form is a tetramer and the activated, oxidised form undergoes a conformational change in CBDII affecting a CBDI interface within the tetramer. This is thought to position the DNA binding domains appropriately for interaction with DNA and the RNA polymerase [41]. The nature of the quaternary arrangement formed by PqsR in complex with DNA and co-inducer has yet to been elucidated. Progress towards this goal is hampered by the inability to prepare full-length recombinant PqsR receptor heterologously expressed in *E. coli*, an important goal for any future studies in this area.

A comparison of the PqsR^CBD^ structure with other LTTRs reveals that the L1 loop in CBDII occupies the same region of the topology as key residues required for co-inducer mediated conformational changes [41], [35], [32]. In OxyR, the region equivalent to the PqsR L1 loop switches conformation upon oxidation or reduction and this is linked to a re-organisation of the tetramer interface [41]. In TsaR and CatM, co-inducer interactions and conformational switching are mediated by an α-helix occupying the same position as the PqsR CBDII L1 loop [35], [32]. Thus co-inducer affected changes in the region of the L1 loop may be the first steps on the pathway to activation of the receptor and hence gene expression.

In our search for potent PqsR antagonists as novel therapeutics, we focused on the QZN system since sterically it is closely related to the natural AQ ligands. We systematically varied the nature and size of the substituents at the 2 and 3 positions in the heterocyclic ring as well as positions 6 and 7 in the carbocyclic ring of the QZN structure to deliver a range of analogues with pharmacophores that may have the desired stereo-electronic properties for antagonist activity. Thus a total of 46 QZNs (**Figure 6**) were synthesised, characterised (see supplemental **Text S1**) and assayed for their agonist and antagonist activities in a whole *P. aeruginosa* bacterial cell assay. The QZN analogues lacking substitution at C-3 (**Figure 6**, 5–7) were inactive although their corresponding AQs, HHQ and NHQ (**Figure 2**, 2 and 3) displayed strong agonist activity. Substitution at C-3 with OH gave analogues 8 and 9 (**Figure 6**) which like the corresponding PQS and C9-PQS (**Figure 2**, 1 and 4) were partial agonists. The 3-methoxy derivatives 12 and 13 (**Figure 6**) were weak antagonists but were totally devoid of agonist activity. Surprisingly, substitution with a halogen in the C-7 position in the carbocyclic ring reversed the activity and indeed 3-OMe-7F-C9-QZN (**Figure 6**, 15) had potent agonist activity (EC_50_ 2.2 µM) comparable with that of the natural ligands. The introduction of a second halogen at C-6 has the opposite effect and consequently 3-OMe-7F-C9-QZN (**Figure 6**, 16) is a much weaker agonist.

The QZN compounds synthesized fell in two distinct groups with 3-OH (and OMe) QZNs generally behaving as agonists and the 3-NH_2_ QZNs as competitive antagonists in whole bacterial cell assays of PqsR activity. Additionally, an alkyl chain of 9 carbons at C-2 in the heterocyclic ring and a halogen at C-7 in the carbocyclic ring are essential for optimum activity in both series. 3-NH_2_-7Cl-C9-QZN (IC_50_ 5.0 µM), 3-NH_2_-7F-C9-QZN (IC_50_ 4.3 µM) and 3-NH_2_-6F,7F-C9-QZN (IC_50_ 1.7 µM) were the most potent antagonists discovered in our studies. Of these, 3-NH_2_-7Cl-C9-QZN was shown to be an effective inhibitor of AQ signaling by antagonizing AQ biosynthesis, virulence gene expression, pyocyanin production and biofilm development. Furthermore, the preference for a halogen at C-7 over C-6 is clearly apparent from the PqsR^CBD^/3-NH_2_-7Cl-C9-QZN complex structure (**Figure 7C**) where additional H-bonding opportunities for the 7Cl substituent are afforded by the pocket formed by the Thr265. Thus it is probable that 3-NH_2_-7Cl-C9-QZN binds more strongly to the PqsR^CBD^ than the native ligands via the strengthened electrostatic interactions between 3-NH_2_ substituent, the water molecule and Leu207 backbone carbonyl in conjunction with additional H-bonding between the 7-Cl and Thr265. However this will require experimental verification.

Remarkably, the simple replacement of the 3-OH with 3-NH2 in the 7Cl-substituted QZNs converts the compound from a potent agonist to a potent antagonist (**Figure 6**, 11 and 23). The importance of this small but significant finding is that the replacement of the PQS 3-OH with 3-NH_2_ does not affect the switch from agonist to antagonist (as both compounds are strong agonists with similar EC_50_s (**Figure 2**; 1.9 µM compared with 0.4 µM). This indicates that for the QZNs compared with the AQs, the stereo-electronic consequences of the additional QZN ring nitrogen are profound in terms of PqsR activation.

In addition to the preliminary SAR studies for PQS agonists [23], [43], PqsR antagonists have recently been described by Klein *et al.* [44] and by Lu *et al.* [45]. The former identified substituted benzamides lacking extended alkyl chains which bind to the PqsR^CBD^ and exhibit relatively weak agonist or antagonist activities by using (±)-trans-U50488 as a template for rational design since this κ-opioid receptor agonist was reported to stimulate *pqsA* transcription [46]. The biological evaluation of these compounds and those of Lu *et al.* [45] have mostly been undertaken using a heterologous *E. coli*-based PqsR-dependent transcriptional reporter which is more sensitive to PQS than *P. aeruginosa* [23], [43]) probably as a consequence of the numerous efflux pumps present in the latter. HHQ analogues with electron-withdrawing C6 substituents (nitrile (–CN), triofluoromethyl (–CF_3_) and nitro (–NO_2_)) were potent antagonists in *E. coli* whole cell assays with EC_50_s in the nanomolar range [45]. However, at the concentrations tested, they failed to reduce PQS production in *P. aeruginosa* although pyocyanin levels were lower after treatment with the 6-CF_3_ analogue. Interestingly, the 7-CF_3_-substituted HHQ, in contrast to the 6-CF_3_ analogue, was devoid of antagonist activity and retained agonist activity at about 50% that of HHQ [45]. In the *E. coli*-based assay, PQS analogues with Cl substitutions at 5, 6, 7 or 8 all exhibited similar activities to PQS [43]. However, in *P. aeruginosa*, 7-Cl-PQS is ∼135× more potent than PQS (**Figure 2**), a finding consistent with the PqsR^CBD^/3-NH_2_-7Cl-C9-QZN complex structure which revealed that a 7-Cl substituent can occupy a pocket and form an H-bond with the side chain of Thr265.

Competitive PqsR antagonists such as 3-NH_2_-7Cl-C9-QZN bind within the PqsR^CBD^ ligand binding pocket in the same orientation as agonists such as NHQ and form additional hydrogen bonds to the side chain OH of Thr265 and the main chain carbonyl of Leu207. Since LTTR agonists stimulate transcription of target genes through changes in the orientation of the DNA and ligand-binding domains [32] this would suggest that the binding of 3-NH_2_-7Cl-C9-QZN, although likely to be tighter than an agonist, is not productive and either maintains the PqsR conformation in the same state as the unbound protein or drives the formation of a different, but inactive, conformation. The latter mechanism has been reported for an antagonist that binds in place of the native *N*-acylhomoserine lactone ligand to the LuxR family protein CviR and forces the transcriptional regulator to adopt a conformation incompatible with high affinity DNA operator binding [47]. Here we have found that superposition of the PqsR^CBD^-3-NH_2_-7Cl-C9-QZN and PqsR^CBD^-NHQ complexes results in subtle changes which tilt the bicyclic ring of the QZN relative to that of NHQ so the QZN interaction is not productive for PqsR activation.

Taken together this work has increased our knowledge of the molecular recognition of ligands by PqsR, demonstrated how the simple and subtle exchange of two isosteres (OH for NH_2_) within a co-inducer molecule can effectively switch virulence gene expression on or off and provided a template structure for the development of QZNs as novel therapeutics which control infection through attenuation of *P. aeruginosa* virulence.

## Materials and Methods

### Bacterial Strains and Growth Conditions

The *E. coli* and *P. aeruginosa* strains used in this study (**Table S1**) were grown in Lysogeny broth (LB) at 37°C. For AQ quantification, *P. aeruginosa* was grown in minimal medium [48]. When required for plasmid maintenance in *E. coli* (pET28a derivatives) or *P. aeruginosa* (pME6032 derivatives), kanamycin (50 µg/ml) or tetracycline (125 µg/ml) were respectively added to the growth medium.

### Mutant Construction

The *P. aeruginosa* Δ*pqsR* in-frame deletion mutant and the triple Δ*pqsA* Δ*pqsH* Δ*pqsR* mutant were constructed in the PAO1 parent and Δ*pqsA* Δ*pqsH* mutant [14] respectively using the pDM4Δ*pqsR* plasmid. The upstream and downstream fragments of *pqsR* were amplified by PCR from PAO1 chromosomal DNA using the primers pairs FW*pqsR*Up-RV*pqsR*Up and FW*pqsR*Down-RV*pqsR*Down, respectively (**Table S2**), introduced into pDM4 [49] and the resulting Δ*pqsR* mutants obtained by allelic exchange [50]. The *pqsA* promoter fused to the *luxCDABE* reporter operon was introduced into both the Δ*pqsR* and Δ*pqsA* Δ*pqsH* Δ*pqsR* mutants using the miniCTX*pqsA*-*lux* plasmid as described previously [23].

For complementation assays, the *pqsR* gene with or without a C-terminus 6xHis coding sequence (*pqsR-6H*) was amplified by PCR from chromosomal DNA with primer pairs FW*pqsR*-RV*pqsR* or FW*pqsR*-RV*pqsR-6H*, respectively (**Table S2**), and cloned by EcoRI-SacI digestion into pME6032 [51]. Site-directed mutations were generated in *pqsR-6H* using the splicing by overlap extension PCR method [52]. Briefly, in the first step two distinct PCRs (PCR-1 and PCR-2) were carried out for each *pqsR-6his* derivative mutant, using chromosomal *P. aeruginosa* DNA as a template. Each PCR-1 was performed with the forward primer FW*pqsR* and with a mutagenic reverse primer carrying the mutation in the desired codon (**Table S2**). Each PCR-2 was performed with a mutagenic forward primer complementary to the reverse primer utilized in the corresponding PCR-1 (**Table S2**) and with primer RV*pqsR-6H*. In the second PCR step products obtained from PCR-1 and PCR-2 for each mutation were spliced together using the FW*pqsR* and RV*pqsR-6H* primers. The mutated *pqsR-6H* variants were cloned by EcoRI/SacI digestion in pME6032 and verified by DNA sequencing. The expression of each of the PqsR variants was confirmed by Western blot analysis using a mouse anti-6xHis antibody (1∶1,000; Sigma-Aldrich, St. Louis, MO, USA).

### Protein Expression, Purification and Crystallization

For overexpression of the PqsR^CBD^, the regions corresponding to the PqsR C^94-332^ and PqsR C^94-294^ CBD were amplified by PCR and cloned into pET28a. The recombinant plasmids were introduced by transformation into *E. coli* Rosetta 2 (DE3) and grown at 37°C to an OD_600_ 0.8 prior to induction with IPTG (1 mM) at 20°C for 16 h. After harvesting by centrifugation, the bacteria were lysed by sonication, centrifuged and filtered to remove cellular debris prior to nickel affinity column purification and elution with an imidazole gradient (0 to 1 M). The 6xHis tag which included the thrombin cleavage sequence was removed from the PqsR^CBD^ proteins using thrombin (Novagen; enzyme/substrate ratio 1∶1000 for 24 h). Final purification was achieved by gel filtration using a Superdex 75 16/60 gel filtration column, with a mobile phase consisting of 20 mM Tris-HCl, 150 mM NaCl, pH 7.4. Both constructs yielded approximately 25 mg PqsR^CBD^/litre of culture as confirmed by SDS-PAGE. The same strategy was used to obtain the PqsR^94-332^ selenomethionine-labelled protein after transforming the *E. coli* methionine auxotroph strain B834 (DE3) with pPqsR^94-332^ and growing the recombinant strain in selenomethionine medium. A protein concentration of 25 mg/ml was used for 96-well crystal screening (Qiagen kits) and crystals were obtained for PqsR^94-332^ and from several conditions using only the MPD suite (Qiagen) after 24 h at 19°C. Optimized conditions in 24-well sitting drop plates containing a reservoir of 100 mM trisodium citrate pH 6.0, 200 mM ammonium acetate and 3% v/v MPD and identical crystals grew with the shortened PqsR^94-309^ construct.

Analytical gel filtration was performed with a Superdex 75 10/300 column equilibrated with running buffer of 20 mM Tris-HCl, 150 mM NaCl, pH 7.4. The standards used were ovalbumin (43 kDa), carbonic anhydrase (29 kDa) and ribonuclease A (13.7 kDa).

### Data Collection and PqsR^CBD^ Structure Determination

Crystals were transferred to a solution with cryoprotectant of 25% MPD and cryo-cooled for collection of diffraction data. The detailed method for PqsR^CBD^-MPD structure determination is outlined in **Text S1**. Native and derivative datasets were collected at beamline IO4 of the Diamond synchrotron and data was processed using XDS and reduced with the CCP4 suite (statistics are shown in **Table 1** together with the description of the SIRAS structure determination for the PqsR^CBD^-MPD structure). PqsR ligands were dissolved in 100% MPD or in a 1∶1 mixture of MPD and isopropanol to give a concentration of 20 mM. When added to recombinant PqsR^CBD^ even low concentrations of these compounds resulted in heavy precipitation. The soaking of PqsR^94-309^ crystals was carried out for 24–48 h with ligands at 5–10 mM. Soaking experiments with HHQ, NHQ, PQS, C9-PQS and shorter chain analogues were carried out and in each case crystals were transferred to a solution with cryoprotectant of 25% MPD and cryo-cooled for collection of diffraction data. Datasets were collected at beamline IO2 of the Diamond synchrotron and data were processed using XDS and reduced with the CCP4 suite (**Table 1**). Rigid body refinement (REFMAC) was carried out to adjust for small changes in cell dimension and 2Fo-Fc and Fo-Fc electron density maps were calculated using the CCP4 suite. Additional electron density was observed for NHQ and 3-NH_2_-7Cl-C9-QZN soaked crystals. Model building was carried out using COOT and refinement with REFMAC.

### AQ and QZN Synthesis

The AQs and QZNs listed in **Figures 2** **and** **6** were synthesised and characterised as described in the supplemental information provided in **Text S1**.

### Bioluminescence Reporter Gene Fusion Assays

The impact of the AQs and QZNs on PqsR-dependent gene expression in *P. aeruginosa* was evaluated using *lux*-based *pqsA*, *lecA* and *phzA1* promoter fusions (**Table S1**) in 96-well microtiter plates as described before [14],[23]. Bioluminescence and bacterial growth were quantified using a combined luminometer-spectrometer (Tecan GENios Pro). Where required, AQs or QZNs were added to reporter strains and EC_50_ or IC_50_ values were extracted from the sigmoidal dose–response curves obtained using Prism2 (Graphpad, San Diego, USA).

### AQ, Pyocyanin and Biofilm Analysis

The impact of 3-NH_2_-7Cl-C9-QZN on (a) AQ production was assayed by LC MS/MS after extracting bacterial cultures with acidified ethyl acetate [11]; (b) pyocyanin was quantified spectrophotometrically [16] and (c) biofilm development was examined using a Bioflux 200 microfluidics device (Fluxion Biosciences; in conjunction with GFP-labelled *P. aeruginosa* strains, [53]). All assays were performed in triplicate at least twice.

## Supporting Information

Figure S1 (**A**) Gel filtration chromatogram of PqsR^CBD^ (residues 94-332). Absorbance elution profiles are shown PqsR^CBD^ in blue overlapped with the elution profiles of protein standards (red) using a Superdex 75 10/300 analytical column. The standards include I - ovalbumin (43 kDa), II - carbonic anhydrase (29 kDa) and III - ribonuclease A (13.7 kDa). (**B**) The PqsR^CBD^ tetrameric arrangement observed in the crystal structure shown as topology with MPD shown as spheres. (**C**) A close up view of the PqsR^CBD^ tetramer interface shown as topology. Two views are shown related by a 180 degree rotation with hydrogen bonding interactions highlighted as dotted lines (orange) and key residues shown in stick. (**D**) End view of the interface shown in (**C**).
(PDF)

Figure S2 Electron density maps are shown for bound ligands displayed within a 2 Å radius. (**A**) Left panel shows the final refined 2.7 Å electron density map (blue) with SigmaA m2Fo-DFc coefficients and phases c of the PqsR-MPD structure contoured at 1.0 r.m.s (REFMAC, CCP4). The modelled MPD molecules are shown as stick and the figure was generated using PyMOL. The middle panel shows the same calculation with the atoms of the MPD excluded (simple omit map). The far right panel is calculated in the absence of any atoms from the ligand with the remaining coordinates utilised in a simulated annealing (SA) combined omit map protocol. The map was displayed in COOT used to generate the figure and contoured at 1.2 r.m.s. (**B**) Left panel shows the final refined 3.1 Å electron density map (blue) with SigmaA m2Fo-DFc coefficients and phases c of the PqsR-NHQ structure contoured at 1.0 r.m.s (REFMAC). The modelled NHQ molecule is shown as stick and the figure was generated using PyMOL. The middle panel shows the same calculation with the atoms of the NHQ excluded (omit map). The far right panel is the phenix SA omit calculation described in (**A**) with NHQ atoms omitted and contoured at 1.2 r.m.s. (**C**) Left panel shows the final refined 3.1 Å electron density map (blue) with SigmaA m2Fo-DFc co-efficients and phases c of the PqsR-3NH2-7Cl-C9QZN (QZN) structure contoured at 1.0 r.m.s (REFMAC). The modelled QZN molecule is shown as stick and the figure was generated using PyMOL. The right panel shows the phenix SA omit calculation as in (**A**) with the atoms of the QZN excluded.
(PDF)

Figure S3 Unique dimer organisation of PqsR. Cartoon diagrams of the central dimer organisation for LTTRs PqsR, OxyR (pdb: 1I69), BenM (pdb: 2F78) and TsaR (pdb: 3FXQ). β-strands are colored magenta and α-helices in cyan. β-strands involved in the hinge region are indicated by a blue arrow showing the centrally located hinge regions in PqsR compared to the peripheral location of hinge regions on OxyR, BenM and TsaR.
(PDF)

Table S1 Strains and plasmids used in this study.
(PDF)

Table S2 Oligonucleotides used in this study.
(PDF)

Text S1 Supplemental Materials and Methods and ^1^H NMR spectra.
(PDF)
