# Supplementary material for: Structural Basis for Native Agonist and Synthetic Inhibitor Recognition by the Pseudomonas aeruginosa Quorum Sensing Regulator PqsR (MvfR)
Source: PLoS Pathog. 2013 Jul 25;9(7):e1003508. doi: 10.1371/journal.ppat.1003508 (PMC3723537; doi:10.1371/journal.ppat.1003508)
Supplement: Figure S1 — (A) Gel filtration chromatogram of PqsRCBD (residues 94-332). Absorbance elution profiles are shown PqsRCBD in blue overlapped with the elution profiles of protein standards (red) using a Superdex 75 10/300 analytical column. The standards include I - ovalbumin (43 kDa), II - carbonic anhydrase (29 kDa) and III - ribonuclease A (13.7 kDa). (B) The PqsRCBD tetrameric arrangement observed in the crystal structure shown as topology with MPD shown as spheres. (C) A close up view of the PqsRCBD tetramer interface shown as topology. Two views are shown related by a 180 degree rotation with hydrogen bonding interactions highlighted as dotted lines (orange) and key residues shown in stick. (D) End view of the interface shown in (C). (PDF) [file ppat.1003508.s001.pdf]

Supplementary Figure S1

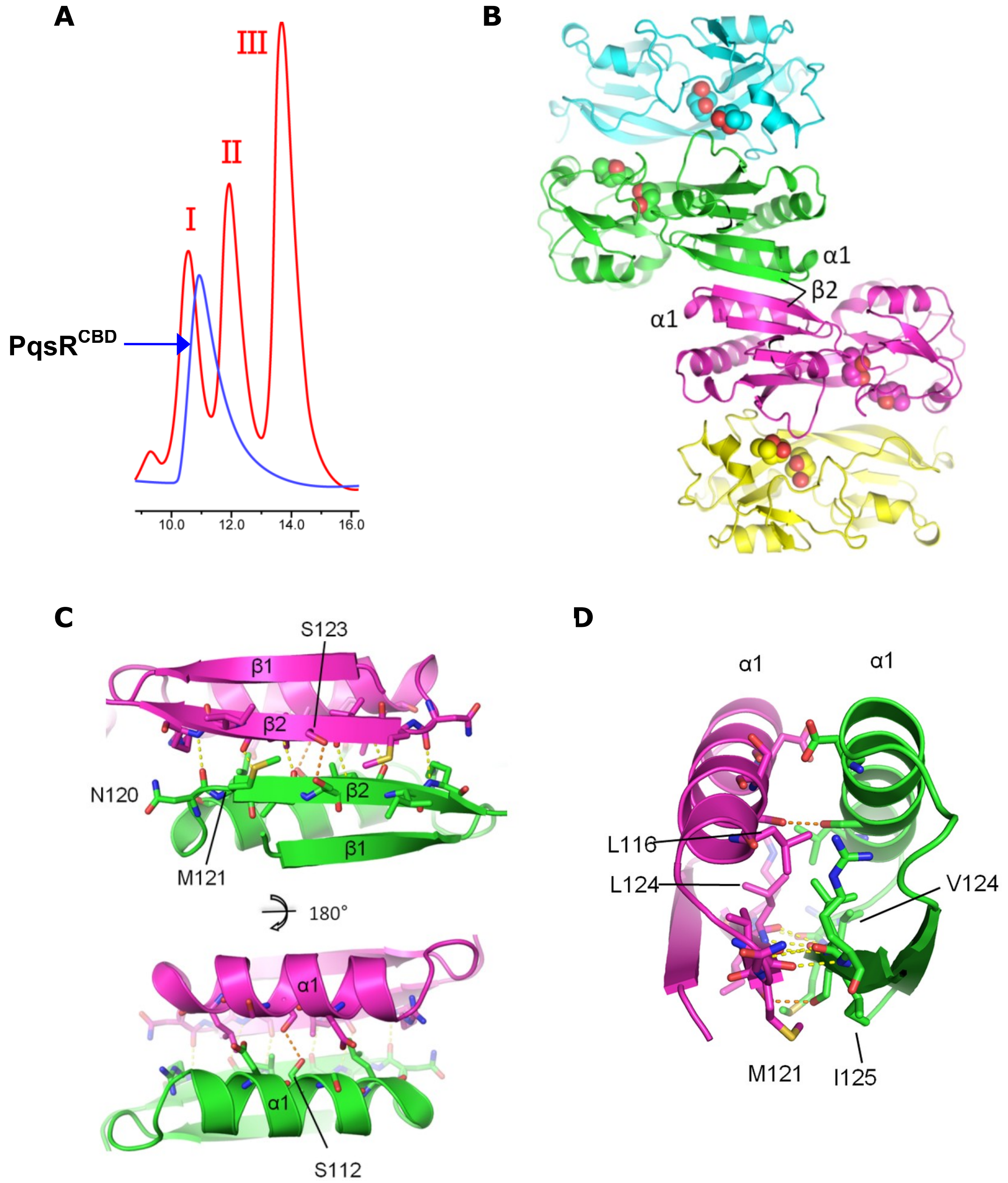

Figure S1. (**A**) Gel filtration chromatogram of PqsR^{CBD} (residues 94-332). Absorbance elution profiles are shown PqsR^{CBD} in blue overlapped with the elution profiles of protein standards (red) using a Superdex 75 10/300 analytical column. The standards include I - ovalbumin (43 kDa), II - carbonic anhydrase (29 kDa) and III - ribonuclease A (13.7 kDa). (**B**) The PqsR^{CBD} tetrameric arrangement observed in the crystal structure shown as topology with MPD shown as spheres. (**C**) A close up view of the PqsR^{CBD} tetramer interface shown as topology. Two views are shown related by a 180 degree rotation with hydrogen bonding interactions highlighted as dotted lines (orange) and key residues shown in stick. (**D**) End view of the interface shown in (**C**).
