# Supplementary material for: Structural Basis for Native Agonist and Synthetic Inhibitor Recognition by the Pseudomonas aeruginosa Quorum Sensing Regulator PqsR (MvfR)
Source: PLoS Pathog. 2013 Jul 25;9(7):e1003508. doi: 10.1371/journal.ppat.1003508 (PMC3723537; doi:10.1371/journal.ppat.1003508)
Supplement: Figure S2 — Electron density maps are shown for bound ligands displayed within a 2 Å radius. (A) Left panel shows the final refined 2.7 Å electron density map (blue) with SigmaA m2Fo-DFc coefficients and phases c of the PqsR-MPD structure contoured at 1.0 r.m.s (REFMAC, CCP4). The modelled MPD molecules are shown as stick and the figure was generated using PyMOL. The middle panel shows the same calculation with the atoms of the MPD excluded (simple omit map). The far right panel is calculated in the absence of any atoms from the ligand with the remaining coordinates utilised in a simulated annealing (SA) combined omit map protocol. The map was displayed in COOT used to generate the figure and contoured at 1.2 r.m.s. (B) Left panel shows the final refined 3.1 Å electron density map (blue) with SigmaA m2Fo-DFc coefficients and phases c of the PqsR-NHQ structure contoured at 1.0 r.m.s (REFMAC). The modelled NHQ molecule is shown as stick and the figure was generated using PyMOL. The middle panel shows the same calculation with the atoms of the NHQ excluded (omit map). The far right panel is the phenix SA omit calculation described in (A) with NHQ atoms omitted and contoured at 1.2 r.m.s. (C) Left panel shows the final refined 3.1 Å electron density map (blue) with SigmaA m2Fo-DFc co-efficients and phases c of the PqsR-3NH2-7Cl-C9QZN (QZN) structure contoured at 1.0 r.m.s (REFMAC). The modelled QZN molecule is shown as stick and the figure was generated using PyMOL. The right panel shows the phenix SA omit calculation as in (A) with the atoms of the QZN excluded. (PDF) [file ppat.1003508.s002.pdf]

Supplementary Figure S2

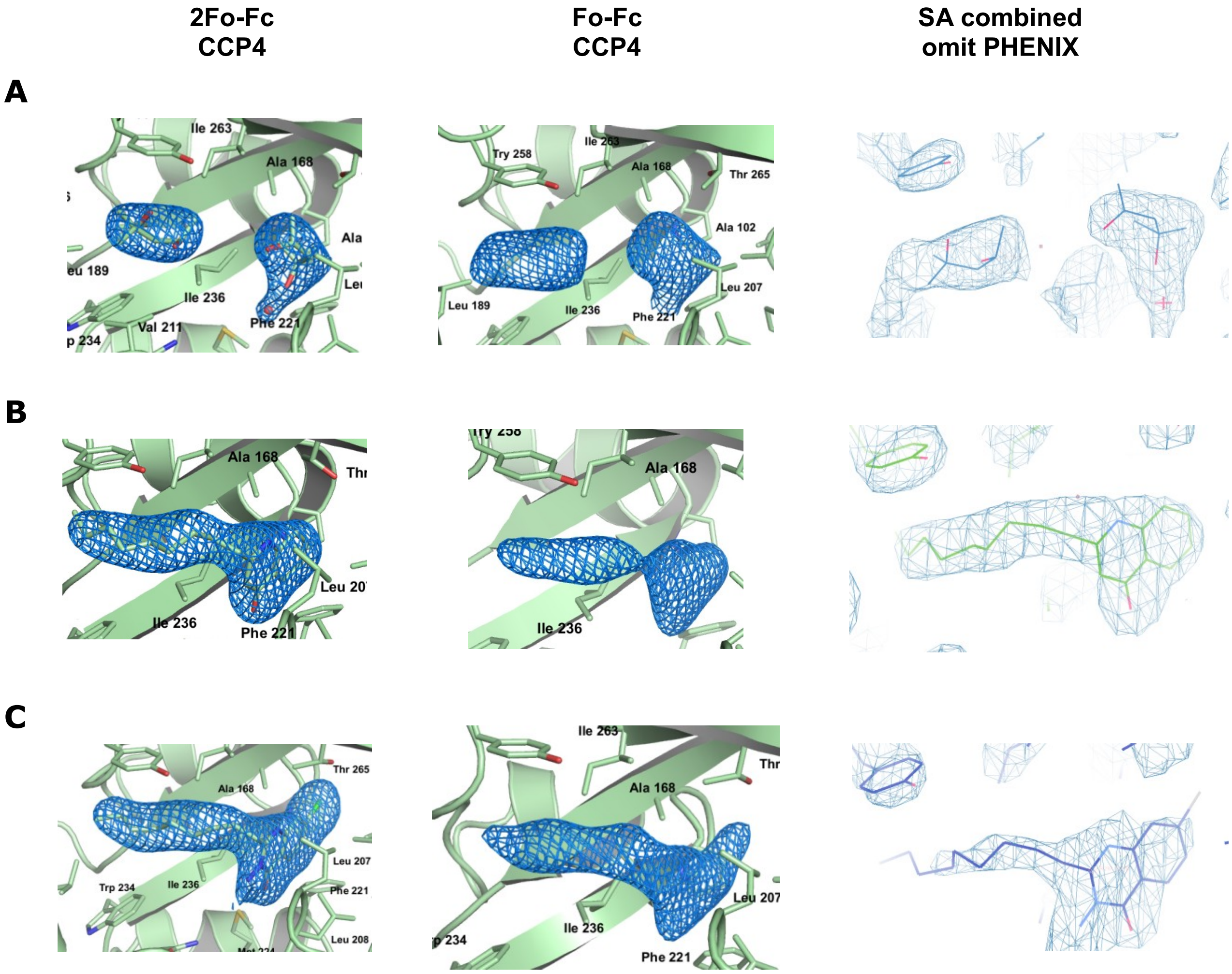

Figure S2. Electron density maps are shown for bound ligands displayed within a 2 Å radius. (**A**) Left panel shows the final refined 2.5 Å electron density map (blue) with SigmaA 2mFo-DFc co-efficients and phases Φc of the PqsR-MPD structure contoured at 1.0 r.m.s (REFMAC, CCP4). The modelled MPD molecules are shown as stick and the figure was generated using PYMOL. The middle panel shows the same calculation with the atoms of the MPD excluded (simple omit map). The far right panel is calculated in the absence of any atoms from the ligand with the remaining coordinates utilised in a simulated annealing (SA) combined omit map protocol. The map was displayed in COOT used to generate the figure and contoured at 1.2 r.m.s. (**B**) Left panel shows the final refined 2.9 Å electron density map (blue) with SigmaA 2mFo-DFc co-efficients and phases Φc of the PqsR-NHQ structure contoured at 1.0 r.m.s (REFMAC). The modelled NHQ molecule is shown as stick and the figure was generated using PYMOL. The middle panel shows the same calculation with the atoms of the NHQ excluded (omit map). The far right panel is the phenix SA omit calculation described in (A) with NHQ atoms omitted and contored at 1.2 r.m.s. (**C**) Left panel shows the final refined 2.95 Å electron density map (blue) with SigmaA 2mFo-DFc co-efficients and phases Φc of the PqsR 3NH2-7Cl-C9QZN (QZN) structure contoured at 1.0 r.m.s (REFMAC). The modelled QZN molecule is shown as stick and the figure was generated using PYMOL. The right panel shows the phenix SA omit calculation as in (A) with the atoms of the QZN excluded.
