# Supplementary material for: Structural Basis for Native Agonist and Synthetic Inhibitor Recognition by the Pseudomonas aeruginosa Quorum Sensing Regulator PqsR (MvfR)
Source: PLoS Pathog. 2013 Jul 25;9(7):e1003508. doi: 10.1371/journal.ppat.1003508 (PMC3723537; doi:10.1371/journal.ppat.1003508)
Supplement: Figure S3 — Unique dimer organisation of PqsR. Cartoon diagrams of the central dimer organisation for LTTRs PqsR, OxyR (pdb: 1I69), BenM (pdb: 2F78) and TsaR (pdb: 3FXQ). β-strands are colored magenta and α-helices in cyan. β-strands involved in the hinge region are indicated by a blue arrow showing the centrally located hinge regions in PqsR compared to the peripheral location of hinge regions on OxyR, BenM and TsaR. (PDF) [file ppat.1003508.s003.pdf]

**Supplementary Figure S3**

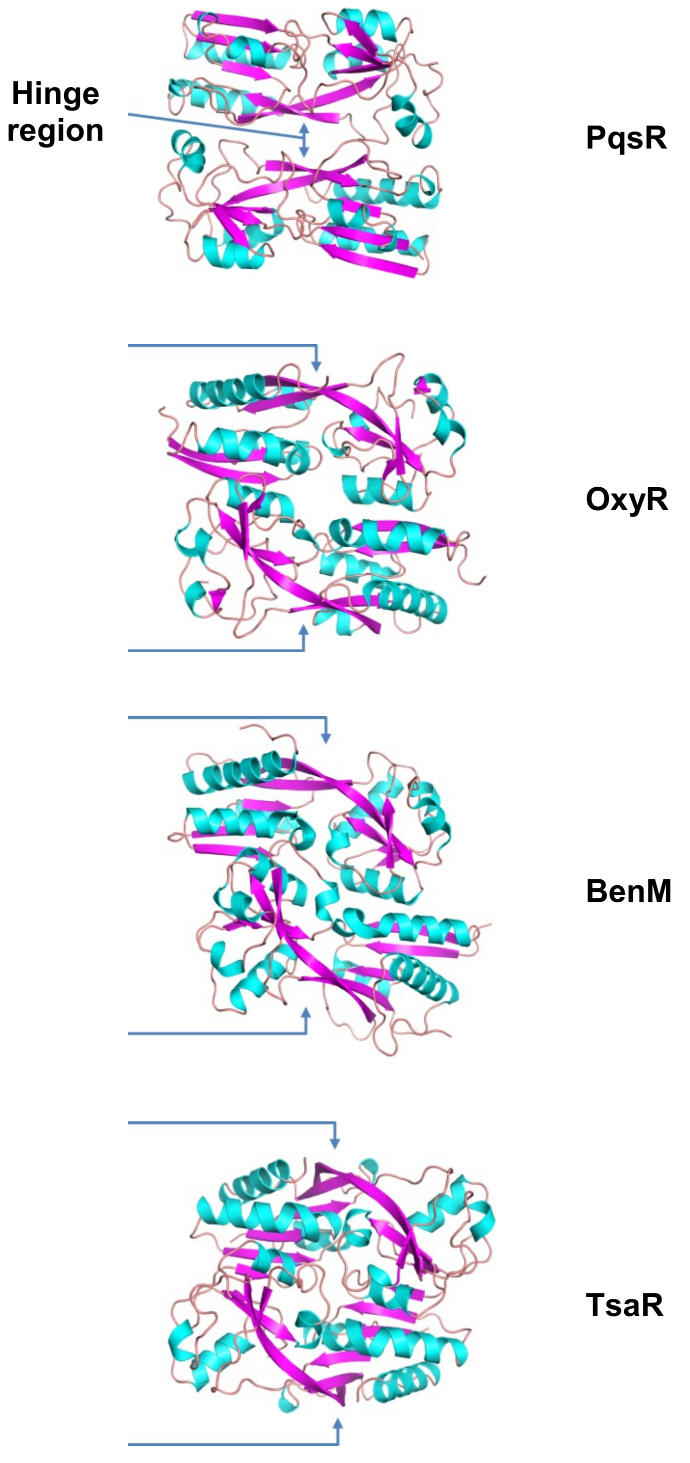


**Figure S3.** Unique dimer organisation of PqsR. Cartoon diagrams of the central dimer organisation for LTTRs PqsR, OxyR (pdb: 1I69), BenM (pdb: 2F78) and TsaR (pdb: 3FXQ). β-strands are colored magenta and α-helices in cyan. β-strands involved in the hinge region are indicated by a blue arrow showing the centrally located hinge regions in PqsR compared to the peripheral location of hinge regions on OxyR, BenM and TsaR.
