# Supplementary material for: Structural Basis for Native Agonist and Synthetic Inhibitor Recognition by the Pseudomonas aeruginosa Quorum Sensing Regulator PqsR (MvfR)
Source: PLoS Pathog. 2013 Jul 25;9(7):e1003508. doi: 10.1371/journal.ppat.1003508 (PMC3723537; doi:10.1371/journal.ppat.1003508)
Supplement: Table S1 — Strains and plasmids used in this study. (PDF) [file ppat.1003508.s004.pdf]

Supplementary Table S1. Strains and plasmids used in this study

Strains/Plasmids	Relevant Characteristics	Source/Reference
E. coli		
Novablue	*gyrA96 relA1 lac* F′ [*proA+B+ lacIqZ*Δ*M15*::Tn*10*] (TetR)	EMD4Biosciences
Rosetta 2 (DE3)	F-*ompT hsdS*B(rB- mB-) *gal dcm* (DE3) pRARE (Cm^R)	EMD4Biosciences
B834 (DE3) pLysS	F-*ompT hsdS*B(rB- mB-) *gal dcm met* (DE3) pLysS	EMD4Biosciences
S17-1λ*pir*	Conjugative strain for suicide plasmids	[1]
SM10λ*pir*	Conjugative strain for suicide plasmids	[1]
P. aeruginosa		
PAO1	wild-type *P. aeruginosa* strain, parent of the strains below	Laboratory collection
PA01 CTX::*pqsA'-lux*	PAO1 with a miniCTX::*pqsA'-luxCDABE* fusion integrated in the chromosomal CTX attachment site	[2]
PAO1 Δ*pqsA*	*pqsA* in-frame deletion mutant; AQ-negative	[2]
PAO1 Δ*pqsA* CTX::*pqsA'-lux*	*pqsA* in-frame deletion mutant with a miniCTX::*pqsA'-luxCDABE* fusion integrated in the chromosome	[3]
PAO1 Δ*pqsAH* CTX::*pqsA'-lux*	double Δ*pqsA* Δ*pqsH* in-frame deletion mutant with a miniCTX::*pqsA'-luxCDABE* chromosomal fusion	[2]
PAO1 Δ*pqsR*	Δ*pqsR* in-frame deletion mutant	This study
PAO1 Δ*pqsAHR*	triple Δ*pqsA* Δ*pqsH* Δ*pqsR* in-frame deletion mutant	This study
PAO1 Δ*pqsR* CTX::*pqsA'-lux*	Δ*pqsR* deletion mutant with a miniCTX::*pqsA'-luxCDABE* chromosomal fusion	This Study
PAO1 Δ*pqsAHR* CTX::*pqsA'-lux*	triple Δ*pqsA* Δ*pqsH* Δ*pqsR* in-frame deletion mutant with a miniCTX::*pqsA'-luxCDABE* chromosomal fusion	This Study
PAO1 *lecA::lux*	*lecA::luxCDABE* chromosomal reporter fusion	[4]
PAO1 CTX::*phzA1'-lux*	PAO1 with a miniCTX::*phzA'-luxCDABE* fusion integrated in the chromosomal CTX attachment site	This study
**Plasmids**		
pBluescript II KS+	Cloning vector; ColE1 replicon; Ap^R	Stratagene
pFLP2	Plasmid providing Flp recombinase; Ap^R	[5]
miniCTX-*lux*	pHKBS1 reporter vector for the insertion of promoter-*luxCDABE* fusions into the CTX attachment site of *P. aeruginosa*; Tc^R	[6]
pCTX::*pqsA'-lux*	Promoter region and part of *pqsA* fused to *luxCDABE* inserted into the miniCTX-1 delivery plasmid. Tc^R	[2]
CTX::*phzA1-lux*	Promoter region and part of *phzA1* fused to *luxCDABE* inserted into the miniCTX-1 delivery plasmid. Tc^R	This Study
pET28a	Expression vector for *E. coli*, Km^R	Novagen
pPqsR-C$^{94-332}$	pET28a containing the PqsR CBD region, residues 94-332; Km^R	This study
pPqsR-C$^{94-294}$	pET28a containing the PqsR CBD region, residues 94-294; Km^R	This study
pBS*pqsR*Up	pBluescript II KS+ carrying the upstream region of *pqsR*; Ap^R	This study
pBS*pqsR*Down	pBluescript-II KS+ carrying the downstream region of *pqsR*; Ap^R	This study
pDM4Δ*pqsR*	pDM4 derivative for the in-frame deletion of *pqsR*; Cm^R	This study
pME6032	pVS1-p15A shuttle expression (IPTG-inducible) vector, Tc^R	[7]
pPqsR	pME6032 derivative expressing native *pqsR*; Tc^R	This Study
pPqsR-6His	pME6032 derivative expressing *pqsR*-6His; Tc^R	This Study
pPqsR-6His-I149A	derivative of pPqsR-6His with I149A substitution; Tc^R	This Study
pPqsR-6His-I149E	derivative of pPqsR-6His with I149E substitution; Tc^R	This Study
pPqsR-6His-A168F	derivative of pPqsR-6His with A168F substitution; Tc^R	This Study
pPqsR-6His-I186A	derivative of pPqsR-6His with I186A substitution; Tc^R	This Study
pPqsR-6His-Q194E	derivative of pPqsR-6His with Q194E substitution; Tc^R	This Study
pPqsR-6His-L207A	derivative of pPqsR-6His with L207A substitution; Tc^R	This Study
pPqsR-6His-L207E	derivative of pPqsR-6His with L207E substitution; Tc^R	This Study
pPqsR-6His-F221A	derivative of pPqsR-6His with F221A substitution; Tc^R	This Study
pPqsR-6His-F221E	derivative of pPqsR-6His with F221E substitution; Tc^R	This Study
pPqsR-6His-F221Y	derivative of pPqsR-6His with F221Y substitution; Tc^R	This Study
pPqsR-6His-I236F	derivative of pPqsR-6His with I236F substitution; Tc^R	This Study
pPqsR-6His-Y258A	derivative of pPqsR-6His with Y258A substitution; Tc^R	This Study
pPqsR-6His-I263W	derivative of pPqsR-6His with I263W substitution; Tc^R	This Study

References for Table S1

1. Simon R, Priefer U, Puhler A (1983) A broad host range mobilization system for *in vivo* genetic engineering, transposon mutagenesis in Gram-negative bacteria. Bio-Technol 1: 784-791.
2. Fletcher MP, Diggle SP, Crusz SA, Chhabra SR, Camara M, et al. (2007) A dual biosensor for 2-alkyl-4-quinolone quorum-sensing signal molecules. Environ Microbiol 9: 2683-2693.
3. Diggle SP, Matthijs S, Wright VJ, Fletcher MP, Chhabra SR, et al. (2007) The *Pseudomonas aeruginosa* 4-quinolone signal molecules HHQ and PQS play multifunctional roles in quorum sensing and iron entrapment. Chem Biol 14: 87-96.
4. Winzer K, Falconer C, Garber NC, Diggle SP, Camara M, et al. (2000) The *Pseudomonas aeruginosa* lectins PA-IL and PA-IIL are controlled by quorum sensing and by RpoS. J Bacteriol 182: 6401-6411.
5. Hoang TT, Karkhoff-Schweizer RR, Kutchma AJ, Schweizer HP (1998) A broad-host-range Flp-FRT recombination system for site-specific excision of chromosomally-located DNA sequences: application for isolation of unmarked *Pseudomonas aeruginosa* mutants. Gene 212: 77-86.
6. Becher A, Schweizer HP (2000) Integration-proficient *Pseudomonas aeruginosa* vectors for isolation of single-copy chromosomal *lacZ* and *lux* gene fusions. Biotechniques 29: 948-952.
7. Heeb S, Blumer C, Haas D (2002) Regulatory RNA as mediator in GacA/RsmA-dependent global control of exoproduct formation in *Pseudomonas fluorescens* CHA0. J Bacteriol 184: 1046-1056.
