# Supplementary material for: Structural Basis for Native Agonist and Synthetic Inhibitor Recognition by the Pseudomonas aeruginosa Quorum Sensing Regulator PqsR (MvfR)
Source: PLoS Pathog. 2013 Jul 25;9(7):e1003508. doi: 10.1371/journal.ppat.1003508 (PMC3723537; doi:10.1371/journal.ppat.1003508)
Supplement: Table S2 — Oligonucleotides used in this study. (PDF) [file ppat.1003508.s005.pdf]

Supplementary Table S2. Oligonucleotides used in this study

Primer	Sequence[a]	Restriction site[b]
*pqsR*C94F	5'-TGCTAGCAATCTCCGGGTGCTGCT-3'	NheI
*pqsR*C332R	5'-GATAAGCTTTCACTCTGGTGCGGCGC-3'	HindIII
*pqsR*C309R	5'-CGATAAGCTTTCAGCTCGGTTGCCAGGC-3'	HindIII
*pqs*RC296R	5'-CGATAAGCTTTCAGCCGAGTTCGCGCAG-3'	HindIII
FW*pqsR*Up	5'-ATAAAGCTTTCTTAGAACCGTTCCTGG-3'	HindIII
RV*pqsR*Up	5'-ATAGGATCCAGGTTATGAATAGGCATC-3'	BamHI
FW*pqsR*Down	5'-ATAGGATCCAGAGTAGAGCGTTCTCCA-3'	BamHI
RV*pqsR*Down	5'-ATATCTAGAAGTTCTGCCTGCTCGGCG-3'	XbaI
FW*pqsR*	5'-TATGAATTCATGCCTATTCATAACCTGAATCA-3'	EcoRI
RV*pqsR*	5'-TATGAGCTCCTACTCTGGTGCGGCGCG-3'	SacI
RV*pqsR-6H*	5'-TATGAGCTCCTAGTGATGGTGATGGTGATGCTCTGGTGCGGCGCGCTG-3'	SacI
FW*pqsR-6H* I149A	5'-ATCGCCATCACC**GCC**GACGAGGAACTG-3'	-
RV*pqsR-6H* I149A	5'-CAGTTCCTCGTC**GGC**GGTGATGGCGAT-3'	-
FW*pqsR-6H* I149E	5'-ATCGCCATCACC**GAG**GACGAGGAACTG-3'	-
RV*pqsR-6H* I149E	5'-CAGTTCCTCGTC**CTC**GGTGATGGCGAT-3'	-
FW*pqsR-6H* A168F	5'-GGCTACACCAAG**TTC**TTCGTCGTCGCC-3'	-
RV*pqsR-6H* A168F	5'-GGCGACGACGAA**GAA**CTTGGTGTAGCC-3'	-
FW*pqsR-6H* I186A	5'-CCCTGCACAGC**GCC**GCGAGCCTGGC-3'	-
RV*pqsR-6H* I186A	5'-GCCAGGCTCGC**GGC**CTGTGCAGGG-3'	-
FW*pqsR-6H* Q194E	5'-GCCAATTACCGG**GAG**ATCAGCCTCGG-3'	-
RV*pqsR-6H* Q194E	5'-CCGAGGCTGAT**CTC**CCGGTAATTGGC-3'	-
FW*pqsR-6H* L207A	5'-CAGCATTCGAAC**GCC**CTGCGGCCGGTC-3'	-
RV*pqsR-6H* L207A	5'-GACCGGCCGCAG**GGC**GTTCGAATGCTG-3'	-
FW*pqsR-6H* L207E	5'-CAGCATTCGAAC**GAG**CTGCGGCCGGTC-3'	-
RV*pqsR-6H* L207E	5'-ACCGGCCGCAG**CTC**GTTCGAATGCTG-3'	-
FW*pqsR-6H* F221A	5'-TTCGTGGAAAAC**GCC**GACGACATGCTG-3'	-
RV*pqsR-6H* F221A	5'-CAGCATGTCGTC**GGC**GTTTTCCACGAA-3'	-
FW*pqsR-6H* F221E	5'-TTCGTGGAAAAC**GAG**GACGACATGCTG-3'	-
RV*pqsR-6H* F221E	5'-CAGCATGTCGTC**CTC**GTTTTCCACGAA-3'	-
FW*pqsR-6H* F221Y	5'-TTCGTGGAAAAC**TAC**GACGACATGCTG-3'	-
RV*pqsR-6H* F221Y	5'-CAGCATGTCGTC**GTA**GTTTTCCACGAA-3'	-
FW*pqsR-6H* I236F	5'-TCGGATGGGGC**TTC**GCGCCGCATTAT-3'	-
RV*pqsR-6H* I236F	5'-ATAATGCGGCGC**GAA**GCCCCATCCGA-3'	-
FW*pqsR-6H* Y258A	5'-CTCAGCGAACTC**GCC**GAACCGGGCGG-3'	-
RV*pqsR-6H* Y258A	5'-CCGCCCGGTTC**GGC**GAGTTCGCTGAG-3'	-
FW*pqsR-6H* I263W	5'-GAACCGGGCGGC**TGG**GACACCAAGGTG-3'	-
RV*pqsR-6H* I263W	5'-CACCTTGGTGTC**CCA**GCCGCCCGGTTC-3'	-

[a] added restriction sites are underlined; substituted codons are in bold
[b] –, no restriction site added
