# Supplementary material for: Structural Basis for Native Agonist and Synthetic Inhibitor Recognition by the Pseudomonas aeruginosa Quorum Sensing Regulator PqsR (MvfR)
Source: PLoS Pathog. 2013 Jul 25;9(7):e1003508. doi: 10.1371/journal.ppat.1003508 (PMC3723537; doi:10.1371/journal.ppat.1003508)
Supplement: Text S1 — Supplemental Materials and Methods and 1H NMR spectra. (PDF) [file ppat.1003508.s006.pdf]

Supplementary Text S1: Synthesis and characterisation of AQs and QZNs

Supplementary Materials and Methods **1-32**
References.. **32**
^{1}H NMR Spectra of selected compounds... **33-45**

PqsR^{CBD}-MPD Structure Determination

Heavy atom reagents from the Hampton kit were utilised in the range of 0.5 μM to 1 mM soaking crystals for 24 to 48 hours. Data was collected on a 1 μM solution of K_2PtCl_4 soaked into PqsR$^{94\text{-}332}$ crystals for 4 hours and data were collected close to the peak wavelength of the Pt atom. The program SOLVE[1] was used to determine a single heavy atom site and SIR and SAD phases combined with solvent flattening resulted in a map with a clear boundary and protein features but was of insufficient quality to build a complete model. The PqsR$^{94\text{-}332}$ sequence has 2 Met residues which were replaced by selenomethionine (SeMet). The SeMet PqsR$^{94\text{-}332}$ crystallized in the same condition as the native and data were collected to 3.5Å. Peak wavelength SAD data enabled location of 2 Se sites using SOLVE. Native, K_2PtCl_4 and SeMet datasets were scaled together (FHSCAL) for a SIRAS calculation in SOLVE resulting in an overall Z score of 17.1 and a mean figure-of-merit (FOM) of 0.46. The phases were improved by solvent flattening using DM with 70% solvent content and the 3.5Å resolution phases were extended to the limit of the native data (2.7Å). Initial model building was performed using BUCCANEER and was completed by manually using COOT[2]. The structure was refined using REFMAC to give a final R_{cryst} of 0.25 and R_{free} of 0.28.

AQ and QZN Synthesis

The starting materials were purchased from Aldrich Chemical Co. and Alfa Aesar. The solvents used were of HPLC grade. Melting points were measured using a Gallenkamp or a Griffin melting point apparatus and are reported uncorrected. ^{1}H NMR spectra were recorded as $CDCl_3$ or DMSO-d_6 sample solutions on Bruker Avance-400 instruments operating at 400 MHz. Chemical shifts were referenced to an internal standard $SiMe_4$. ES-MS spectra were recorded using Waters Micromass LCT spectrometer. TLC was performed using Merck silica gel 60 GF_{254} pre-coated (0.2 mm) aluminum plates. Preparative layer chromatography (PLC) was performed using Merck silica gel 60 GF_{254} pre-coated (1.0 mm) glass plates. For column purification a Biotage Flashmaster Personal system was used with Biotage Isolute Flash SiII cartridges (10 g, 20 g and 70 g). All products during chromatography were visualized using either UV light (λ = 254 nm) or by staining with dilute potassium permanganate solutions.

2-Alkyl-3-hydroxy-4(1*H*)-quinolones (PQS 1, C9-PQS 4 and **7-Cl-PQS 5)**

PQS **1** and C9-PQS **4** have been previously prepared by Pesci *et al.*[3] from the corresponding HHQ **2** and NHQ **3** by their conversion to 3-formyl derivatives by Duff reaction followed by Baeyer-Villiger oxidation to the desired 3-hydroxy products. As this procedure had a limited scope and invariably gave poor yields[4], we have developed an alternative two step synthesis based on a report by Hradil *et al*.[5]. The procedure is outlined in **Scheme 2** and involves the esterification of appropriate anthranilic acids with α-chloroketones followed by thermal dehydrative cyclisation to form 2-alkylbenzo[e][1,4]oxazepin-5(1*H*)-one intermediates which undergo concurrent ring contraction to deliver PQS and its variants in good yields[6]. Recently Hodgkinson *et al*.[7] have adapted this approach to synthesise PQS and its analogues in improved yields by incorporating microwave and flow methods. The synthesis of the key component**, α-chloroketone** is summarised in **Scheme 1**[8].

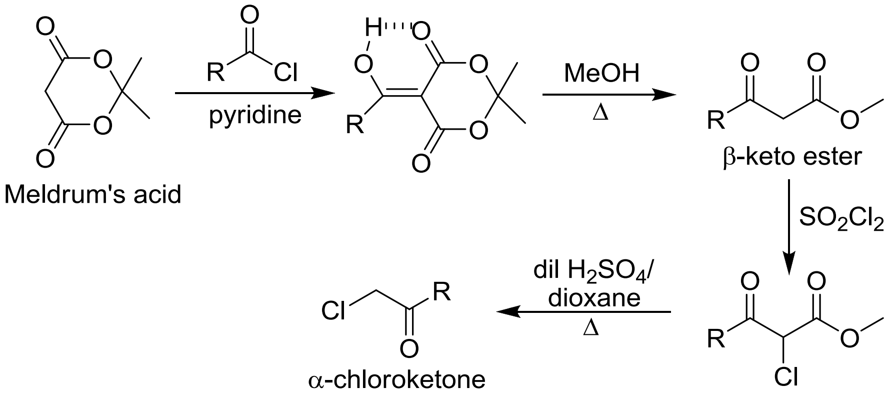

Scheme 1: Synthesis of α-chloroketones

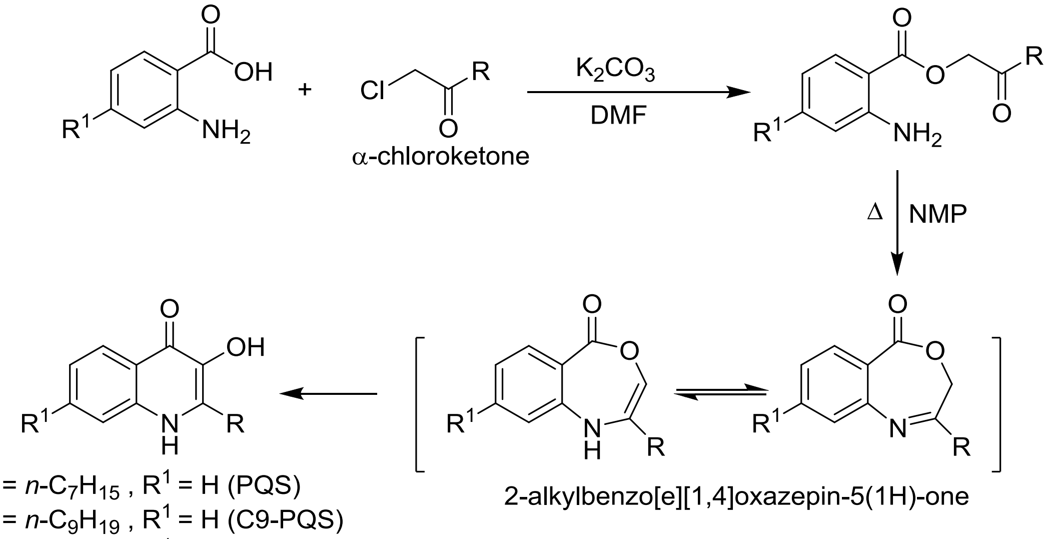

1 R = n-C_7H_{15} , R^1 = H (PQS)
4 R = n-C_9H_{19} , R^1 = H (C9-PQS)
5 R = n-C_7H_{15} , R^1 = Cl (7-Cl-PQS)

Scheme 2: Synthesis of PQS and its analogues

2-Alkyl-4(1*H*)-quinolones (HHQ 2 and **NHQ 3)** were synthesised by the method of Somanathan and Smith[9] as outlined **in Scheme 3.** The key components**, β-Keto esters** were prepared from Meldrum's acid as shown above in **Scheme 1**[10].

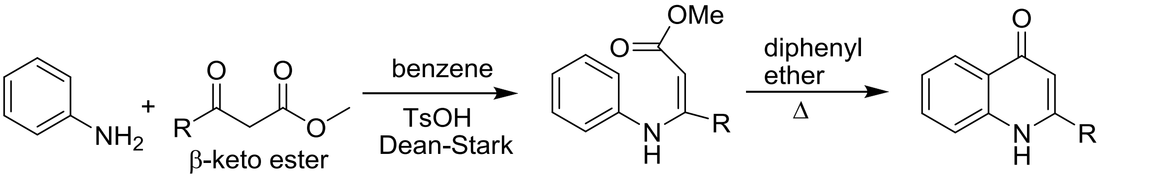

2 R = n-C_7H_{15} (HHQ)
3 R = n-C_9H_{19} (NHQ)

Scheme 3: Synthesis of HHQ and its analogue NHQ

2-*n*-Heptyl-3-hydroxy-4(1*H*)-quinolone (PQS)

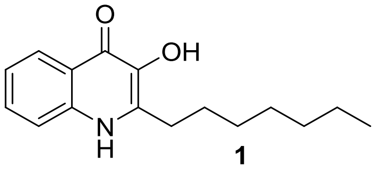

1

Methyl 3-oxodecanoate

Pyridine (16.2 ml, 0.2 mol) was added to a solution of Meldrum's acid (14.4 g, 0.1 mol) in dichloromethane (100 ml) at 0-5 ^{o}C followed by the addition of octanoyl chloride (18.8 ml, 0.11 mol) over 20 min. The resulting mixture was stirred at 0-5 ^{o}C for 1 h followed by a further 1 h stir at room temperature. The reaction mixture was washed with 1 M HCl, water and brine. The organic layer was dried over magnesium sulphate and concentrated under reduced pressure to give 5-octanoyl Meldrum's acid as a brown oil (25.6 g, 95%).

5-Octanoyl Meldrums Acid (25.6 g) and methanol (150 ml) were heated at reflux for 2 h. The solvent was removed under vacuum and the resulting crude brown oil was purified by high vacuum distillation to give *methyl 3-oxo-decanoate* as a clear oil (14.1 g, 74.2%).

^{1}H NMR ($CDCl_3$) δ 0.90 (3H, t, Me), 1.30 (8H, m, $(CH_2)_4$Me), 1.61 (2H, m, $CH_2(CH_2)_4$Me), 2.55 (2H, t, $CH_2(CH_2)_5$Me), 3.47 (2H, s, COCH_2CO), 3.76 (3H, s, OMe)

Methyl 2-chloro-3-oxodecanoate

Sulphuryl chloride (2.84 g, 0.021 mol) was added over 5 min to a solution of methyl 3-oxo-decanoate (4.0 g, 0.02 mol) and the mixture was stirred at room temperature for 2 h. Water (25 ml) was added carefully with cooling and stirred at room temperature for 15 min. The lower aqueous layer was removed and the organic layer was washed with brine (15 ml), dried over magnesium sulphate and concentrated under reduced pressure to give *methyl 2-chloro-3-oxodecanoate* as a clear oil (4.04 g, 86.1%).

^{1}H NMR ($CDCl_3$) δ 0.91 (3H, t, Me), 1.31 (8H, m, $(CH_2)_4$Me), 1.64 (2H, m, $CH_2(CH_2)_4$Me), 2.73 (2H, t, $CH_2(CH_2)_5$Me), 3.86 (3H, s, OMe), 4.82 (1H, s, CO*CH*ClCO)

1-Chloro-2-nonanone

Methyl 2-chloro-3-oxo-decanoate (2.0 g, 8.5 mmol), sulphuric acid (4.0 g), water (6 ml) and dioxane (2 ml) were heated at reflux for a total of 48 h. The mixture was cooled, diluted with water (40 ml) and extracted with dichloromethane (3 x 10 ml). The organic layer was washed with water (10 ml) and brine (10 ml), dried over magnesium sulphate and concentrated under reduced pressure to give *1-chloro-2-nonanone* as a dark brown oil. (Yield = 1.0 g, 66.7%)

^{1}H NMR ($CDCl_3$) δ 0.90 (3H, t, Me), 1.31 (8H, m, $(CH_2)_4$Me), 1.64 (2H, m, $CH_2(CH_2)_4$Me), 2.61 (2H, t, $CH_2(CH_2)_5$Me), 4.10 (2H, s, COCH_2Cl)

2-Oxononyl anthranilate

Potassium carbonate (0.674 g, 4.9 mmol) was added to a mixture of anthranilic acid (0.933 g, 6.8 mmol) in DMF (10 ml) and heated at 90 ^{o}C for 1 h. The mixture was cooled to 50 ^{o}C and 1-chloro-2-nonanone (1.0 g, 5.7mmol) was added. The mixture was stirred at 50 ^{o}C for

30 min, cooled to room temperature and poured into ice/water (100 ml). The resulting slurry was stirred at 0-10 °C for 1h before being filtered and washed with water (20 ml) to give *2-oxononyl anthranilate* as a pale brown solid (1.25 g, 79.2%).

^{1}H NMR (DMSO-d$_6$) δ 0.86 (3H, t, Me), 1.25 (8H, m, *(CH$_2$)$_4$*Me), 1.52 (2H, m, *CH$_2$*(CH$_2$)$_4$Me), 2.47 (2H, m, *CH$_2$*(CH$_2$)$_5$Me), 4.94 (2H, s, COCH$_2$CO), 6.55 (1H, dt, 3H), 6.64 (1H, b, NH), 6.79, (1H, dd, 4H), 7.28 (1H, dt, 5H), 7.76, (1H, dd, 6H)

2-*n*-Heptyl-3-hydroxy-4(1*H*)-quinolone (PQS)

2-Oxononyl anthranilate (1.0 g, 3.6 mmol) was heated at reflux in *N*-methyl-2-pyrrolidone (NMP) (3.0 ml) for 1 h 15 min and then cooled to 50 °C. Ethyl acetate (12 ml) was added and on cooling to room temperature a cream solid was precipitated. The precipitate was stirred at 0 to 5 °C for 30 min , collected by filtration and washed with water (6.0 ml), cold ethanol (2.0 ml), and cold ethyl acetate (2.0 ml) to obtain **PQS** as a cream/white solid (0.547 g, 58.5%).

mp 192-194 °C (Lit. 190-192 °C; [7])

^{1}H NMR (DMSO-d$_6$) δ 0.85 (3H, t, J = 7 Hz, Me), 1.2-1.4 (8H, m, *(CH$_2$)$_4$*Me), 1.67 (2H, m, *CH$_2$*(CH$_2$)$_4$Me), 2.71 (2H, t, J = 8 Hz, *CH$_2$*(CH$_2$)$_5$Me), 7.22(1H, 8-H), 7.54 (2H, 6-H & 7-H), 8.00 (1H, b, NH) 8.09 (1H, 5-H), 11.40 (1H, s, OH)

ES-MS *m/z* 260.1658 [M+H]$^+$, $C_{16}H_{22}NO_2^+$ requires 260.1651

2-Hepty-4(1*H*)-quinolone (HHQ)

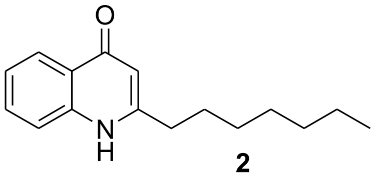

2

Methyl 3-oxodecanoate (as described for **1**) (20.0 g, 0.1 mol), aniline (9.765 g, (0.105 mol), tosic acid (0.115 g), and dry benzene (200 ml) were heated at reflux overnight using a Dean and Stark arm to remove any water produced. The solvent was removed under reduced pressure to give *Methyl 2-anilinodecanoate as* an orange oil in quantitative yield which was used immediately in the next step.

Methyl-2-anilinodecanoate (27.5 g, 0.1 mol) was added dropwise over 30 min to refluxing diphenyl ether (b.p. 260 °C) (27.0 ml). The mixture was heated at reflux for a further period of 30 min before cooling to room temperature to give a pale brown solution which was poured into petroleum ether, b.p. 40-60 °C (4.5 litres). An insoluble oil precipitated which slowly solidified with stirring over 24 h .The majority of the solvent was decanted off to leave behind a brown solid which was collected by filtration (3.063 g, 12.6%). The solid was purified by recrystallisation from ethyl acetate to give **2-*n*-hepty-4(1*H*)-quinolone (HHQ)** as a pale brown solid (2.38 g).

mp 140-142 °C (Lit. 138-141 °C; [9])

^{1}H NMR ($CDCl_3$) δ 0.82 (3H, t, J = 7 Hz, Me), 1.2-1.4 (8H, m, *(CH_2)$_4$*Me), 1.74 (2H, m, *CH_2*(CH_2)$_4$Me), 2.74 (2H, t, J = 8 Hz, *CH_2*(CH_2)$_5$Me), 6.30 (1H, 3-H), 7.36(1H, 8-H), 7.63 (1H, 6-H), 7.88 (1H, 7-H), 8.39 (1H, 5-H), 12.80 (1H, s, NH)
ES-MS *m/z* 244.1699 $[M+H]^+$, $C_{16}H_{22}NO^+$ requires 244.1701)

2-*n*-Nonyl-4(1*H*)-quinolone (NHQ)

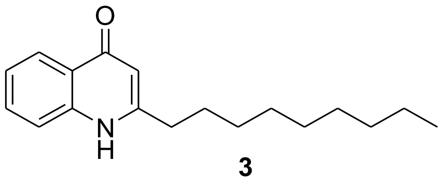

3

NHQ was prepared using the methods described for **HHQ**
mp 129-131 °C (Lit. 129-132 °C; [9])
^{1}H NMR ($CDCl_3$) δ 0.86 (3H, t, J = 7 Hz, Me), 1.2-1.4 (12H, m, *(CH_2)$_6$*Me), 1.73 (2H, m, *CH_2*(CH_2)$_6$Me), 2.70 (2H, t, J = 8 Hz, *CH_2*(CH_2)$_7$Me), 6.25 (1H, 3-H), 7.34(1H, 8-H), 7.60 (1H, 6-H), 7.95 (1H, 7-H), 8.38 (1H, 5-H), 11.90 (1H, s, NH)
ES-MS *m/z* 272.2033 $[M+H]^+$, $C_{18}H_{26}NO^+$ requires 272.2014.

3-Hydroxy-2-*n*-nonyl-4(1*H*)-quinolone (C9-PQS)

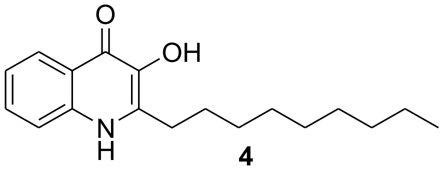

4

C9-PQS was prepared using the methods described for **PQS.**
mp 184-185 °C (Lit 184-187 °C; [7])
^{1}H NMR (DMSO-d_6) δ 0.84 (3H, t, J = 7 Hz, Me), 1.2-1.4 (12H, m, *(CH_2)$_6$*Me), 1.66 (2H, m, *CH_2*(CH_2)$_7$Me), 2.73 (2H, t, J = 8 Hz, *CH_2*(CH_2)$_7$Me), 7.22(1H, 8-H), 7.53 (2H, 6-H & 7-H), 8.00 (1H, b, NH), 8.12 (1H, 5-H), 11.44 (1H, s, OH)
ES-MS *m/z* 288.1965 $[M+H]^+$, $C_{18}H_{26}NO_2^+$ requires 288.1964.

7-Chloro-2-*n*-heptyl-3-hydroxy-4(1*H*)-quinolone (7-Cl-PQS)

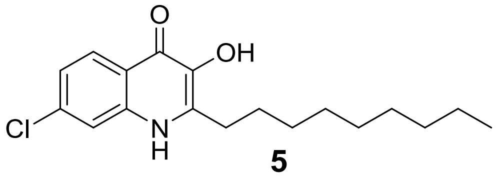

5

7-Cl-PQS was prepared from 4-chloroanthranilic acid using the methods described for **PQS.**
Yield = 0.481g (51.1 %) Off white solid
mp = 257-261 °C (Lit 264-269 °C;)
^{1}H NMR (DMSO-d_6) δ 0.86 (3H, t, J = 7 Hz, Me), 1.2-1.4 (8H, m, *(CH_2)$_4$*Me), 1.66 (2H, m, *CH_2*(CH_2)$_4$Me), 2.72 (2H, t, J = 8 Hz, *CH_2*(CH_2)$_5$Me), 7.23(1H, 6-H), 7.56 (2H, 8-H), 8.10 (1H, 5-H) 8.23 (1H, b, NH), 11.48 (1H, s, OH)
ES-MS *m/z* 294.1182 $[M+H]^+$, $C_{16}H_{21}ClNO_2^+$ requires 294.1183

2-Alkyl-4(3*H*)-quinazolinones were synthesised by established literature procedures essentially in three steps as outlined in **Scheme 4**[6,11,12]. The acylation of appropriate anthranilic acids with acid chlorides gave ***N*-Alkanoylanthranilic acids** which via dehydrative cyclisation yielded **2- Alkylbenzo[*d*][1,3]oxazin-4-ones**. The latter when aminated in refluxing ethanol or THF delivered the desired **quinazolinones** as crystalline solids after purification.

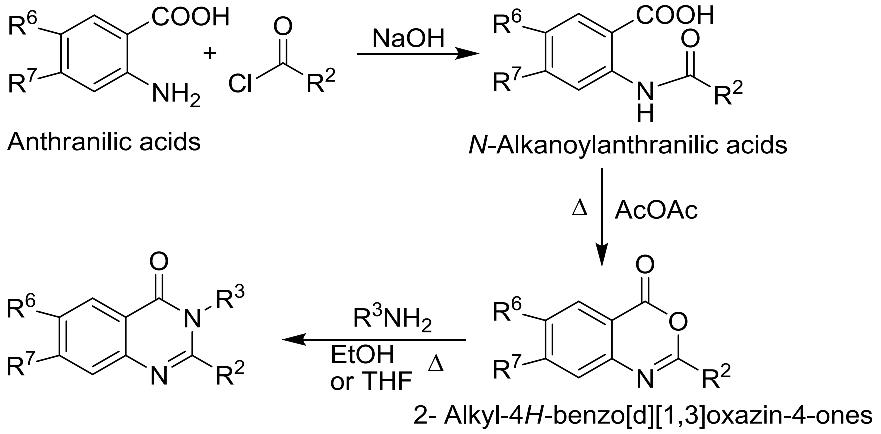

Scheme 4: Synthesis of 2-Alkyl-4(3*H*)-quinazolinones

General Procedure 1 (GP1)

The *N*-Alkanoylanthranilic acids

The alkanoyl chloride (0.0275 mol) and a solution of sodium hydroxide, (0.025 mol) in water (10 ml) were simultaneously added dropwise over 15 min to a solution of anthranilic acid (0.025 mol) and sodium hydroxide (0.025 mol) in distilled water (10 ml) at 0-5 °C. The resulting slurry was stirred at 0-5 °C for 30 min followed by a further 30 min stir at room temperature. The reaction mixture was then acidified to pH 1.0 with concentrated HCl (3.5 ml) and extracted with ethyl acetate (30 ml) which was washed with brine (10 ml), dried with magnesium sulphate and the solvent was removed under reduced pressure. The resulting solid residue was stirred with petroleum ether bp 60-80 °C (15 ml) for 1 h. The product was collected by filtration and washed to give the title acid as a crystalline solid.

General Procedure 2 (GP2)

2-Alkyl-4*H*-benzo[*d*][1,3]oxazin-4-ones

A mixture of *N*-alkanoylanthranilic acid (4.0 g) and acetic anhydride (25 ml) was stirred at reflux for 2 h. After cooling the solvent was removed under high vacuum to give the title compound.

General Procedure 3 (GP3)

2-Alkyl-3-amino-4(3*H*)-quinazolinones

A solution of 2-alkylbenzo[d][1,3]oxazin-4-one (1 mmol) and hydrazine monohydrate (4 mmol) in ethanol (20 ml) was stirred at reflux overnight. The solvent was removed under reduced pressure to give a solid which was either purified by recrystallisation or Flash chromatography (ethyl acetate in hexane) to deliver the title compounds as solids.

General Procedure 4 (GP4)

The Alkanoyl chlorides

A solution of alkanoic acid (0.0261 mol), thionyl chloride (3.26 g=2 ml, 0.0274 mol, 1.05 eqv), DMF (4 drops) in toluene (10 ml) was stirred at room temperature for 15 min. The solution was then stirred at 60 to 65 °C for 1h after which the solvent was removed under reduced pressure and the resulting clear oil was used directly. (Yield = quantitative)

2-*n*-Heptyl-4(3*H*)-quinazolinone (C7-QZN)

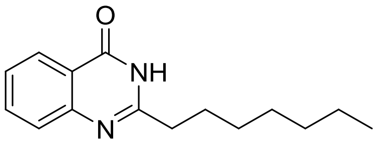

6

N-Octanoylanthranilic acid was prepared in 82% yield as a pale brown solid by the GP1 using anthranilic acid and octanoyl chloride and converted to 2-*n*-heptyl-4*H*-benzo[*d*][1,3]oxazin-4-one as an oily brown solid in quantitative yield by GP2.

2-*n*-Heptyl-4*H*-benzo[*d*][1,3]-oxazin-4-one (0.245 g, 1 mmol) and ammonium acetate (0.154 g, 2 mmol) were heated at 170 °C for 2 h. The residue was cooled to room temperature and taken up in ethyl acetate (40 ml) and water (25 ml) after 30 min of stirring the mixture fully dissolved. The lower aqueous was removed and the organic layer was washed with saturated sodium bicarbonate, 1 M HCl, and brine. The organic layer was dried over magnesium sulphate and concentrated to dryness to give a cream solid which was purified by recrystallisation from ethyl acetate/hexane to deliver the title compound as an off white solid (0.102 g, 42%).

mp 135-136 °C (Lit. mp 143-145 °C; [12])

^{1}H NMR ($CDCl_3$) δ 0.90 (3H, t, J = 7 Hz, Me), 1.25-1.5 (8H, m, *$(CH_2)_4$*Me), 1.88 (2H, m, *CH_2*$(CH_2)_5$Me), 2.78 (2H, t, J = 8 Hz, *CH_2*$(CH_2)_6$Me), 7.49 (1H, 8-H), 7.73 (1H, 6-H), 7.79 (1H, 7-H), 8.30 (1H, 5-H), 10.86 (1H, s, NH)

ES-MS *m/z* 245.1629 $[M+H]^+$, $C_{15}H_{21}N_2O^+$ requires 245.1654.

2-*n*-Nonyl-4(3*H*)-quinazolinone (C9-QZN)

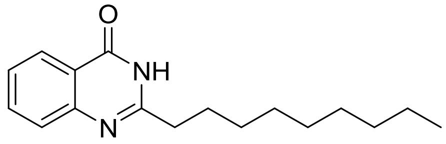

7

N-Decanoylanthranilic acid was prepared as a pale brown solid in 75% yield by GP1 using anthranilic acid and decanoyl chloride and converted to 2-*n*-nonyl-4*H*-benzo[*d*][1,3]oxazin-4-one as a brown oil in quantitative yield by GP2.

2- *n*-Nonyl-4*H*-benzo[d][1,3]-oxazin-4-one (0.273 g, 1 mmol) and ammonium acetate (0.154 g, 2 mmol) were heated at 170 °C for 2 h. The residue was cooled to room temperature and taken up in ethyl acetate (40 ml) and water (25 ml) after 30 min of stirring the mixture was fully dissolved. The aqueous layer was removed and the organic layer was washed with saturated sodium bicarbonate, 1 M HCl, and brine. The organic layer was dried over magnesium sulphate and concentrated to dryness to give a cream solid which was purified by recrystallisation from ethyl acetate to deliver the title compound as a white solid (0.127 g, 47%).

mp 120-121 °C

^{1}H NMR ($CDCl_3$) δ 0.91 (3H, t, J = 7 Hz, Me), 1.25-1.5 (12H, m, *(CH$_2$)$_6$*Me), 1.87 (2H,quintet, J = 8 Hz, *CH$_2$*(CH$_2$)$_6$Me), 2.76 (2H, t, J = 7.7 Hz, *CH$_2$*(CH$_2$)$_7$Me), 7.48 (1H, 8-H), 7.72 (1H, 6-H), 7.79 (1H, 7-H), 8.29 (1H, 5-H), 10.35 (1H, s, NH_2),

ES-MS *m/z* 273.1946 $[M+H]^+$, $C_{17}H_{25}N_2O^+$ requires 273.1967)

7-Fluoro 2-*n*-nonyl -4(3*H*)-quinazolinone (7F-C9-QZN)

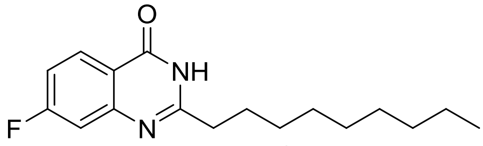

8

N-Decanoyl-4-fluoroanthranilic acid was prepared as a pale brown solid in 58.2% yield by GP1 using 4-fluoroanthranilic acid and decanoyl chloride and converted to 7-fluoro-2-*n*-nonyl-4*H*-benzo[*d*][1,3]oxazin-4-one as a brown oil in quantitative yield by GP2.

7-fluoro-2-*n*-nonyl-4*H*-benzo[*d*][1,3]oxazin-4-one (0.290 g, 1 mmol) and ammonium acetate (0.154 g, 2 mmol) were heated at 170 °C for 2 h. The residue was cooled to room temperature and taken up in ethyl acetate (40 ml) and water (25 ml) after 30 min of stirring the mixture fully dissolved. The lower aqueous was removed and the organic layer was washed with water, saturated sodium bicarbonate and brine. The organic layer was dried over magnesium sulphate and concentrated to dryness to give a cream solid which was purified using Flash chromatography (20% ethyl acetate in hexane) to deliver the title compound as a white solid. (0.065 g, 22.4%)

mp 130-131 °C

^{1}H NMR ($CDCl_3$) δ 0.91 (3H, t, J = 7 Hz, Me), 1.25-1.5 (12H, m, *$(CH_2)_6$*Me), 1.87 (2H, m, *CH_2*$(CH_2)_6$Me), 2.76 (2H, t, J = 8 Hz, *CH_2*$(CH_2)_7$Me), 7.20 (1H, 6-H), 7.37 (1H, 8-H), 8.30 (1H, 5-H), 10.71 (1H, s, NH),
ES-MS *m/z* 291.1866 $[M+H]^+$, $C_{17}H_{24}FN_2O^+$ requires 291.1873)

2-*n*-Heptyl-3-hydroxy-4(3*H*)-quinazolinone (3-OH-C7-QZN)

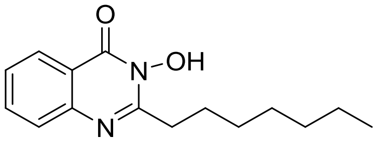

9

Hydroxylamine hydrochloride (0.278 g, 4 mmol) was added to a solution of sodium methoxide from sodium metal (0.0966 g, 4.1 mmol) in ethanol (20 ml). The suspension was stirred and the precipitated sodium chloride was removed by filtration. 2-*n*-Heptyl-4*H*-benzo[d][1,3]oxazin-4-one (as described for **6**) (0.985 g, 4 mmol) was added to the above filtrate and the mixture was then heated at reflux for 48 h. The solvent was removed under reduced pressure to give an oily solid which was purified by recrystallisation from ethanol to give the title compound as a cream solid (0.245 g, 23.5%).
mp 212-220 ^{o}C (Lit. mp 215-220 ^{o}C; [6])

^{1}H NMR (DMSO) δ 0.86 (3H, t, J = 6.6Hz, Me), 1.20-1.4 (8H, m, *$(CH_2)_4$*Me), 1.63 (2H,d, J = 8 Hz, *CH_2*$(CH_2)_4$Me), 2.26 (2H, t, J = 7.6Hz, *CH_2*$(CH_2)_5$Me), 6.90 (1H, 8-H), 7.22 (1H, 6-H), 7.96 (1H, 7-H), 8.44 (1H, 5-H), 14.31 (1H, s, OH)
ES-MS *m/z* 261.1630 $[M+H]^+$, $C_{15}H_{21}N_2O_2^+$ requires 261.1603)

3-Hydroxy-2-*n*-nonyl--4(3*H*)-quinazolinone (3-OH-C9-QZN)

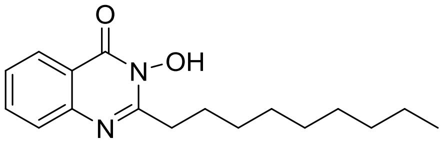

10

Hydroxylamine hydrochloride (0.208 g, 3 mmol) was added to a solution of sodium methoxide from sodium metal (0.069 g, 3.2 mmol) in ethanol (20 ml). The suspension was stirred and the precipitated sodium chloride was removed by filtration. 2-*n*-Nonyl-4*H*-benzo[d][1,3]oxazin-4-one (as described for **7**)(0.819 g, 3 mmol) was added to the above filtrate and the mixture was then heated at reflux for 48 h. The solvent was removed under reduced pressure to give an oily solid which was purified by trituration with hexane and ether followed by recrystallisation from ethanol to give the title compound as a white solid (0.021 g, 2.4%).
mp 93-94 ^{o}C

^{1}H NMR (DMSO-d_6) δ 0.86 (3H, t, J = 7Hz, Me), 1.20-1.5 (12H, m, *(CH_2)$_6$*Me), 1.74 (2H, m, *CH_2*(CH_2)$_6$Me), 2.84 (2H, t, J = 8 Hz, *CH_2*(CH_2)$_7$Me), 7.50 (1H, 8-H), 7.64 (1H, 6-H), 7.79 (1H, 7-H), 8.12 (1H, 5-H), 11.57 (1H, s, OH)
ES-MS *m/z* 289.1925 $[M+H]^+$, $C_{17}H_{25}N_2O_2^+$ requires 289.1916)

7-Chloro-3-hydroxy -2-*n*-nonyl -4(3*H*)-quinazolinone (3-OH-7Cl-C9-QZN)

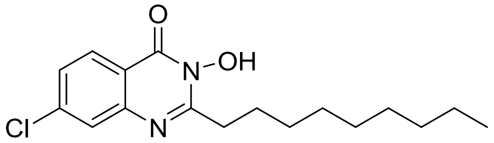

11

N-Decanoyl-4-chloroanthranilic acid was prepared as a pale brown solid in 82.7% yield by GP1 using 4-chloroanthranilic acid and decanoyl chloride and converted to 7-chloro-2-n-nonyl-4*H*-benzo[d][1,3]oxazin-4-one as a pale brown solid in quantitative yield by GP2.
Hydroxylamine hydrochloride (0.208 g, 3 mmol) was added to a solution of sodium methoxide from sodium metal (0.069 g, 3.2 mmol) in ethanol (20 ml). The suspension was stirred and the precipitated sodium chloride was removed by filtration. 2-*n*-Nonyl-7-chloro-4*H*-benzo[d][1,3]oxazin-4-one (0.923 g, 3 mmol) was added to the above filtrate and the mixture was heated at reflux for 48 h. The solvent was removed under reduced pressure to give a cream solid which was purified by trituration with ether and recrystallisation from ethanol to give the title compound as a white solid (0.020 g, 2.03%).
mp 107-109 ^oC
^{1}H NMR (DMSO-d_6) δ 0.85 (3H, t, J = 7 Hz, Me), 1.25-1.5 (12H, m, *(CH_2)$_6$*Me), 1.74 (2H,quintet, J = 7.7 Hz, *CH_2*(CH_2)$_6$Me), 2.84 (2H, t, J = 7.5 Hz, *CH_2*(CH_2)$_7$Me), 7.53 (1H, 6-H), 7.69 (1H, 8-H), 8.12 (1H, 5-H), 11.70 (1H, OH)
ES-MS *m/z* 323.1502 & 325.1573 $[M+H]^+$, $C_{17}H_{24}ClN_2O_2^+$ requires 323.1526 & 325.1497

7-Fluoro-3-hydroxy-2-*n*-nonyl-4(3*H*)-quinazolinone (3-OH-7F-C9QZN)

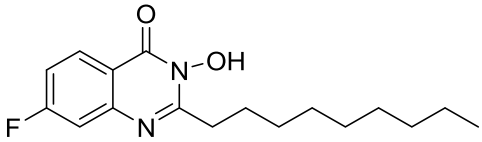

12

A solution of 2-*n*-nonyl-7-fluoro-4*H*-benzo[d][1,3]oxazin-4-one (as described for **8**) (0.291 g, 1 mmol) and hydroxylamine HCl (0.1043 g, 1.5 mmol) in dry pyridine (15 ml) was stirred at 110 ^oC for 18 h. The solvent was removed under reduced pressure to give an oily solid which was triturated with water (10 ml) .The resulting brown solid was filtered and washed with 1M HCl and water. The brown solid was purified by recrystallisation from ethanol to give the title product as an off white solid (0.092 g, 30.0%).
mp 100-102 ^oC

^{1}H NMR (DMSO-d$_6$) δ 0.86 (3H, t, J = 7 Hz, Me), 1.25-1.5 (12H, m, *(CH$_2$)$_6$*Me), 1.74 (2H,quintet, J = 7.7 Hz, *CH$_2$*(CH$_2$)$_6$Me), 2.84 (2H, t, J = 7.5 Hz, *CH$_2$*(CH$_2$)$_7$Me), 7.35-7.44 (2H, 6-H & 8-H), 8.18 (1H, 5-H), 11.65 (1H, OH)
ES-MS *m/z* 307.1815 [M+H]$^+$, $C_{17}H_{24}FN_2O_2^+$ requires 307.1822.

2-*n*-Heptyl-3-methoxy-4(3*H*)-quinazolinone (3-OMe-C7-QZN)

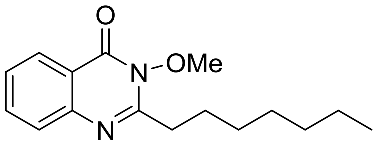

13

Methoxyamine hydrochloride (0.184 g, 2 mmol) was added to a solution of sodium methoxide from sodium metal (0.046 g, 2 mmol) in methanol (10 ml) followed by the addition of 2-*n*-heptyl-4*H*-benzo[d][1,3]oxazin-4-one (as described for **6**) (0.491 g, 2 mmol) and the solution was refluxed overnight. The solvent was removed under reduced pressure and the residue was dissolved in ethyl acetate and washed successively with water, 1 M HCl and brine. The organic layer was dried over magnesium sulphate and the solvent concentrated under reduced pressure to give a pale brown oil. The oil was triturated with hexane to give a pale brown solid. The solid was further purified using Flash chromatography (30% ethyl acetate in hexane) to deliver the title compound as a white solid (0.030 g, 5.5%).
mp 53-54 ^{o}C
^{1}H NMR (CDCl$_3$) δ 0.92 (3H, t, *J* = 7.0Hz, Me), 1.25-1.5 (8H, m, *(CH$_2$)$_4$* Me), 1.88 (2H, quintet, J = 7.6Hz,_*CH$_2$*(CH$_2$)$_4$Me), 2.90 (2H, t, J = 8Hz, *CH$_2$*(CH$_2$)$_5$Me), 4.14 (3H, s, NOMe), 7.47 (1H, 8-H), 7.70 (1H, 6-H), 7.75 (1H, 7-H), 8.27 (1H, 5-H)
ES-MS *m/z* 275.1765 [M+H]$^+$, $C_{16}H_{23}N_2O_2^+$ requires 275.1760.

3-Methoxy-2-*n*-nonyl-4(3*H*)-quinazolinone (3-OMe-C9-QZN)

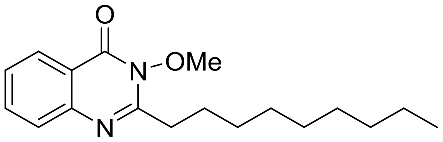

14

A solution of 2-*n*-nonyl-4*H*-benzo[*d*][1,3]oxazin-4-one (as described for **7**) (0.273 g, 1 mmol), methoxyamine HCl (0.092 g, 1.1 mmol) and triethylamine (0.111 g, 1.1 mmol) in dry THF (15 ml) was stirred at reflux overnight. The solvent was removed under reduced pressure and the residue was dissolved in ethyl acetate (20 ml) and washed with 1 M HCl (2 x 10 ml) and brine (10 ml). The organic layer was dried over magnesium sulphate and concentrated to dryness to give a cream solid which was purified using Flash chromatography (10% ethyl acetate in hexane) and then silica plate chromatography (50% ethyl acetate in hexane) to give the title compound as a white solid (0.040 g, 13.2%).
mp 51-52 ^{o}C

^{1}H NMR ($CDCl_3$) δ 0.91 (3H, t, J = 7 Hz, Me), 1.25-1.5 (12H, m, *(CH₂)₆*Me), 1.87 (2H, m, *CH₂*(CH₂)₆Me), 2.90 (2H, t, J = 7.8 Hz, *CH₂*(CH₂)₇Me), 4.14 (3H, s, OMe), 7.48 (1H, 8-H), 7.69 (1H, 6-H), 7.75 (1H, 7-H), 8.29 (1H, 5-H)

ES-MS *m/z* 303.2054 $[M+H]^+$, $C_{18}H_{27}N_2O_2^+$ requires 303.2073.

7-Chloro-3-methoxy-2-*n*-nonyl-4(*3H*)-quinazolinone (3-OMe-7Cl-C9-QZN)

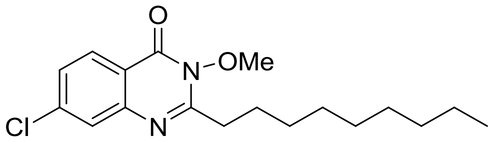

15

A solution of 7-chloro-2-*n*-nonyl-4*H*-benzo[*d*][1,3]oxazin-4-one (as described for **11**) (0.308 g, 1 mmol), methoxyamine HCl (0.092 g, 1.1 mmol) and triethylamine (0.111 g, 1.1 mmol) in dry THF (15 ml) was stirred at reflux overnight. The solvent was removed under reduced pressure and the residue was dissolved in ethyl acetate (20 ml) and washed with 1 M HCl (2 x 10 ml) and brine (10 ml). The organic layer was dried over magnesium sulphate and concentrated to dryness to give a cream solid which was purified using Flash chromatography (15% ethyl acetate in hexane) to give the title compound as a white solid (0.111 g, 33%).

mp 96-97 ^{o}C

^{1}H NMR ($CDCl_3$) δ 0.91 (3H, t, J = 7 Hz, Me), 1.25-1.5 (12H, m, *(CH₂)₆*Me), 1.86 (2H, m, *CH₂*(CH₂)₆Me), 2.88 (2H, t, J = 7.8 Hz, *CH₂*(CH₂)₇Me), 4.13 (3H, s, OMe), 7.42 (1H, 6-H), 7.69 (1H, 8-H), 8.21 (1H, 5-H)

ES-MS *m/z* 337.1700 & 339.1768 $[M+H]^+$, $C_{18}H_{26}ClN_2O_2^+$ requires 337.1683 & 339.1653.

7-Fluoro-3-methoxy-2-*n*-nonyl-4(3*H*)-quinazolinone (3-OMe-7F-C9-QZN)

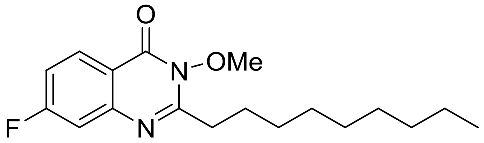

16

A solution of 7-fluoro-2-nonyl-4*H*-benzo[*d*][1,3]oxazin-4-one (as described for **8**) (0.291 g, 1 mmol), methoxyamine HCl (0.092 g, 1.1 mmol) and triethylamine (0.111 g, 1.1 mmol) in dry THF (15 ml) was stirred at reflux overnight. The solvent was removed under reduced pressure and the residue was dissolved in ethyl acetate (20 ml) and washed with 1 M HCl (2 x 10 ml) and brine (10 ml). The organic layer was dried over magnesium sulphate and concentrated to dryness to give a cream solid which was purified using Flash chromatography (15% ethyl acetate in hexane) and triturated with diethyl ether to give the title compound as a white solid (0.037 g).

mp 82-83 ^{o}C

^{1}H NMR ($CDCl_3$) δ 0.91 (3H, t, J = 7.0 Hz, Me), 1.25-1.5 (12H, m, *(CH_2)$_6$*Me), 1.86 (2H,quintet, J = 8 Hz, *CH_2*(CH_2)$_6$Me), 2.88 (2H, t, J = 8 Hz, *CH_2*(CH_2)$_7$Me), 4.13 (3H, s, OMe), 7.19 (1H, 6-H), 7.32 (1H, 8-H)), 8.29 (1H, 5-H)
ES-MS *m/z* 321.1960 $[M+H]^+$, $C_{18}H_{26}FN_2O_2^+$ requires 321.1978.

6,7-Difluoro-3-methoxy-2-*n*-nonyl-4(3*H*)-quinazolinone (3-OMe-6F, 7F-C9-QZN)

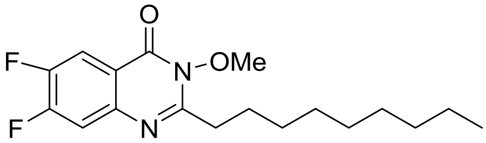

17

N-Decanoyl-4, 5-difluoroanthranilic acid was prepared as a pale brown solid in 44.8% yield using GP1 from 4,5-difluoroanthranilic acid and decanoyl chloride and converted to 6, 7-difluoro-2-*n*-nonyl-4*H*-benzo[*d*][1,3]oxazin-4-one as a pale brown solid in quantitative yield using GP2.

A solution of 6, 7-difluoro-2-*n*-nonyl-4*H*-benzo[*d*][1,3]oxazin-4-one (0.309 g, 1 mmol), methoxyamine HCl (0.092 g, 1.1 mmol) and triethylamine (0.111 g, 1.1 mmol) in dry THF (15 ml) was stirred at reflux overnight. The solvent was removed under reduced pressure and the residue was dissolved in ethyl acetate (20 ml) and washed with 1 M HCl (2 x 10 ml) and brine (10 ml). The organic layer was dried over magnesium sulphate and concentrated to dryness to give a yellow solid which was purified using Flash chromatography (10% ethyl acetate in hexane) to give the title compound as a white solid (0.032 g).
mp 75-76 ^{o}C
^{1}H NMR ($CDCl_3$) δ 0.91 (3H, t, J = 7.0 Hz, Me), 1.25-1.5 (12H, m, *(CH_2)$_6$*Me), 1.86 (2H,quintet, J = 8 Hz, *CH_2*(CH_2)$_6$Me), 2.87 (2H, t, J = 8 Hz, *CH_2*(CH_2)$_7$Me), 4.13 (3H, s, OMe), 7.46 (1H, 8-H), 8.03 (1H, 5-H)
ES-MS *m/z* 339.1789 $[M+H]^+$, $C_{18}H_{25}F_2N_2O_2^+$ requires 339.1884.

3-Amino-2-*n*-pentyl-4(3*H*)-quinazolinone (3-NH_2-C5-QZN)

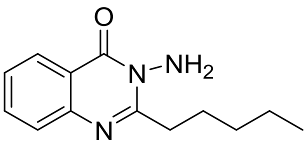

18

N-Hexanoylanthranilic acid was prepared as a pale brown solid in 84% yield by GP1 using anthranilic acid and hexanoyl chloride and converted to 2-*n*-pentylbenzo[*d*][1,3]oxazin-4-one as a brown oil in quantitative yield by GP2.
2-*n*-Pentyl-4*H*-benzo[d][1,3]oxazin-4-one by **GP3** afforded the title compound as a cream solid in 97.4% yield which required no further purification.
mp 60-62 ^{o}C (Lit. mp 60-61 ^{o}C; [6])

^{1}H NMR ($CDCl_3$) δ 0.96 (3H, t, J = 7.2 Hz, Me), 1.25-1.5 (4H, m, *$(CH_2)_2$*Me), 1.85 (2H,m, *CH_2*$(CH_2)_2$Me), 3.05 (2H, m, *CH_2*$(CH_2)_3$Me), 4.87 (2H, s, NH_2), 7.47 (1H, 8-H), 7.70 (1H, 6-H), 7.75 (1H, 7-H), 8.27 (1H, 5-H)
ES-MS *m/z* 232.1441 $[M+H]^+$, $C_{13}H_{18}N_3O^+$ requires 232.1450.

3-Amino-2-*n*-heptyl-4(3*H*)-quinazolinone (3-NH_2-C7-QZN)

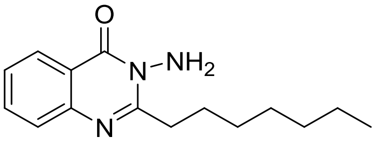

19

2-*n*-Heptyl-4*H*-benzo[d][1,3]oxazin-4-one (as described for **6**) by **GP3** afforded the title compound as a cream solid which was recrystallised from ethanol/water to give an off white solid (81.3%).
mp 74-75 ^{o}C (Lit. mp 74-75 ^{o}C; [6])
^{1}H NMR ($CDCl_3$) δ 0.92 (3H, t, J = 7.0 Hz, Me), 1.25-1.5 (8H, m, *$(CH_2)_4$*Me), 1.84 (2H, quintet, J = 7.6 Hz, *CH_2*$(CH_2)_4$Me), 3.05 (2H, t, J = 7.6 Hz, *CH_2*$(CH_2)_5$Me), 4.87 (2H, s, NH_2), 7.47 (1H, 8-H), 7.70 (1H, 6-H), 7.75 (1H, 7-H), 8.27 (1H, 5-H)
ES-MS *m/z* 260.1780 $[M+H]^+$, $C_{15}H_{22}N_3O^+$ requires 260.1763.

3-Amino-2-*n*-nonyl-4(3*H*)-quinazolinone (3-NH_2-C9-QZN)

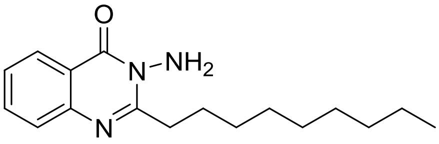

20

A solution of 2-nonyl-4*H*-benzo[d][1,3]oxazin-4-one (as described for **7**) by **GP3** afforded the title compound as a cream solid in a quantitative yield which required no further purification.
mp 56-58 ^{o}C (Lit. mp 57-58 ^{o}C; [6])
^{1}H NMR ($CDCl_3$) δ 0.91 (3H, t, J = 7 Hz, Me), 1.25-1.5 (12H, m, *$(CH_2)_6$*Me), 1.85 (2H,quintet, J = 7.64 Hz, *CH_2*$(CH_2)_6$Me), 3.04 (2H, t, J = 8 Hz, *CH_2*$(CH_2)_7$Me), 4.87 (2H, s, NH_2), 7.47 (1H, 8-H), 7.70 (1H, 6-H), 7.75 (1H, 7-H), 8.27 (1H, 5-H)
ES-MS *m/z* 288.2054 $[M+H]^+$, $C_{17}H_{26}N_3O^+$ requires 288.2076.

3-Amino-2-undecanyl-4(3*H*)-quinazolinone (3-NH_2-C11-QZN; [11])

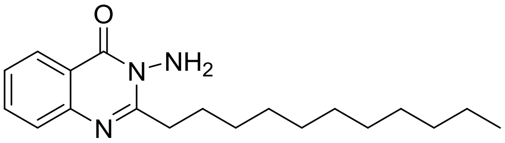

21

N-Undecanoylanthranilic acid was prepared as a pale brown solid in 75% yield by GP1 using anthranilic acid and undecanoyl chloride and converted to 2-undecanyl-4*H*-benzo[*d*][1,3]oxazin-4-one as a brown oil in quantitative yield by GP2.

2-Undecanyl-4*H*-benzo[d][1,3]oxazin-4-one by **GP3** afforded the title quinazolinone **21** as a cream solid in a quantitative yield which required no further purification.

mp 62-64 ^{o}C

^{1}H NMR ($CDCl_3$) δ 0.91 (3H, t, J = 7 Hz, Me), 1.25-1.5 (16H, m, *(CH_2)*$_8$Me), 1.85 (2H,quintet, J = 7.64 Hz, *CH_2*(CH_2)$_8$Me), 3.04 (2H, t, J = 8 Hz, *CH_2*(CH_2)$_9$Me), 4.87 (2H, s, NH_2), 7.47 (1H, 8-H), 7.70 (1H, 6-H), 7.75 (1H, 7-H), 8.27 (1H, 5-H)

ES-MS *m/z* 316.2434 $[M+H]^+$, $C_{19}H_{30}N_3O^+$ requires 316.2389.

3-Amino-6-chloro-2-*n*-nonyl-4(3*H*)-quinazolinone (3-NH_2-6Cl-C9-QZN)

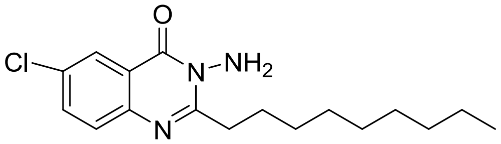

22

N-Decanoyl-5-chloroanthranilic acid was prepared as a pale brown solid in 71% yield by GP1 using 5-chloroanthranilic acid and decanoyl chloride and converted to 6-chloro-2-*n*-nonyl-4*H*-benzo[*d*][1,3]oxazin-4-one as a pale brown solid in quantitative yield by GP2.

The title compound was prepared by GP3 from 6-chloro-2-*n*-nonyl-4*H*-benzo[*d*][1,3]oxazin-4-one. It was purified by recrystallsation twice from ethyl acetate to give a white solid in 29.5% yield.

mp 91-93 ^{o}C

^{1}H NMR ($CDCl_3$) δ 0.91 (3H, t, J = 7.Hz, Me), 1.25-1.5 (12H, m, *(CH_2)*$_6$Me), 1.85 (2H,quintet, J = 8 Hz, *CH_2*(CH_2)$_6$Me), 3.03 (2H, t, J = 8 Hz, *CH_2*(CH_2)$_7$Me), 4.87 (2H, s, NH_2), 7.67 (2H, 7-H & 8-H), 7.75 (1H, 7-H), 8.22 (1H, 5-H)

ES-MS *m/z* 322.1699 & 324.1740 $[M+H]^+$, $C_{17}H_{25}ClN_3O^+$ requires 322.1686 & 324.1657.

3-Amino-7-chloro-2-*n*-nonyl-4(3*H*)-quinazolinone (3-NH_2-7Cl-C9-QZN)

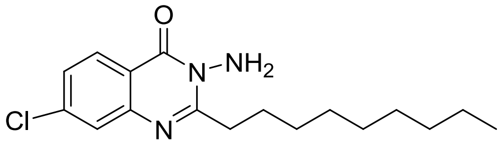

23

7-Chloro-2-n-nonyl-4*H*-benzo[d][1,3]oxazin-4-one (as described for **11**) by **GP3** afforded the title compound as a cream solid which was purified by recrystallisation from ethyl acetate to give an off white solid (Yield 35.4%).

mp 79-80 ^oC

^1H NMR ($CDCl_3$) δ 0.91 (3H, t, J = 7.0 Hz, Me), δ 1.25-1.5 (12H, m, $(CH_2)_6$Me), 1.84 (2H,quintet, J = 8 Hz, $CH_2(CH_2)_6$Me), 3.03 (2H, t, J = 8 Hz, $CH_2(CH_2)_7$Me), 4.85 (2H, NH_2), 7.42 (1H, 8-H), 7.70 (1H, 6-H), 8.19 (1H, 5-H)

ES-MS *m/z* 322.1712 & 324.1667 $[M+H]^+$, $C_{17}H_{25}ClN_3O^+$ requires 322.1686 & 324.1657.

3-Amino-6-fluoro-2-*n*-nonyl-4(3*H*)-quinazolinone (3-NH_2-6F-C9-QZN)

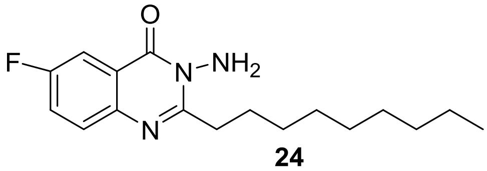

24

The precursor methyl 2-(decanamido)-5-fluorobenzoate was prepared by the following method.

Decanoyl chloride (1.11 g, 11 mmol) was added dropwise over 10 min to a solution of methyl 2-amino-5-fluorobenzoate (1.69 g, 10 mmol) and triethylamine (1.11 g, 11 mmol) in dry THF (20 ml) at room temperature. Stirred overnight at room temperature and then solvent was removed under vacuum. The residue was dissolved in ethyl acetate (20 ml) washed with saturated sodium bicarbonate (2 x 10 ml), 1 M HCl (2 x 10 ml) and brine (10 ml). The organic layer was dried with magnesium sulphate and the solvent was removed under vacuum to give a yellow solid (2.782 g, 86.1%).

A solution of methyl 2-(decanamido)-5-fluorobenzoate (0.323 g, 1 mmol) and hydrazine monohydrate (0.2 ml, 4 mmol) in butanol (7.5 ml) was stirred at reflux overnight. The solvent was removed under reduced pressure to give a solid which was firstly purified by stirring in hexane and isolated by filtration. The material was then dissolved in ethyl acetate and washed with saturated sodium bicarbonate and brine, dried with magnesium sulphate and the solvent was removed under vacuum to give a cream solid. The cream solid was triturated with diethyl ether to give the title compound as an off white solid. (0.089 g, 29.2%)

mp 83-85 ^oC

^{1}H NMR ($CDCl_3$) δ 0.90 (3H, t, J = 7 Hz, Me), 1.25-1.5 (12H, m, *(CH₂)*₆Me), 1.84 (2H, m, *CH₂*(CH₂)₆Me), 3.03 (2H, t, J = 8 Hz, *CH₂*(CH₂)₇Me), 4.87 (2H, s, NH₂), 7.48 (1H, 8-H), 7.70 (1H, 7-H), 7.89 (1H, 5-H)

ES-MS *m/z* 306.1975 $[M+H]^+$, $C_{17}H_{25}FN_3O^+$ requires 306.1982.

3-Amino-7-fluoro-2-*n*-nonyl-4(3*H*)-quinazolinone (3-NH₂-7F-C9-QZN)

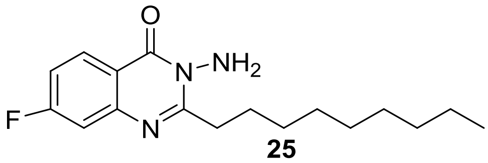

25

The title compound was prepared by GP3 from 7-fluoro-2-*n*-nonyl-4*H*-benzo[*d*][1,3]oxazin-4-one (as described for **8**). It was purified by Flash chromatography (15% ethyl acetate in hexane) to give a white solid (34.8%)

mp 73-74 °C

^{1}H NMR ($CDCl_3$) δ 0.91 (3H, t, J = 7.Hz, Me), δ 1.25-1.5 (12H, m, *(CH₂)*₆Me), 1.85 (2H,quintet, J = 8 Hz, *CH₂*(CH₂)₆Me), 3.03 (2H, t, J = 8 Hz, *CH₂*(CH₂)₇Me), 4.84 (2H, s, NH₂), 7.18 (1H, 8-H), 7.33 (1H, 6-H), 8.27 (1H, 5-H)

ES-MS *m/z* 306.1961 $[M+H]^+$, $C_{17}H_{25}FN_3O^+$ requires 306.1982.

3-Amino-6,7-difluoro-2-*n*-nonyl-4(3*H*)-quinazolinone (3-NH₂-6F,7F-C9QZN)

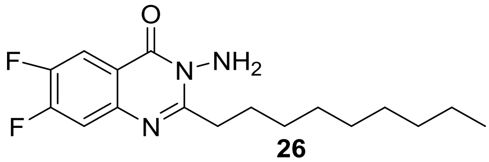

26

The title compound was prepared by GP3 from 6,7-difluoro-2-*n*-nonyl-4*H*-benzo[*d*][1,3]oxazin-4-one (as described for **17**). The crude yellow solid was purified using Flash chromatography (15% ethyl acetate in hexane) to give the title compound as a white solid. (60.1%)

mp 75-77 °C

^{1}H NMR ($CDCl_3$) δ 0.91 (3H, t, J = 7.0Hz, Me), 1.25-1.5 (12H, m, *(CH₂)*₆Me), 1.85 (2H,quintet, J = 8 Hz, *CH₂*(CH₂)₆Me), 3.02 (2H, t, J = 8 Hz, *CH₂*(CH₂)₇Me), 4.85 (2H, s, NH₂), 7.47 (2H, 8-H), 8.01 (1H, 5-H)

ES-MS *m/z* 324.1869 $[M+H]^+$, $C_{17}H_{24}F_2N_3O^+$ requires 324.1887.

3-Amino-7-(trifluoromethyl)-2-*n*-nonyl-4(3*H*)-quinazolinone (3-NH_2-7CF_3-C9-QZN)

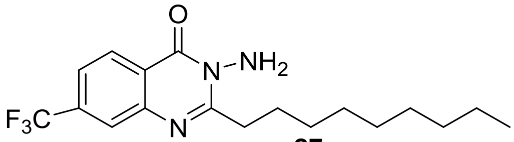

27

N-Decanoyl-4-(trifluoromethyl)anthranilic acid was prepared as a pale brown solid in 51.5% yield using GP1 using 4-(trifluoromethyl)anthranilic acid and decanoyl chloride and converted to 7-(trifluoromethyl)-2-*n*-nonyl-4*H*-benzo[*d*][1,3]oxazin-4-one as a pale brown solid in quantitative yield using by GP2.

The title compound was prepared by GP3 from 7-(trifluoromethyl)-2-*n*-nonyl-4*H*-benzo[*d*][1,3]oxazin-4-one. It was purified by Flash chromatography (15% ethyl acetate in hexane) to give a white solid (62.5%).

mp 71-72 ^{o}C

^{1}H NMR ($CDCl_3$) δ 0.92 (3H, t, J = 7.0Hz, Me), 1.25-1.5 (12H, m, *(CH_2)*$_6$Me), 1.85 (2H,quintet, J = 8 Hz, *CH_2*(CH_2)$_6$Me), 3.06 (2H, t, J = 8 Hz, *CH_2*(CH_2)$_7$Me), 4.90 (2H, s, NH_2), 7.67 (1H, 6-H), 8.00 (1H, 8-H), 8.38 (1H, 5-H)

ES-MS *m/z* 356.1917 $[M+H]^+$, $C_{18}H_{25}F_3N_3O^+$ requires 356.1950.

3-Amino-6,7-dimethoxy-2-*n*-nonyl-4(3*H*)-quinazolinone (3-NH_2-6OMe, 7OMe -C9-QZN)

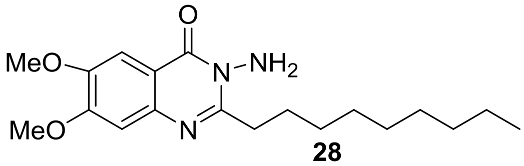

28

N-Decanoyl-4,5-dimethoxyanthranilic acid was prepared as a pale brown solid in 81.8% yield using GP1 using 4,5-dimethoxyanthranilic acid and decanoyl chloride and converted to 6,7-dimethoxy-2-*n*-nonyl-4*H*-benzo[*d*][1,3]oxazin-4-one as a pale brown solid in quantitative yield using by GP2.

The title compound was prepared by GP3 from 6,7-dimethoxy-2-*n*-nonyl-4*H*-benzo[*d*][1,3]oxazin-4-one It was purified by Flash chromatography (50% ethyl acetate in hexane) to give a white solid (25.3%)

mp 87-89 ^{o}C

^{1}H NMR ($CDCl_3$) δ 0.90 (3H, t, J = 7.0Hz, Me), 1.25-1.5 (12H, m, *(CH_2)*$_6$Me), 1.83 (2H,quintet, J = 8 Hz, *CH_2*(CH_2)$_6$Me), 3.01 (2H, t, J = 8 Hz, *CH_2*(CH_2)$_7$Me), 4.02(6H, d, 2x OMe), 4.86 (2H, s, NH_2), 7.09 (2H, 8-H), 7.55 (1H, 5-H)

ES-MS *m/z* 348.2295 $[M+H]^+$, $C_{19}H_{30}N_3O_3^+$ requires 348.2287.

3-Amino-2-(3-methyloctyl)-4(3*H*)-quinazolinone (3-NH$_2$-(3-Me-C8)-QZN)

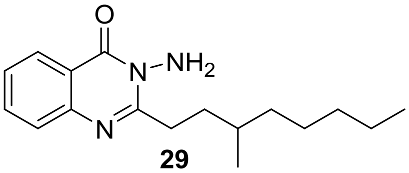

29

4-Methylnonanoyl chloride was prepared by GP4 using 4-methylnonanoic acid.

N-(4-Methylnonanoyl)anthranilic acid was prepared as a pale brown solid in 66.2% yield by GP1 using anthranilic acid and 4-methylnonanoyl chloride and converted to 2-(3-methyloctyl)-4*H*-benzo[*d*][1,3]oxazin-4-one as a pale brown oil in quantitative yield by GP2.

The title compound was prepared by GP3 from 2-(3-methyloctyl)-4*H*-benzo[*d*][1,3]oxazin-4-one. It was purified by Flash chromatography (20% ethyl acetate in hexane) to give a cream/white solid (18.1%).

mp 67-68 ^{o}C

^{1}H NMR ($CDCl_3$) δ 0.91 (3H, t, J = 7 Hz, $(CH_2)_4$*Me*), 1.01 (3H, d, J = 6.5 Hz, CH_2H*Me* CH_2), 1.25-1.5 (8H, m, *(CH$_2$)$_4$*Me), 1.65 (2H, m, CH_2*CH$_2$*HMe), 1.87 (1H ,m , CH_2CH_2*H*Me), 3.05 (2H, m, *CH$_2$*CH_2HMe), 4.87 (2H, s, NH_2), 7.47 (1H, 8-H), 7.70 (1H, 6-H), 7.74 (1H, 7-H), 8.26 (1H, 5-H)

ES-MS *m/z* 288.2092 $[M+H]^+$, $C_{17}H_{26}N_3O^+$ requires 288.2076.

3-Amino-2-(7-methyloctyl)-4(3*H*)-quinazolinone (3-NH$_2$-(7-Me-C8)-QZN)

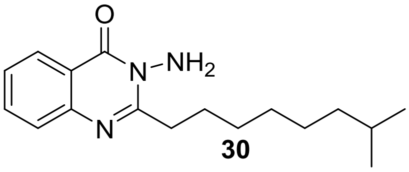

30

8-Methylnonanoic acid was prepared by the following procedure.

8-Methyl-7-oxononanoic acid (4.5 g, 0.0242 mol), hydrazine monohydrate (7.71 ml), potassium hydroxide pellets (1.59 g, 0.0242 mol) and diethylene glycol (23 ml) were stirred at reflux for 7h. Further potassium hydroxide pellets (7.98 g, 0.121 mol) and diethylene glycol (23 ml) was added and the mixture was stirred at reflux for 16 h. The mixture was cooled, poured into ice water (400 ml) and acidified with conc. HCl (25 ml). The white precipitate was collected by filtration and washed with cold water to afford 8-Methylnonanoic acid in 75.4% yield)

8-Methylnonanoyl chloride was prepared by GP4 using 8-methylnonanoic acid.

N-(8-Methylnonanoyl)anthranilic acid was prepared as a pale brown solid in 78.6% yield using GP1 using anthranilic acid and 8-methylnonanoyl chloride and converted to 2-(7-methyloctyl)-4*H*-benzo[*d*][1,3]oxazin-4-one as a pale brown oil in quantitative yield using by GP2.

The title quinazolinone was prepared by GP3 from 2-(8-methyloctyl)-4*H*-benzo[*d*][1,3]oxazin-4-one. Purification was by Flash chromatography (20% ethyl acetate in hexane).The compound was then dissolved in ethyl acetate and washed with saturated sodium bicarbonate (several times) and brine, dried with magnesium sulphate and the solvent was removed under reduced pressure to give **30** as a cream/white solid. (15.4%)

mp 69-71 ^{o}C

^{1}H NMR ($CDCl_3$) δ 0.89 (6H, d, Me_2), 1.18-1.55 (9H, m, $(CH_2)_4HMe_2$), 1.84 (2H ,m , $CH_2(CH_2)_4Me_2$), 3.05 (2H, m, $CH_2(CH_2)_5$), 4.87 (2H, s, NH_2), 7.47 (1H, 8-H), 7.70 (1H, 6-H), 7.75 (1H, 7-H), 8.26 (1H, 5-H)

ES-MS *m/z* 288.2088 $[M+H]^+$, $C_{17}H_{26}N_3O^+$ requires 288.2076.

3-Amino-2-[(*E*)-3-nonenyl]-4(3*H*)-quinazolinone (3-NH$_2$-C9:3-QZN)

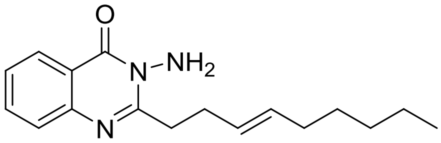

31

trans-4-Decenoyl chloride was prepared from *trans*-4-decenoic acid by GP4.

N-[(*E*)-4-Decenoyl]anthranilic acid was prepared as a pale brown solid in 57% yield by GP1 using anthranilic acid and *trans*-4-decenoyl chloride and converted to 2-[(*E*)-4-decenoyl]-4*H*-benzo[*d*][1,3]oxazin-4-one by GP2 in a quantitative yield.

The title compound was prepared by GP3 using 2-[(*E*)-4-decenoyl) -4*H*-benzo[*d*][1,3]oxazin-4-one. It was purified by Flash chromatography (30% ethyl acetate in hexane) and then recrystallised from ethyl acetate to give a white solid (14.0%)

mp 60-61 ^{o}C

^{1}H NMR ($CDCl_3$) δ 0.89 (3H, t, J = 7.2 Hz, Me), 1.2-1.35 (6H, m, $(CH_2)_3Me$), 2.02 (2H, m, $CH_2(CH_2)_3Me$), 2.55 (2H, m, CH_2CH_2CHCH), 3.13 (2H, quintet, J =6.2 Hz, $CH_2CH_2CH{=}CH$), 4.88 (2H, s, NH_2), 5.55 (2H, m, CH=CH), 7.47(1H, 8-H), 7.71 (1H, 6-H), 7.75 (1H, 7-H), 8.27 (1H, 5-H)

ES-MS *m/z* 286.1919 $[M+H]^+$, $C_{17}H_{24}N_3O^+$ requires 286.1919.

3-Amino-2-[(2-butoxyethoxy)methyl]-4(3*H*)-quinazolinone (3-NH$_2$-(2, 5-dioxa-C9)-QZN

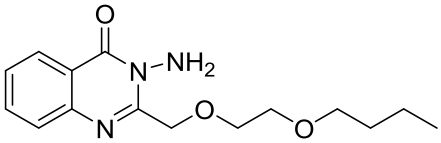

32

(2-Butoxyethoxy)acetic acid was prepared by the following method,

Chromic acid (26 ml) was added to a solution of diethylene glycol butyl ether (5 g, 0.01 M) in acetone until a red colour persisted. The mixture was stirred at room temperature for 2 h. Isopropanol (45 ml) was added until the mixture turned green. Insoluble inorganics were removed by filtration and the filtrate was concentrated under reduced pressure to give a dark green oil. The oil was initially purified by dissolving in ethyl acetate (50 ml and washing the solution with water (3 x 25 ml) and brine (20 ml), dried over magnesium sulphate and concentrated under reduced pressure to give a green oil (2.567 g). The oil was then purified by dissolving in saturated $NaHCO_3$ (30 ml) and washing with ethyl acetate (2 x 20 ml). The aqueous layer was acidified with cHCl (4 ml) and extracted with ethyl acetate (2 x 25 ml), which was dried over magnesium sulphate and concentrated under reduced pressure to give (2-butoxyethoxy)acetic acid as a clear oil (1.574 g, 29%).

^{1}H NMR ($CDCl_3$) δ 0.95 (3H, t, Me), 1.4 (2H, m, CH_2Me), 1.63 (2H, m, CH_2CH_2Me), 3.57 (2H, m, $CH_2(CH_2)_2$Me), 3.65 (2H, t, $OCH_2CH_2OCH_2COOH$), 3.79 (2H, t, $OCH_2CH_2OCH_2COOH$), 4.19 (2H, s, CH_2COOH)

(2-Butoxyethoxy)acetyl chloride was prepared by GP4 using (2-butoxyethoxy)acetic acid.

N-[(2-Butoxyethoxy)acetyl]anthranilic acid was prepared as a pale brown solid in 75% yield by GP1 using anthranilic acid and (2-butoxyethoxy)acetyl chloride and converted to 2-[(2-butoxyethoxy)methyl]benzo[*d*][1,3]oxazin-4-one as a yellow oil in quantitative yield by GP2.

The title compound was prepared by GP3 using 2-[(2-butoxyethoxy)methyl]-4*H*-benzo[*d*][1,3]oxazin-4-one. It was purified by Flash chromatography (20% ethyl acetate in hexane followed by 30% ethyl acetate in hexane) and then recrystallised from ethyl acetate/pet ether 60-80 to give the title quinazolinone **32** as a white solid (Yield = 0.067 g, 23.0%).

mp 62-64 ^{o}C

^{1}H NMR ($CDCl_3$) δ 0.91 (3H, t, J = 7.4 Hz, Me), 1.34 (2H, m, $(CH_2)_2CH_2$Me), 1.56 (2H, m, $CH_2CH_2CH_2$Me), 3.44 (2H, t, J = 6.7Hz $CH_2(CH_2)_2$Me), 3.62 (2H, quintet, J = 3Hz, OCH_2CH_2O), 3.82 (2H, quintet, J = 2.7Hz, OCH_2CH_2O), 4.84 (2H, S, $CH_2OCH_2CH_2O$), 5.72 (2H, S, NH_2), 7.53 (1H, 8-H), 7.76 (2H, 6-H and 7-H), 8.30 (1H, 5-H)

ES-MS *m/z* 292.1635 $[M+H]^+$, $C_{15}H_{22}N_3O_3^+$ requires 292.1661.

3-Acetamido-2-*n*-nonyl-4(3*H*)-quinazolinone (3-NHAc-C9-QZN)

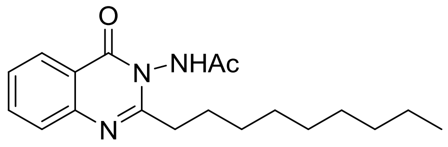

33

A solution of 3-Amino-2-*n*-nonyl-4(3*H*)-quinazolinone **20** (0.044 g, 0.153 mol), pyridine (0.3 ml) and acetic anhydride (3 ml) were stirred overnight at 35 to 40 ^{o}C. The solvent was removed under reduced pressure. The residue was dissolved in ethyl acetate (15 ml) and washed with saturated $NaHCO_3$ (2 x 10 ml), 1 M HCl (2 x 10 ml) and brine (10 ml). The

organic layer was dried over magnesium sulphate and concentrated under reduced pressure to give *3-diacetylamino-2-n-nonyl-4(3H)-quinazolinone* as a solid. (Yield = 0.041 g, 72.0%)
^{1}H NMR ($CDCl_3$) δ 0.91 (3H, t, *J* = 7.Hz, Me), δ 1.25-1.5 (12H, m, *$(CH_2)_6$*Me), 1.84 (2H,quintet, *J* = 7.5 Hz, *CH_2*$(CH_2)_6$Me), 2.44 (6H, d, $N(COMe)_2$), 2.55 (2H, m, *CH_2*$(CH_2)_7$Me), 7.52 (1H, 8-H), 7.75 (1H, 6-H), 7.82 (1H, 7-H), 8.26 (1H, 5-H)

A solution of 3-diacetylamino-2-*n*-nonyl-4(*3H*)-quinazolinone (0.031 g, 0.0835 mmol), 1 M NaOH (0.2 ml, 0.2 mmol), water (0.1 ml), and THF (0.25 ml) was stirred overnight at room temperature. The THF was removed under reduced pressure, water (2 ml) was added and the mixture acidified to pH 1 with 1 M HCl (0.25 ml) and extracted with ethyl acetate (2 x 5 ml). The organic extract was washed with brine and dried over magnesium sulphate and concentrated to give a glassy solid which was purified by trituration with petroleum ether 60-80 to give the title compound, 3-acetamido-2-n-nonyl-4(3*H*)-quinazolinone **33** as a white solid (0.010 g, 36.4%).
mp 73-76 ^oC
^{1}H NMR ($CDCl_3$) δ 0.91 (3H, t, J = 7.Hz, Me), δ 1.25-1.5 (12H, m, *$(CH_2)_6$*Me), 1.82 (2H,quintet, J = 7.6 Hz, *CH_2*$(CH_2)_6$Me), 2.78 (2H, m, *CH_2*$(CH_2)_7$Me), 7.47 (1H, 8-H), 7.71 (1H, 6-H), 7.77 (1H, 7-H), 8.01 (1H, s, NH), 8.23 (1H, 5-H)
ES-MS *m/z* 330.2180 $[M+H]^+$, $C_{19}H_{28}N_3O_2^+$ requires 330.2182.

2-*n*-Heptyl-3-dimethylamino-4(3*H*)-quinazolinone (3-NMe_2-C7-QZN)

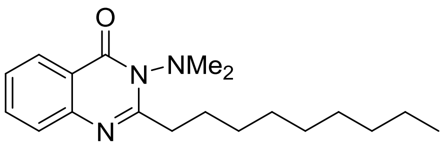

34

A solution of 2-*n*-heptyl-4*H*-benzo[d][1,3]oxazin-4-one (as described for **6**) (0.1225 g, 0.5 mmol) and *N*,*N*-dimethylhydrazine (0.12 g, 2 mmol) in dry tetrahydrofuran (10 ml) was stirred at reflux overnight. The solvent was removed under reduced pressure to give an oily residue. The crude oil was purified using Flash chromatography (20% ethyl acetate in hexane) to give the title compound as a white solid (0.050 g, 34.7%).
mp 60-62 ^oC
^{1}H NMR ($CDCl_3$) δ 0.92 (3H, t, J = 7.0Hz, Me), 1.25-1.5 (8H, m, *$(CH_2)_4$*Me), 1.81 (2H, quintet, J = 7.6Hz,_*CH_2*$(CH_2)_4$Me), 2.92 (2H, t, J = 8Hz, *CH_2*$(CH_2)_5$Me), 3.1 (6H, s, NMe_2), 7.43 (1H, 8-H), 7.64 (1H, 6-H), 7.71 (1H, 7-H), 8.24 (1H, 5-H)
ES-MS *m/z* 288.2036 $[M+H]^+$, $C_{17}H_{26}N_3O^+$ requires 288.2076.

3-(2-Aminoethyl)-2-*n*-nonyl-4(3*H*)-quinazolinone (3-C2NH$_2$-C9-QZN)

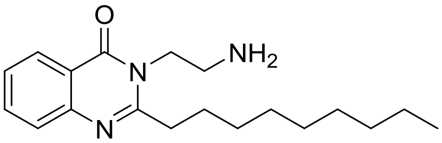

35

A solution of 2-*n*-nonyl-4*H*-benzo[d][1,3]oxazin-4-one (as described for **7**) (0.273 g, 1 mmol) and *N*-Boc-ethylenediamine (0.64g, 4 mmol) in dry THF (12 ml) was stirred at reflux overnight. The solvent was removed under reduced pressure and the residue was dissolved in ethyl acetate (25 ml) and washed with saturated $NaHCO_3$ (2 x 10 ml), 1 M $KHSO_4$ (2 x 10 ml) and brine (10 ml). The organic layer was dried over magnesium sulphate and concentrated to dryness to give a clear oil which was purified using Flash chromatography (20% ethyl acetate in hexane) to give *3-[2-(tert-butoxycarbonylamino)ethyl]-2-n-nonyl-4(3H)-quinazolinone* as a white solid (0.141 g).

^{1}H NMR ($CDCl_3$) δ 0.91 (3H, t, *J* = 7.2 Hz, Me), 1.25-1.5 (21H, m, *(CH$_2$)$_6$*Me and C*(CH$_3$)$_3$*), 1.83 (2H, q, J = 7.4 Hz, *CH$_2$*(CH$_2$)$_6$Me), 2.92 (2H, t, J = 7.4 Hz, *CH$_2$*(CH$_2$)$_7$Me), 3.50 (2H, m, CH$_2$*CH$_2$*NH), 4.27 (2H, t, J = 7.4 Hz, *CH$_2$*CH$_2$NH), 4.29 (1H, b, NH) 7.45 (1H, 8-H), 7.66 (1H, 6-H), 7.74 (1H, 7-H), 8.25 (1H, 5-H).

A solution of 3-[2-(*tert*-butoxycarbonylamino)ethyl]-2-*n*-nonyl-4(3*H*)-quinazolinone (0.100 g, 0.24 mmol) in DCM (6 ml) and trifluoroacetic acid (5 ml) was stirred overnight at room temperature. The solution was concentrated to dryness under reduced pressure with the aid of acetonitrile. The residue was dissolved in ethyl acetate and washed with saturated sodium bicarbonate and brine. The organic layer was dried over magnesium sulphate and concentrated to dryness to give the title compound **35** as a white solid (0.035 g, 46.1%).

mp 77-81 ^{o}C

^{1}H NMR ($CDCl_3$) δ 0.90 (3H, t, J = 6.7 Hz, Me), 1.20-1.55 (12H, m, *(CH$_2$)$_6$*Me), 1.85 (2H, quintet, J = 7.7 Hz, *CH$_2$*(CH$_2$)$_6$Me), 2.11 (2H, s, NH$_2$), 2.88 (2H, t, J = 7.8 Hz, *CH$_2$*(CH$_2$)$_7$Me), 3.11 (2H, s, *CH$_2$*NH$_2$), 4.22 (2H, s, J = 6.8 Hz, *CH$_2$* CH$_2$NH$_2$), 7.44 (1H, 8-H), 7.65 (1H, 6-H), 7.72 (1H, 7-H), 8.27 (1H, 5-H)

ES-MS *m/z* 316.2391 $[M+H]^+$, $C_{19}H_{30}N_3O^+$ requires 316.2389.

3-(2-Aminoethyl)-6-chloro-2-*n*-nonyl-4(3*H*)-quinazolinone (3-C2NH$_2$-6Cl-C9-QZN)

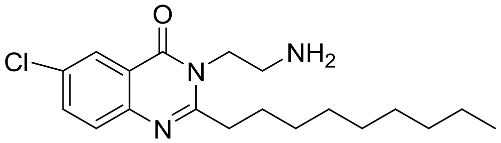

36

A solution of 2-nonyl-6-chloro-4*H*-benzo[d][1,3]oxazin-4-one (as described for **22**) (0.557 g, 1.81 mmol) and *N*-Boc-ethylenediamine (0.64 g, 7.24 mmol) in dry THF (22 ml) was stirred at reflux overnight. The solvent was removed under reduced pressure and the residue was

dissolved in ethyl acetate (40 ml) and washed with saturated $NaHCO_3$ (2 x 15 ml), 1 M $KHSO_4$ (2 x 15 ml) and brine (15 ml). The organic layer was dried over magnesium sulphate and concentrated to dryness to give a clear oil which was purified using Flash chromatography (20% ethyl acetate in hexane) to give *3-[2-(tert-butoxycarbonylamino)ethyl]-6-chloro-2-*n-*nonyl-4(3H)-quinazolinone* as a white solid (0.166 g, 20.4%).

A solution of 3-[2-(*tert*-butoxycarbonylamino)ethyl]-6-chloro-2-*n*-nonyl-4(3*H*)-quinazolinone (0.115 g, 3.4 mmol) in DCM (8 ml) and trifluoroacetic acid (8 ml) was stirred overnight at room temperature. The solution was concentrated to dryness under reduced pressure with the aid of acetonitrile. The residue was dissolved in ethyl acetate (15 ml) and washed with saturated sodium bicarbonate (2 x 15 ml) and brine (10 ml). The organic layer was dried over magnesium sulphate and concentrated to dryness to give an off white solid which was purified by trituration with diethyl ether to give the title compound **36** as a white solid (0.024 g, 20%).

mp 72-74 ^{o}C

^{1}H NMR ($CDCl_3$) δ 0.92 (3H, t, J =7 Hz, Me), 1.20-1.55 (14H, m, NH_2 & *(CH₂)*₆Me), 1.85 (2H, quintet, J = 7.9 Hz, *CH₂*(CH₂)₆Me), 2.88 (2H, t, J = 7.8 Hz, *CH₂*(CH₂)₇Me), 3.08 (2H, t, J = 7.42 Hz, *CH₂*NH₂), 4.20 (2H, t, J = 6.9 Hz, *CH₂*CH₂NH₂), 7.59 (1H, 8-H), 7.66 (1H, 7-H), 8.22 (1H, 5-H)

ES-MS *m/z* 350.2016 & 352.1854 $[M+H]^+$, $C_{19}H_{29}ClN_3O^+$ requires 350.1999 & 352.1970.

3-(2-Aminoethyl)-7-chloro-2-*n*-nonyl-4(3*H*)-quinazolinone (3-C2NH$_2$-7Cl-C9-QZN)

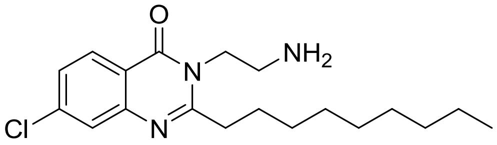

37

A solution of 7-chloro-2-nonyl-4*H*-benzo[d][1,3]oxazin-4-one (as described for **11**) (0.615 g, 2 mmol) and *N*-Boc-ethylenediamine (1.28 g, 8 mmol) in dry THF (24 ml) was stirred at reflux overnight. The solvent was removed under reduced pressure and the residue was dissolved in ethyl acetate (40 ml) and washed with saturated $NaHCO_3$ (2 x 15 ml), 1 M $KHSO_4$ (2 x 15 ml) and brine (15 ml). The organic layer was dried over magnesium sulphate and concentrated to dryness to give a yellow oil which was purified using Flash chromatography (20% ethyl acetate in hexane) to give *3-[2-(tert-butoxycarbonylamino)ethyl]-7-chloro-2-n-nonyl-4(3H)-quinazolinone* as a white solid (0.268 g, 29.8%).

A solution of 3-[2-(*tert*-butoxycarbonylamino)ethyl]-7-chloro-2-*n*-nonyl-4(3*H*)-quinazolinone (0.200 g, 4.4 mmol) in DCM (10 ml) and trifluoroacetic acid (10 ml) was stirred overnight at room temperature. The solution was concentrated to dryness under reduced pressure with

the aid of acetonitrile. The residue was dissolved in ethyl acetate (20 ml) and washed with saturated sodium bicarbonate (2 x 15 ml) and brine (10 ml). The organic was dried over magnesium sulphate and concentrated to dryness to give the title compound **37** as a cream white solid (0.091 g, 58.7%).

mp 69-72 ^oC

^1H NMR ($CDCl_3$) δ 0.93 (3H, t, J = 7 Hz, Me), 1.2-1.5 (12H, m, *$(CH_2)_6$*Me), 1.51 (2H, s, NH_2), 1.85 (2H, quintet, J = 7.8 Hz, *CH_2*$(CH_2)_6$Me), 2.87 (2H, t, J = 7.6 Hz, *CH_2*$(CH_2)_7$Me), 3.08 (2H, t, J = 7 Hz, *CH_2*NH_2), 4.19 (2H, t, J = 6.7 Hz, *CH_2*CH_2NH_2), 7.40 (1H, 8-H), 7.66 (1H, 6-H), 8.18 (1H, 5-H)
ES-MS *m/z* 350.2025 & 352.1950 $[M+H]^+$, $C_{19}H_{29}ClN_3O^+$ requires 350.1999 & 352.1970.

3-(3-Aminopropyl)-7-chloro-2-*n*-nonyl-4(3*H*)-quinazolinone (3-C3NH$_2$-7Cl-C9-QZN)

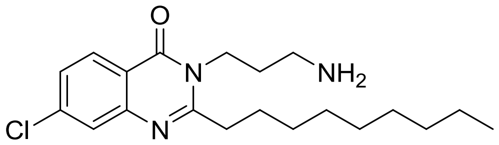

38

A solution of 7-chloro-2-nonyl-4*H*-benzo[*d*][1,3]oxazin-4-one (as described for **11**) (0.440 g, 1.43 mmol)and *N*-Boc-1,3-diaminopropane (1 g, 5.74 mmol) in dry THF (16 ml) was stirred at reflux overnight. The solvent was removed under reduced pressure and the residue was dissolved in ethyl acetate (30 ml) and washed with saturated $NaHCO_3$ (2 x 15 ml), 1 M $KHSO_4$ (2 x 15 ml) and brine (15 ml). The organic layer was dried over magnesium sulphate and concentrated to dryness to give a yellow oil which was purified using Flash chromatography (10% ethyl acetate in hexane) to give *3-[3-(tert-butoxycarbonylamino)propyl]-7-chloro-2-n-nonyl-4(3H)-quinazolinone* as a clear oily solid. (Yield = 0.133 g)
^1H NMR ($CDCl_3$) δ 0.92 (3H, t, *J* = 7.Hz, Me), 1.25-1.5 (21H, m, *$(CH_2)_6$*Me and C*$(CH_3)_3$*), 1.85 (2H, m, *CH_2*$(CH_2)_6$Me), 1.92 (2H, m, CH_2*CH_2*CH_2NH), 2.81 (2H, t, *CH_2*$(CH_2)_7$Me), 3.21 (2H, m, CH_2CH_2*CH_2*NH), 4.18 (2H, t, *CH_2*CH_2CH_2NH), 5.15 (1H, b, NH) 7.40 (1H, 6-H), 7.66 (1H, 5-H), 8.18 (1H, 8-H),
A solution of precursor 3-[3-(*tert*-butoxycarbonylamino)propyl]-7-chloro-2-*n*-nonyl-4(3*H*)-quinazolinone (0.130 g, 2.8 mmol) in DCM (7 ml) and trifluoroacetic acid (7 ml) was stirred overnight at room temperature. The solution was concentrated to dryness under reduced pressure with the aid of acetonitrile. The residue was dissolved in ethyl acetate (15 ml) and washed with saturated sodium bicarbonate (2 x 7.5 ml) and brine (7.5 ml). The organic layer was dried over magnesium sulphate and concentrated to dryness to give a cream solid which was purified by trituration with diethyl ether to give the title compound **38** as a white solid (0.029 g, 28.4%).

mp 82-95 ^oC

^{1}H NMR ($CDCl_3$) δ 0.92 (3H, t, J = 7 Hz, Me), 1.25-1.5 (12H, m, *$(CH_2)_6$*Me), 1.85 (2H, quintet, J = 7.6 Hz, *CH_2*$(CH_2)_6$Me), 2.08 (2H, quintet, J =6.6 Hz, *CH_2*CH_2NH_2) 2.83 (2H, t, J = 7.7 Hz, *CH_2*$(CH_2)_7$Me) 2.96 (2H, s, J = 7 Hz, *CH_2*NH_2), 3.15 (2H, b, NH_2), 3.15 (2H, b, NH_2) 4.26 (2H, t, J = 7 Hz, *CH_2*$(CH_2)_2NH_2$), 7.40 (1H, 8-H), 7.66 (1H, 6-H), 8.16 (1H, 5-H)
ES-MS *m/z* 364.2128 & 366.2149 $[M+H]^+$, $C_{20}H_{31}ClN_3O^+$ requires 364.2156 & 366.2126.

3-(3-Aminoethyl)-7-chloro-2-(3-phenylpropyl)-4(3*H*)-quinazolinone (3-C2NH$_2$-7Cl-PhC3-QZN)

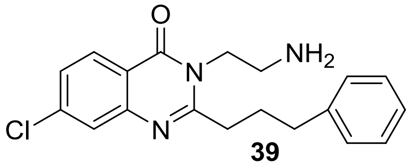

39

4-Phenylbutyryl chloride was prepared from 4-phenylbutyric acid by GP4.
N-(4-Phenylbutyryl)-4-chloroanthranilic acid was prepared as a pale brown solid in 74.1% yield by GP1 using 4-chloroanthranilic acid and 4-phenylbutyryl chloride and converted to 7-chloro-2-(3-phenylpropyl)-4*H*-benzo[*d*][1,3]oxazin-4-one as a pale brown solid in quantitative yield by GP2.
A solution of 7-chloro-2-(3-phenylpropyl)-4*H*-benzo[*d*][1,3]oxazin-4-one (0.599 g, 2 mmol) and *N*-Boc-ethylenediamine (1.28 g, 8 mmol) in dry THF (24 ml) was stirred at reflux overnight. The solvent was removed under reduced pressure and the residue was dissolved in ethyl acetate (40 ml) and washed with saturated $NaHCO_3$ (2 x 15 ml), 1 M $KHSO_4$ (2 x 15 ml) and brine (15 ml). The organic layer was dried over magnesium sulphate and concentrated to dryness to give a pale brown paste which was purified using Flash chromatography (10% to 30% ethyl acetate in hexane) to give *3-[2-(tert-butoxycarbonylamino)ethyl]-7-chloro-2-(3-phenylpropyl)-4(3H)-quinazolinone* as a white solid (0.220 g, 25.0%).
A solution of 3-[2-(*tert*-butoxycarbonylamino)ethyl]-7-chloro-2-(3-phenylpropyl)-4(3*H*)-quinazolinone (0.200 g, 4.4 mmol) in DCM (10 ml) and trifluoroacetic acid (10 ml) was stirred overnight at room temperature. The solution was concentrated to dryness under reduced pressure with the aid of acetonitrile. The residue was dissolved in ethyl acetate (20 ml) and washed with saturated sodium bicarbonate (2 x 15 ml) and brine (10 ml). The organic was dried over magnesium sulphate and concentrated to dryness to give an off white solid which was purified by trituration with diethyl ether to give the title quinazolinone **39** as a white solid (0.081 g, 56.8%).

mp 103-105 ^oC

^{1}H NMR ($CDCl_3$) δ 1.40 (2H, b, NH_2), 2.21 (2H, m, CH_2*CH_2*CH_2), 2.85 (4H, m, *CH_2*CH_2*CH_2*), 2.95 (2H, m, CH_2*CH_2*NH_2), 4.04 (2H, t, J = 6.9 Hz, *CH_2*CH_2NH_2), 7.20-7.36 (5H, m, Ph), 7.40 (1H, 6-H), 7.66 (1H, 8-H), 8.17 (1H, 5-H)
ES-MS *m/z* 342.1339 & 344.1371 $[M+H]^+$, $C_{19}H_{21}ClN_3O^+$ requires 342.1373 & 344.1344.

3-Amino-2-(3-phenylpropyl)-4(3*H*)-quinazolinone (3-NH$_2$-PhC3-QZN)

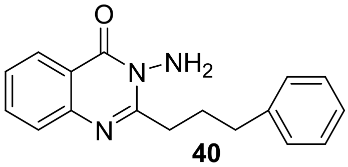

40

The title derivative **40** was prepared as a white solid in quantitative yield by GP3 using 2-(3-phenylpropyl) -4*H*-benzo[*d*][1,3]oxazin-4-one (as described above for **39**).

mp 113-116 ^{o}C

^{1}H NMR (CDCl$_3$) δ 2.24 (2H, quintet, J = 7.8 Hz,CH$_2$*CH$_2$*CH$_2$Ph), 2.84 (2H, t, J = 7.6 Hz, (CH$_2$)$_2$*CH$_2$*Ph), 3.15 (2H, t, *CH$_2$*(CH$_2$)$_2$Ph), 4.84 (2H, s, NH$_2$), 7.15(5H, m, Ph), 7.50 (1H, 8-H), 7.78 (2H, 6-H and 7-H), 8.25 (1H, 5-H)

ES-MS *m/z* 280.1422 [M+H]$^+$, $C_{17}H_{18}N_3O^+$ requires 280.1450.

3-Amino-7-chloro-2-(3-phenylpropyl)- 4(3*H*)-quinazolinone (3-NH$_2$-7Cl-PhC3-QZN)

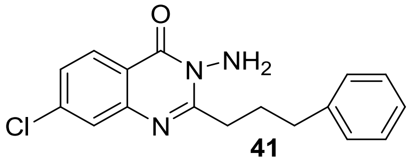

41

4-chloro-*N*-(4-phenylbutyryl)anthranilic acid was prepared as a pale brown solid in 74.1% yield by GP1using 4-chloroanthranilic acid and 4-phenylbutyryl chloride (as described for **39**) and converted to 7-chloro-2-(3-phenylpropyl) -4*H*-benzo[*d*][1,3]oxazin-4-one as a pale brown solid in quantitative yield by GP2.

The title quinazolinone was prepared by GP3 7-chloro-2-(3-phenylpropyl)-4*H*-benzo[*d*][1,3]oxazin-4-one. It was purified by Flash chromatography (15% ethyl acetate in hexane) to give **41** as a white solid (38.6%).

mp 115-117 ^{o}C

^{1}H NMR (CDCl$_3$) δ 2.21 (2H, m, CH$_2$*CH$_2$*CH$_2$), 2.82 (2H, t, J = 7.5 Hz, (CH$_2$)$_2$*CH$_2$*), 3.07 (2H, t, J = 7.4 Hz, *CH$_2$*(CH$_2$)$_2$), 4.79 (2H, s, NH$_2$), 7.20-7.35 (5H, m, Ph), 7.42 (1H, 6-H), 7.70 (1H, 8-H), 8.18 (1H, 5-H)

ES-MS *m/z* 314.1051 & 316.1006 [M+H]$^+$, $C_{17}H_{17}ClN_3O^+$ requires 314.1060 & 316.1031.

3-Amino-7-chloro -2-(2-(3,4,5-trimethoxyphenyl)ethyl)-4(3*H*)-quinazolinone (3-NH$_2$-7Cl-(TriOMeC$_6$H$_2$)C2-QZN)

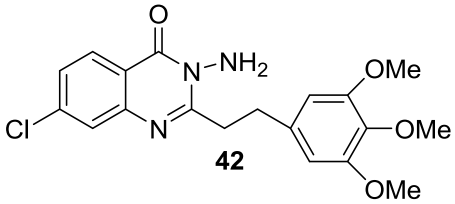

42

3-(3,4,5-Trimethoxyphenyl)propionyl chloride was prepared using 3-(3,4,5,-trimethoxyphenyl)propionyl acid by GP4.

4-chloro-2-(3-(3,4,5-trimethoxyphenyl)propionyl)anthranilic acid was prepared as a pale brown solid in 76.0% yield by GP1 using 4-chloroanthranilic acid and 3-(3,4,5,-trimethoxyphenyl)propionyl chloride and converted to *7-chloro -2-[2-(3,4,5-trimethoxyphenyl)ethyl]-4H-benzo[d][1,3]oxazin-4-one* as a pale brown solid in quantitative yield by GP2.

The title quinazolinone was prepared by GP3 from 7-chloro-2-[2-(3,4,5-trimethoxyphenyl)ethyl]-4H-benzo[d][1,3]oxazin-4-one. It was purified by trituration with diethyl ether and hexane to give **42** as a white solid (0.363 g, 93.2%).

mp 152-154 °C

^{1}H NMR ($CDCl_3$) δ 3.14 (2H, m, CH_2*CH_2*), 3.36 (2H, m, *CH_2*CH_2), 3.85,3.86,3.87 (3x3H, 3s, 3x 3OMe),4.79 (2H, s, NH_2), 6.53 (2H, s, Ar 2′-H, 6′-H), 7.45 (1H, 6-H), 7.73 (1H, 8-H), 8.20 (1H, 5-H)

ES-MS *m/z* 390.1166 & 392.1172 $[M+H]^+$, $C_{19}H_{21}ClN_3O_4^+$ requires 390.1221 & 392.1191.

3-Amino-7-chloro-2-(2-(indol-3-yl)ethyl)-4(3*H*)-quinazolinone (3-NH_2-7Cl-(3-Ind)C2-QZN)

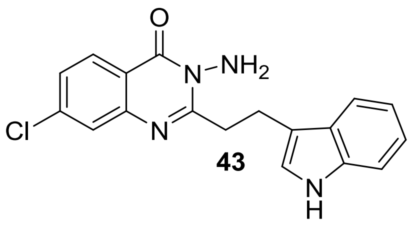

43

3-Indolepropionyl chloride was prepared by the following method

A solution of 3-indolepropionic acid (0.9 g, 4.8 mmol), thionyl chloride (0.66 g/0.4 ml, 5.5 mmol, 1.15 eqv), DMF (4 drops) in dry THF (50 ml) was stirred at 0 to 5 °C for 2h. The solution was then stored at 0 to 5 °C overnight after which the solvent was removed under reduced pressure and the resulting viscous brown oil was re-dissolved in dry THF (5 ml) and used directly (Yield = quantitative).

N-(3-Indolepropionyl)-4-chloroanthranilic acid was prepared as a brown solid in 98.0% yield by GP1 using 4-chloroanthranilic acid and 3-indolepropionyl chloride and converted to *7-chloro-2-(indol-3-yl)ethyl-4H-benzo[d][1,3]oxazin-4-one* as a brown solid in quantitative yield by GP2.

The title quinazolinone was prepared by GP3 from 7-chloro-2-(indol-3-yl)ethyl-4*H*-benzo[*d*][1,3]oxazin-4-one. It was purified Flash chromatography (2% methanol in DCM) and trituration with diethyl ether and ethyl acetate to give **43** as a yellow solid (16.2%).

mp 175-180 °C

^{1}H NMR (DMSO) δ 3.20 (2H, m, CH_2*CH_2*), 3.31 (2H, m, *CH_2*CH_2), 5.84 (2H, s, NH_2), 7.00 (1H, m, Ind 6-H), 7.08 (1H, m, Ind 5-H), 7.22 (1H, s, Ind 2-H), 7.35 (1H, d, Ind 7-H), 7.54 (1H, 6H), 7.62 (1H, d, Ind 4-H), 7.75 (1H, 8H), 8.13 (1H, 5H), 10.80 (1H, s, NH)

ES-MS *m/z* 339.1003 & 341.0953 $[M+H]^+$, $C_{18}H_{16}ClN_4O^+$ requires 339.1013 & 341.0983.

3-Amino-7-chloro-2-(4-biphenylmethyl)-4(3*H*)-quinazolinone (3-NH$_2$-7Cl-BiPh-C1-QZN)

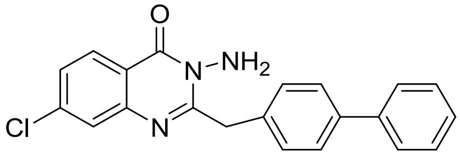

44

4-Biphenylacetyl chloride was prepared by GP4 using 4-biphenylacetic acid.

4-chloro-*N*-(4-biphenylacetyl)anthranilic acid was prepared as a pale brown solid in 69.7% yield by GP1 using 4-chloroanthranilic acid and 4-biphenylacetyl chloride and converted to *7-chloro-2-(4-biphenylmethyl)-*4*H-benzo[d][1,3]oxazin-4-one* as a pale brown solid in 95.2% yield by GP2.

The title quinazolinone was prepared by GP3 from 7-chloro-2-(4-biphenylmethyl)-4*H*-benzo[*d*][1,3]oxazin-4-one. It was purified by recrystallisation from ethyl acetate to give **44** an off white solid (32.7%).

mp 186-187 ^{o}C

^{1}H NMR (DMSO) δ 4.38 (2H, s, *CH$_2$*Ph), 5.77 (2H, s, NH$_2$), 7.30-7.71 (11H, m, biphHs & 6-H & 8-H), 8.14 (1H, 5-H)

ES-MS *m/z* 362.1057 & 364.1049 [M+H]$^+$, $C_{21}H_{17}ClN_3O$ requires 362.1060 & 364.1031.

3-Amino-7-chloro -2-(4-phenoxybenzyl)-4(3*H*)-quinazolinone (3-NH$_2$-7Cl-PhOBn-QZN)

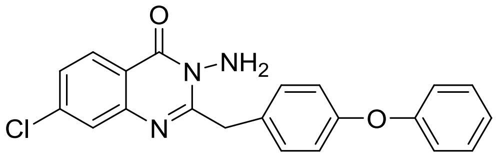

45

4-Phenoxyphenylacetyl chloride was prepared by GP4 using 4-phenoxyphenylacetic acid.

N-(4-Phenoxyphenylacetyl)-4-chloroanthranilic acid was prepared as a pale brown solid in quantitative yield by GP1 using 4-chloroanthranilic acid and 4-phenoxyphenylacetyl chloride and converted to *7-chloro -2-(4-phenoxybenzyl)-*4*H-benzo[d][1,3]oxazin-4-one* was prepared as a pale brown solid in 73.4% yield by GP2.

The title quinazolinone was prepared by GP3 from 7-chloro-2-*(*4-phenoxybenzyl)-4*H*-benzo[*d*][1,3]oxazin-4-one. It was purified by recrystallisation from ethyl acetate to give **45** as an off white solid (40.5%).

mp 144-145 ^{o}C

^{1}H NMR (CDCl$_3$) δ 4.36 (2H, s, *CH$_2$*Ph), 4.81 (2H, s, NH$_2$), 6.97-7.46 (10H, m, $C_6H_5OC_6H_4$ & 6-H), 7.76 (1H, 8-H), 8.20 (1H, 5-H)

ES-MS *m/z* 378.0970 & 380.1033[M+H]$^+$, $C_{21}H_{17}ClN_3O_2^+$ requires 378.1009 & 380.0980.

3-Amino-2-(3-cyclohexylpropyl)-4(3*H*)-quinazolinone (3-NH_2-CyC3-QZN)

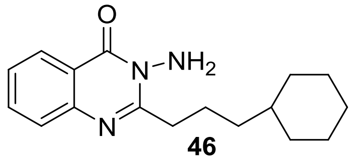

46

A mixture of 4-cyclohexanebutyric acid (4.46 g, 0.0261 mol), thionyl chloride (3.26 g, 2 ml, 0.0274 mol, 1.05 eqv), DMF (4 drops) in toluene (10 ml) was stirred at room temperature for 15 min. The solution was then stirred at 60 to 65 ^{o}C for 1h and the solvent was removed under reduced pressure to give 4-cyclohexanebutyryl chloride as a clear oil in quantitative yield.

N-(4-Cyclohexanebutyryl)anthranilic acid was prepared in 86% yield as a pale brown solid by GP1 using anthranilic acid and 4-cyclohexanebutyryl chloride and converted to

2-(3-cyclohexanepropyl)-4H-benzo[d][1,3]oxazin-4-one was prepared as a pale brown oil in quantitative yield by GP2.

The title quinazolinone **46** was prepared as a white solid in quantitative yield by GP3 using 2-*(*3-cyclohexanepropyl)-4*H*-benzo[*d*][1,3]oxazin-4-one.

mp 110-113 ^{o}C

^{1}H NMR ($CDCl_3$) δ 0.8-1.8 (13H, m, $(CH_2)_2CH_2$ and cyHs), 1.88 (2H, quintet, J = 8 Hz, $CH_2CH_2CH_2$), 3.10 (2H, t, $CH_2(CH_2)_2$, 4.92 (2H, s, NH_2), 7.49 (1H, 8-H), 7.78 (2H, 6-H and 7-H), 8.26 (1H, 5-H)

ES-MS *m/z* 286.1903 $[M+H]^+$, $C_{17}H_{24}N_3O^+$ requires 286.1919.

3-Amino-8-aza-2-*n*-nonyl-4(3*H*)-quinazolinone (3-NH_2-8Aza-C9-QZN)

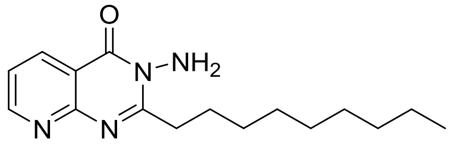

47

The precursor *2-(decanamido)pyridine-3-carboxylic acid* was prepared as follows:

Decanoyl chloride (2.07 g, 10.88 mmol) was added dropwise over 5 min at room temperature to a solution of 2-aminonicotinic acid (1 g, 7.25 mmol) and DIPEA (1.88 g, 14.5 mmol) in DMF (5 ml). This was stirred at room temperature for 3 days and poured into ice. The mixture was extracted with dichloromethane (2 x 20 ml) which was then washed with saturated sodium bicarbonate and brine. The organic layer was dried with magnesium sulphate and the solvent was removed under reduced pressure to give *2-(decanamido)pyridine-3-carboxylic acid* as a yellow solid (1.53 g, 72.3%) and converted to *2-nonyl-4H-pyrido[2,3-d][1,3]oxazin-4-one* as a brown residue in quantitative yield by GP2.

The title quinazolinone was prepared by GP3 using 2-*n*-nonyl-4*H*-pyrido[2,3-d][1,3]oxazin-4-one. The crude product was stirred in dichloromethane and filtered. The filtrate was concentrated, the residue purified by Flash chromatography (100% ethyl acetate) and trituration of the product with diethyl ether to give **47** as a cream solid (0.013 g)

mp 81-83 ^{o}C

^{1}H NMR ($CDCl_3$) δ 0.90 (3H, t, J = 7 Hz, Me), δ 1.25-1.5 (12H, m, $(CH_2)_6$Me), 1.93 (2H, m, $CH_2(CH_2)_6$Me), 3.11 (2H, t, J = 7.9 Hz, $CH_2(CH_2)_7$Me), 4.88 (2H, s, NH_2), 7.42 (1H, 6-H), 8.60 (1H, 5-H), 9.00 (1H, 7-H)

ES-MS *m/z* 289.2017 $[M+H]^+$, $C_{16}H_{25}N_4O^+$ requires 289.2028.

References for Text S1

1. Terwilliger TC, Berendzen J (1999) Automated MAD and MIR structure solution. Acta Crystallogr D Biol Crystallogr 55: 849-861.
2. Emsley P, Cowtan K (2004) *Coot*: model-building tools for molecular graphics. Acta Crystallogr D Biol Crystallogr 60: 2126-2132.
3. Pesci EC, Milbank JBJ, Pearson JP, McKnight S, Kende AS, et al. (1999) Quinolone signaling in the cell-to-cell communication system of *Pseudomonas aeruginosa*. Proc Natl Acad Sci U S A 96: 11229-11234.
4. Pritchard DI, Bycroft BW, Chhabra SR, Hooi D (2002) Substituted 4-quinolones. *PCT Int. Appl.* WO/2002/047686.
5. Hradil P, Hlavac J, Lemr K (1999) Preparation of 1,2-disubstituted-3-hydroxy-4(1*H*)-quinolinones and the influence of substitution on the course of cyclization. J Heterocycl Chem 36: 141-144.
6. Purcell IC (2007) Bacterial autoinducer derived 4-quinolones as novel immune modulators: University of Nottingham.
7. Hodgkinson JT, Galloway WRJD, Saraf S, Baxendale IR, Ley SV, et al. (2011) Microwave and flow syntheses of *Pseudomonas* quinolone signal (PQS) and analogues. Org Biomol Chem 9: 57-61.
8. De Kimpe N, De Cock W, Schamp N (1987) A convenient synthesis of 1-chloro-2-alkanones. Synthesis: 188-190.
9. Somanathan R, Smith KM (1981) Synthesis of some 2-alkyl-4-quinolone and 2-alkyl-4-methoxyquinoline alkaloids. J Heterocycl Chem 18: 1077-1079.
10. Houghton RP, Lapham DJ (1982) A modified preparation of b-keto-esters. Synthesis: 451-452.
11. Mahmoud MR, El-Bordany EAA, Hassan NF, Abu El-Azm FSM (2007) New 2,3-disubstituted quinazolin-4(3*H*)-ones from 2-undecyl-3,1-benzoxazin-4-one. J Chem Res: 541-544.
12. Watson AJA, Maxwell AC, Williams JMJ (2012) Ruthenium-catalysed oxidative synthesis of heterocycles from alcohols. Org Biomol Chem 10: 240-243.

Supplementary Data. ^{1}H NMR Spectra of selected compounds

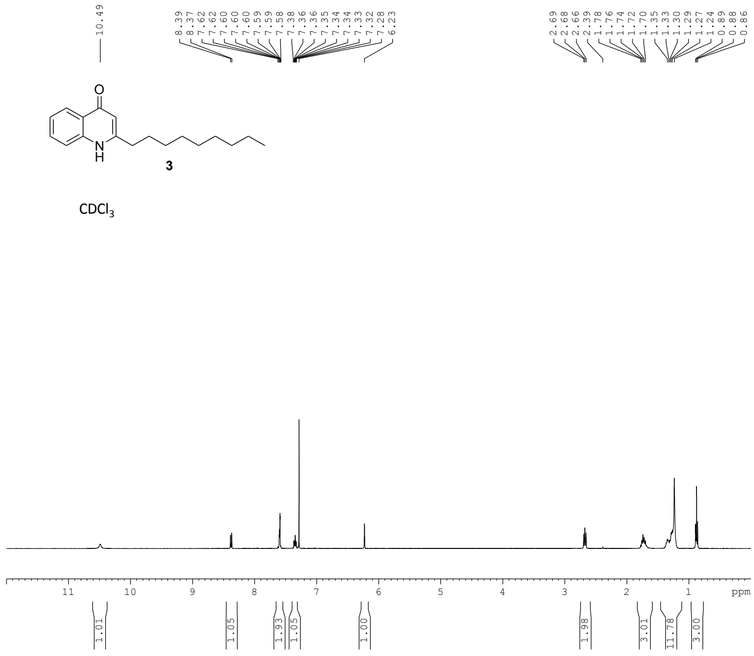

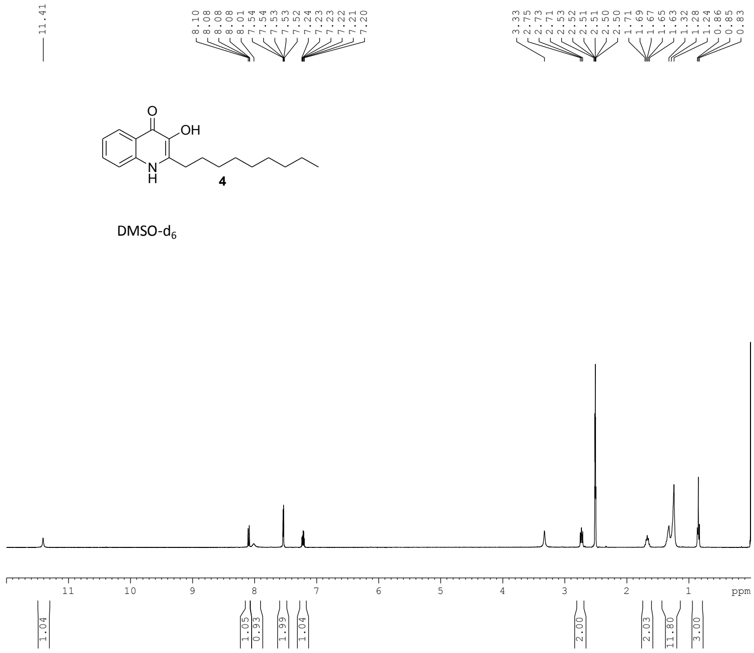

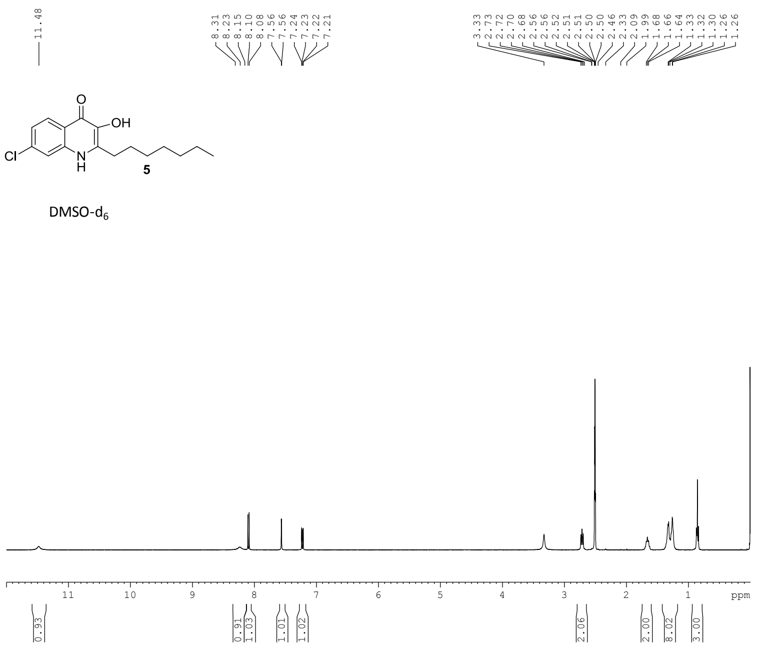

5
DMSO-d$_6$
ppm

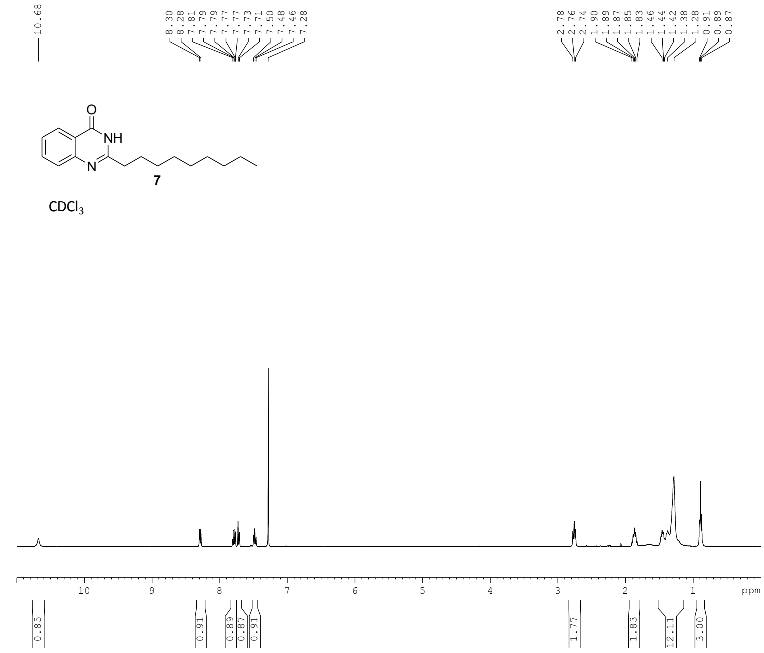

7
CDCl$_3$
ppm

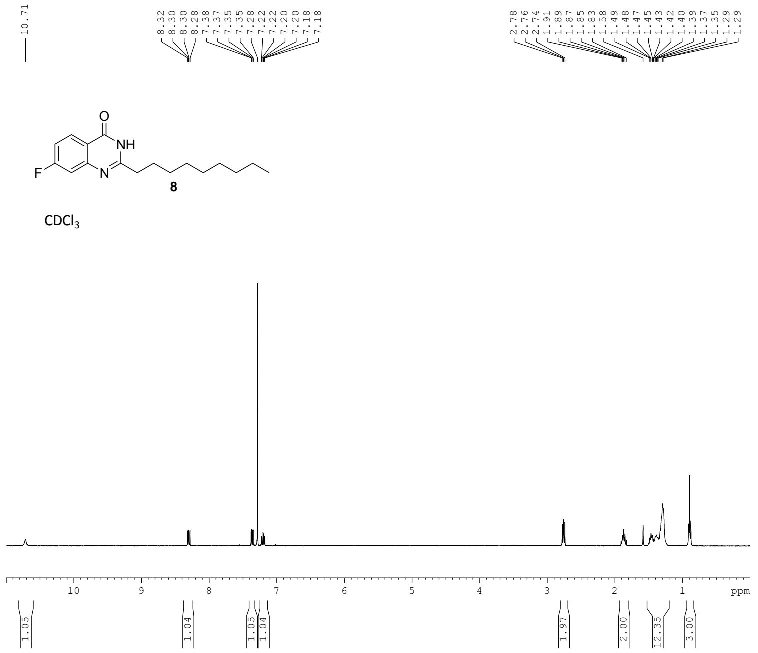
8
CDCl3
ppm

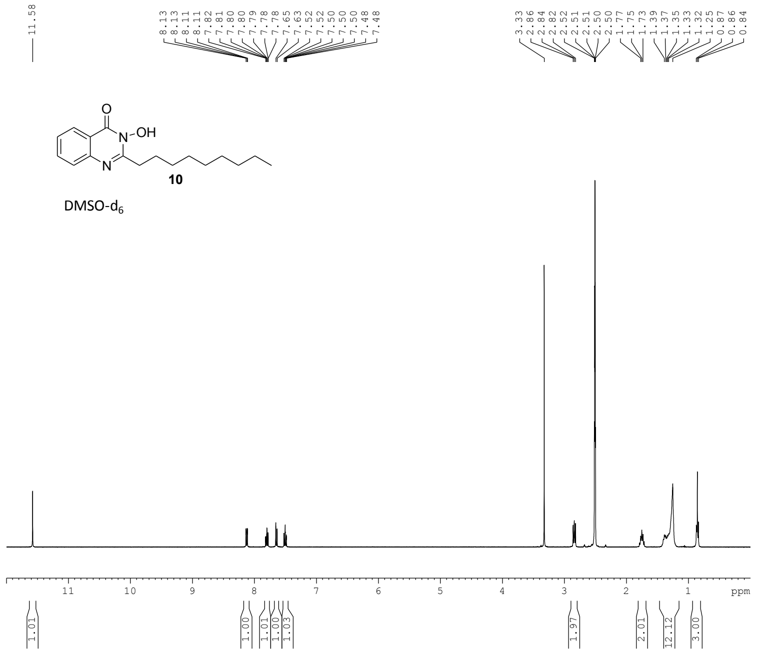
N-OH
10
DMSO-d6
ppm

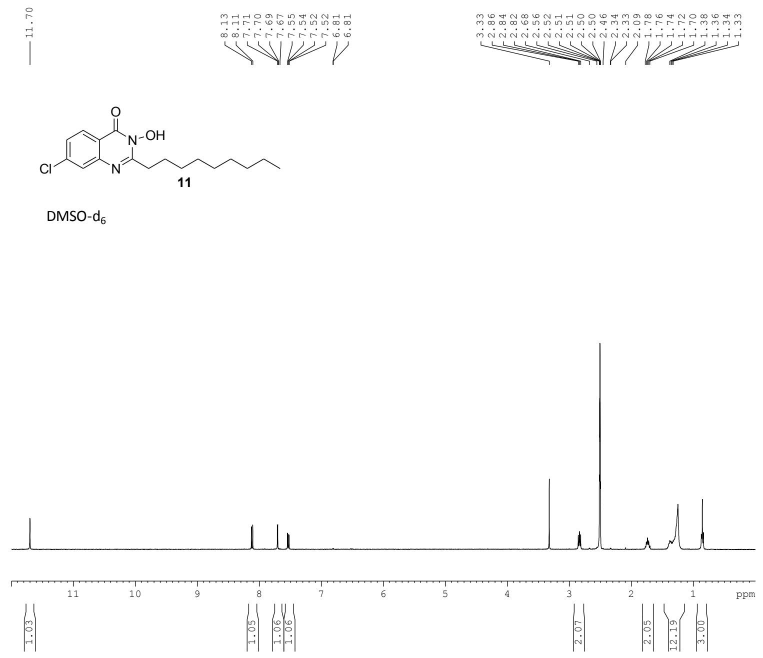
11.70
8.13 8.11 7.71 7.70 7.69 7.67 7.55 7.54 7.52 7.52 6.81 6.81
3.33 2.86 2.84 2.82 2.68 2.56 2.52 2.51 2.51 2.50 2.50 2.46 2.34 2.33 2.09 1.78 1.76 1.74 1.72 1.70 1.38 1.36 1.34 1.33
O
N
OH
Cl
N
11
DMSO-d6
11 10 9 8 7 6 5 4 3 2 1 ppm
1.03 1.05 1.06 1.06 2.07 2.05 12.19 3.00

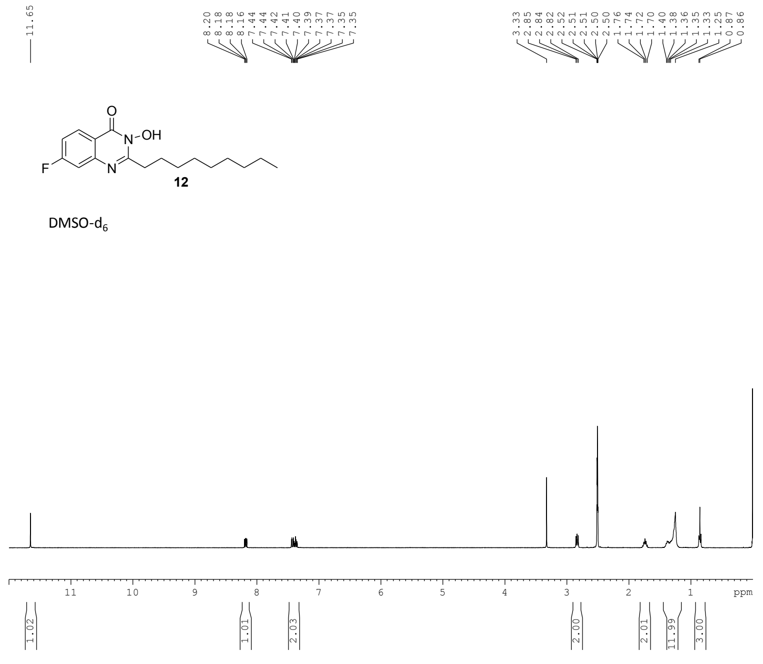
11.65
8.20 8.18 8.18 8.16 7.44 7.44 7.42 7.41 7.40 7.39 7.37 7.35 7.35
3.33 2.85 2.84 2.82 2.52 2.51 2.51 2.50 2.50 1.76 1.74 1.72 1.70 1.40 1.38 1.36 1.35 1.33 1.25 0.87 0.86
O
N
OH
F
N
12
DMSO-d6
11 10 9 8 7 6 5 4 3 2 1 ppm
1.02 1.01 2.03 2.00 2.01 11.99 3.00

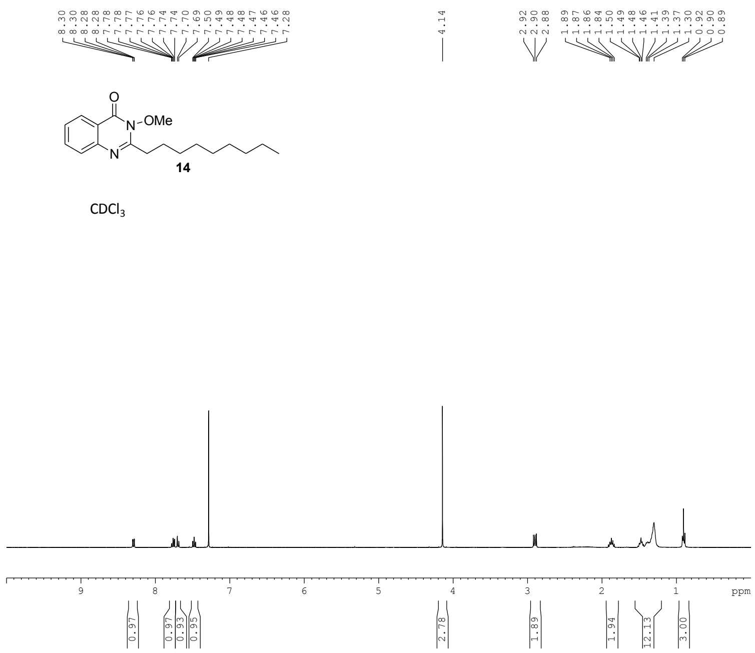
OMe
14
CDCl3
ppm

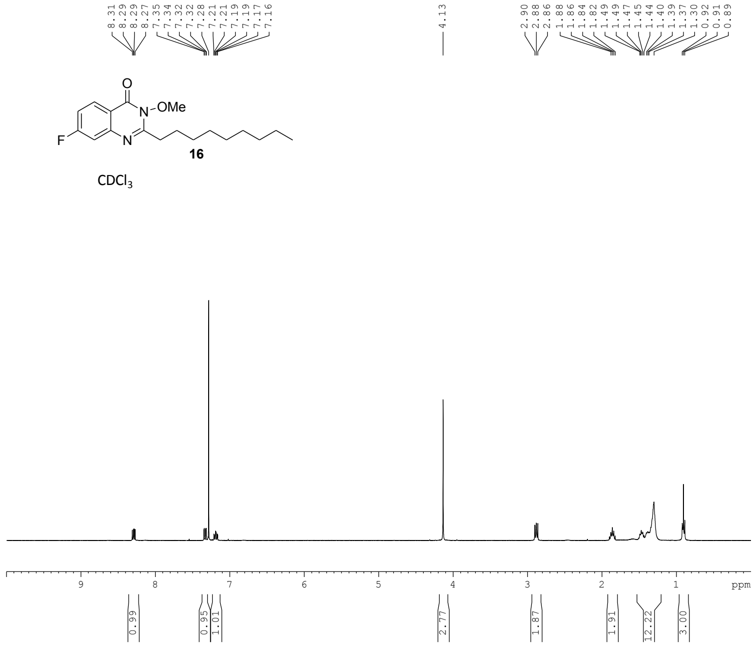
OMe
F
16
CDCl3
ppm

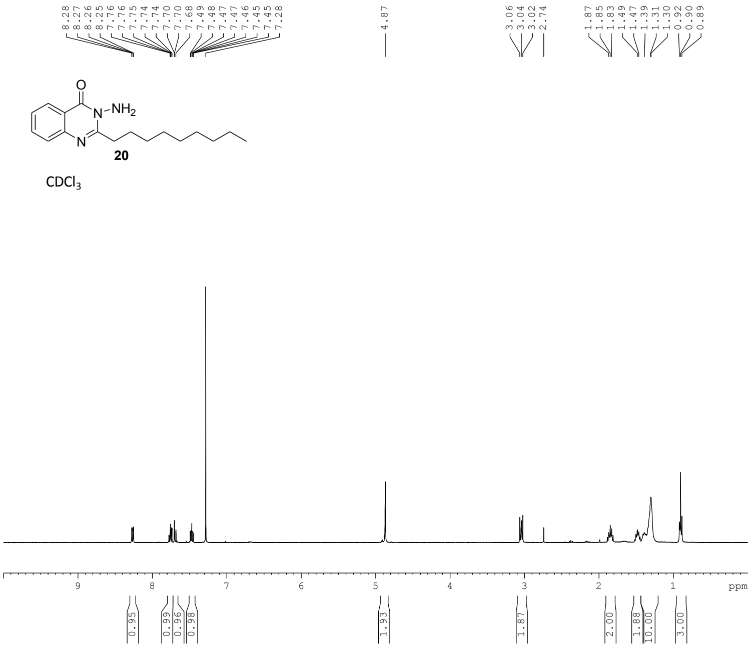
8.28
8.27
8.26
8.25
7.76
7.75
7.74
7.74
7.70
7.70
7.68
7.49
7.48
7.47
7.47
7.46
7.45
7.28
4.87
3.06
3.04
3.02
2.74
1.87
1.85
1.83
1.49
1.47
1.39
1.31
1.30
0.92
0.90
0.89
O
N
NH2
N
20
CDCl3
9
8
7
6
5
4
3
2
1
ppm
0.95
0.99
0.96
0.98
1.93
1.87
2.00
1.88
10.00
3.00

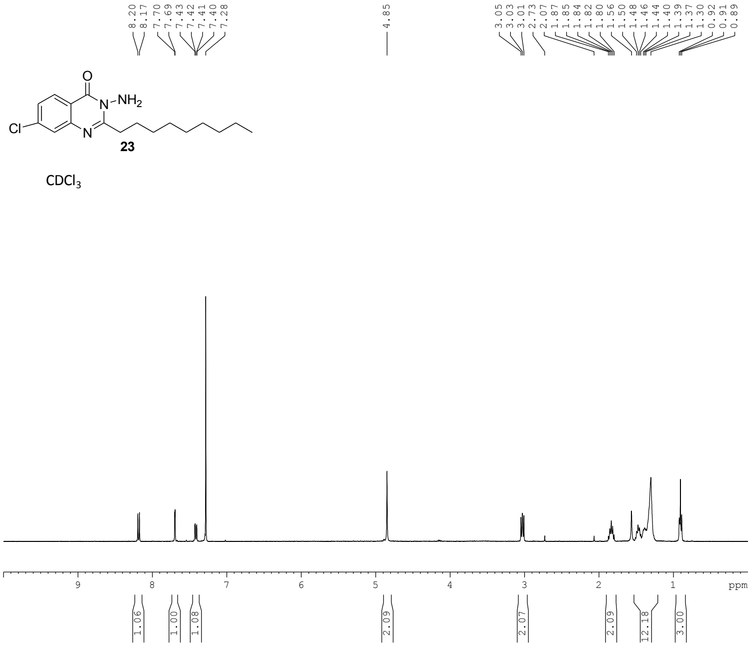
8.20
8.17
7.70
7.69
7.43
7.42
7.41
7.40
7.28
4.85
3.05
3.03
3.01
2.73
2.07
1.87
1.85
1.84
1.82
1.80
1.56
1.50
1.48
1.46
1.44
1.40
1.39
1.37
1.30
1.32
0.92
0.91
0.89
O
N
NH2
Cl
N
23
CDCl3
9
8
7
6
5
4
3
2
1
ppm
1.06
1.00
1.08
2.09
2.07
2.09
12.18
3.00

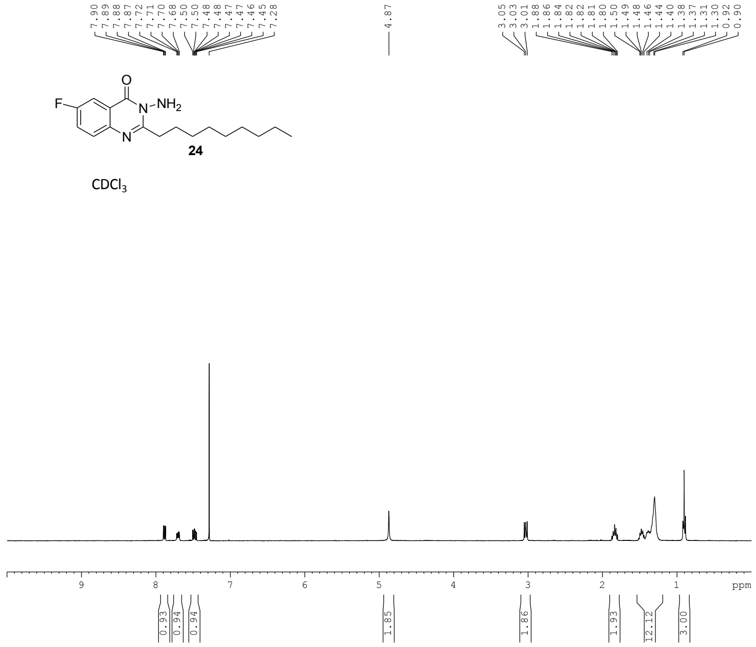
7.90
7.89
7.88
7.87
7.72
7.71
7.70
7.68
7.50
7.50
7.48
7.48
7.47
7.47
7.46
7.45
7.28
4.87
3.05
3.03
3.01
1.88
1.86
1.84
1.82
1.82
1.81
1.80
1.50
1.49
1.48
1.46
1.44
1.40
1.38
1.37
1.31
1.30
0.92
0.90
F
O
N
NH2
N
24
CDCl3
9
8
7
6
5
4
3
2
1
ppm
0.93
0.94
0.94
1.85
1.86
1.93
12.12
3.00

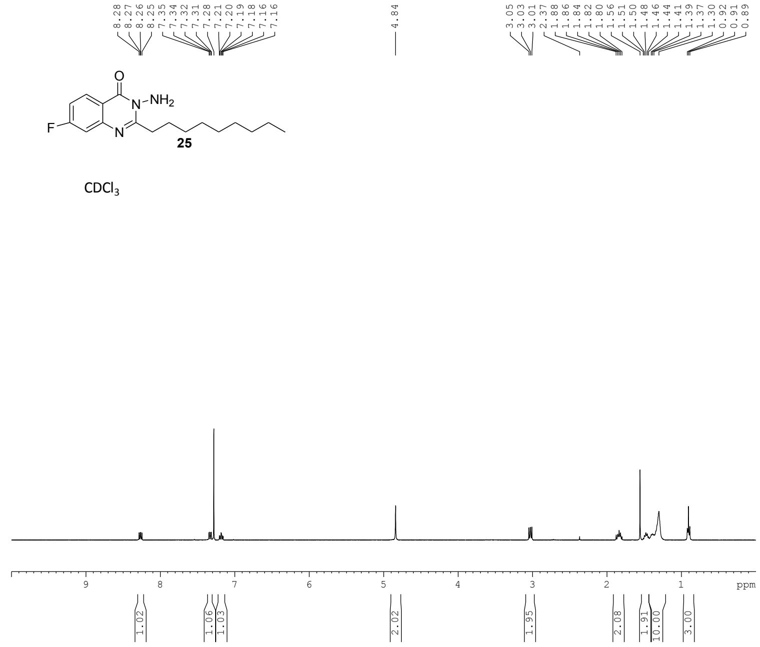
8.28
8.27
8.26
8.25
7.35
7.34
7.32
7.31
7.28
7.21
7.20
7.19
7.18
7.16
7.16
4.84
3.05
3.03
3.01
2.37
1.88
1.86
1.84
1.82
1.80
1.56
1.51
1.50
1.48
1.46
1.44
1.41
1.39
1.37
1.30
0.92
0.91
0.89
F
O
N
NH2
N
25
CDCl3
9
8
7
6
5
4
3
2
1
ppm
1.02
1.06
1.03
2.02
1.95
2.08
1.91
10.00
3.00

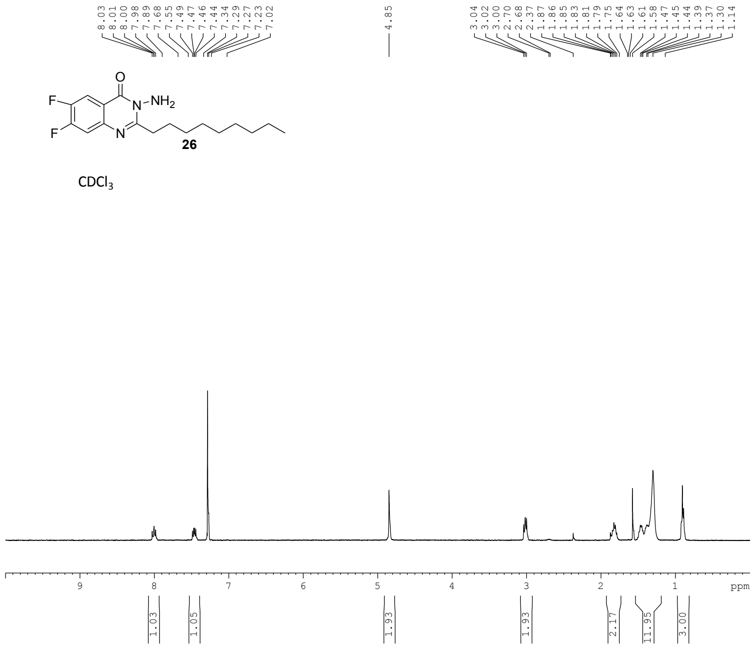
8.03 8.01 8.00 7.98 7.89 7.68 7.55 7.49 7.47 7.46 7.44 7.34 7.29 7.27 7.23 7.02
4.85
3.04 3.02 3.00 2.70 2.68 2.37 1.87 1.86 1.85 1.83 1.81 1.79 1.75 1.64 1.63 1.61 1.58 1.47 1.45 1.44 1.39 1.37 1.30 1.14
O
F
F
N
N
NH2
26
CDCl3
9 8 7 6 5 4 3 2 1 ppm
1.03 1.05 1.93 1.93 2.17 11.95 3.00

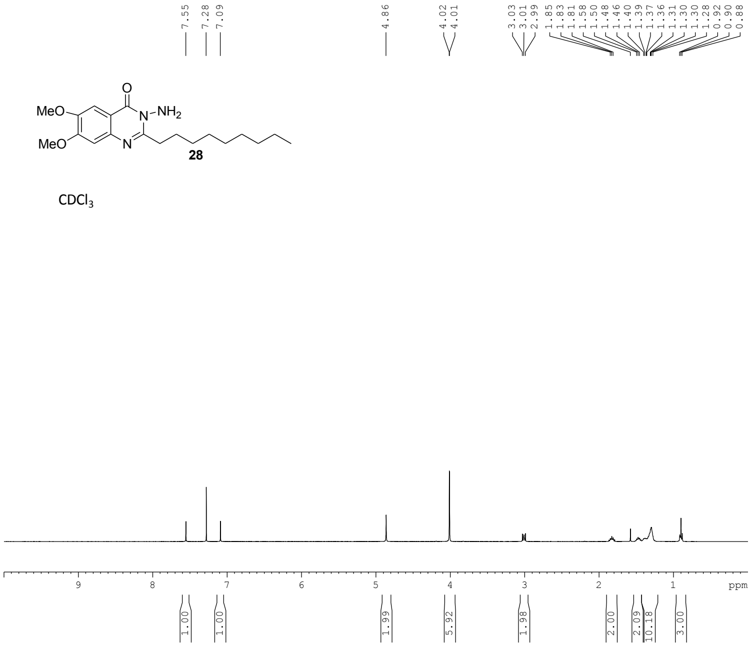
7.55 7.28 7.09
4.86
4.02 4.01
3.03 3.01 2.99 1.85 1.83 1.81 1.58 1.50 1.48 1.46 1.40 1.39 1.37 1.36 1.31 1.30 1.30 1.28 0.92 0.90 0.88
O
MeO
MeO
N
N
NH2
28
CDCl3
9 8 7 6 5 4 3 2 1 ppm
1.00 1.00 1.99 5.92 1.98 2.00 2.09 10.18 3.00

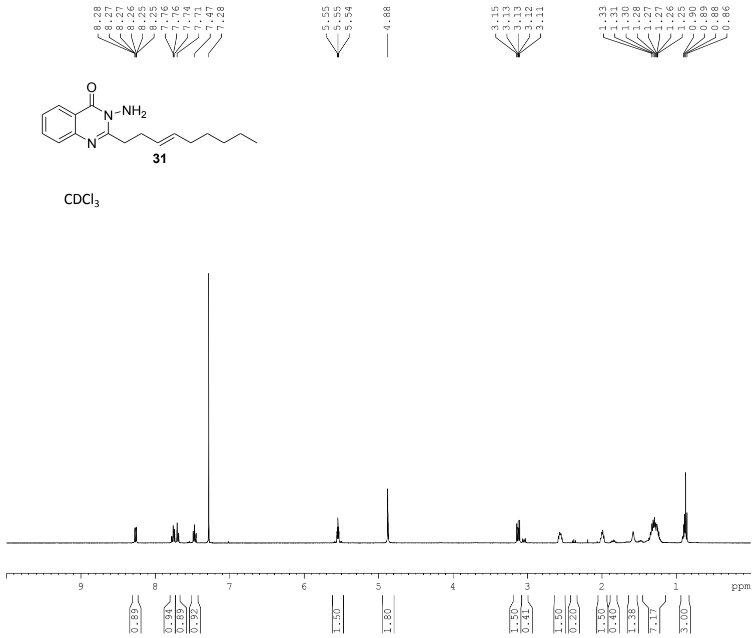
8.28
8.27
8.26
8.25
8.25
7.76
7.76
7.74
7.71
7.47
7.28
5.55
5.55
5.54
4.88
3.15
3.13
3.13
3.12
3.11
1.33
1.31
1.30
1.28
1.27
1.27
1.26
1.25
0.90
0.89
0.88
0.86
O
N
NH2
N
31
CDCl3
9
8
7
6
5
4
3
2
1
ppm
0.89
0.94
0.89
0.92
1.50
1.80
1.50
0.41
1.50
0.20
1.50
0.40
1.38
7.17
3.00

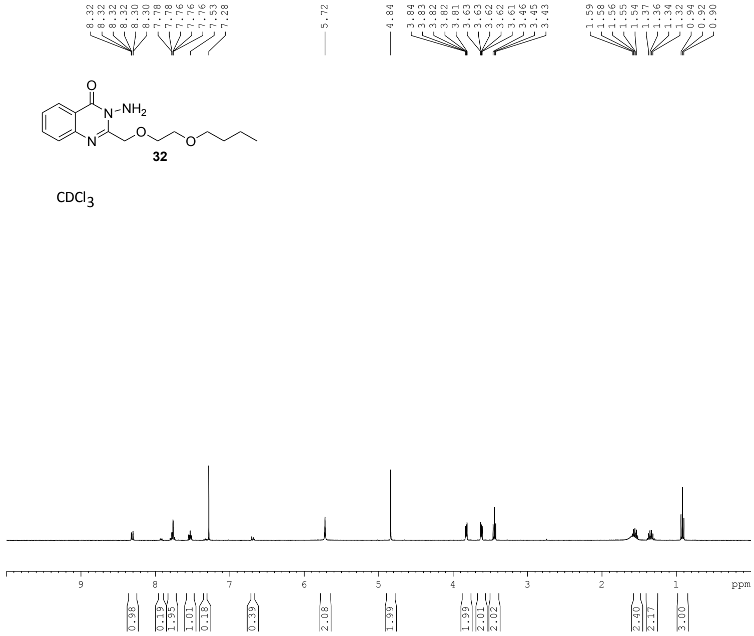
8.32
8.32
8.32
8.30
8.30
7.78
7.76
7.76
7.76
7.53
7.28
5.72
4.84
3.84
3.83
3.82
3.82
3.81
3.63
3.63
3.62
3.61
3.46
3.45
3.43
1.59
1.58
1.56
1.55
1.54
1.37
1.36
1.34
1.32
0.94
0.92
0.90
O
N
NH2
N
O
O
32
CDCl3
9
8
7
6
5
4
3
2
1
ppm
0.98
0.19
1.95
1.01
0.18
0.39
2.08
1.99
1.99
2.01
2.02
2.40
2.17
3.00

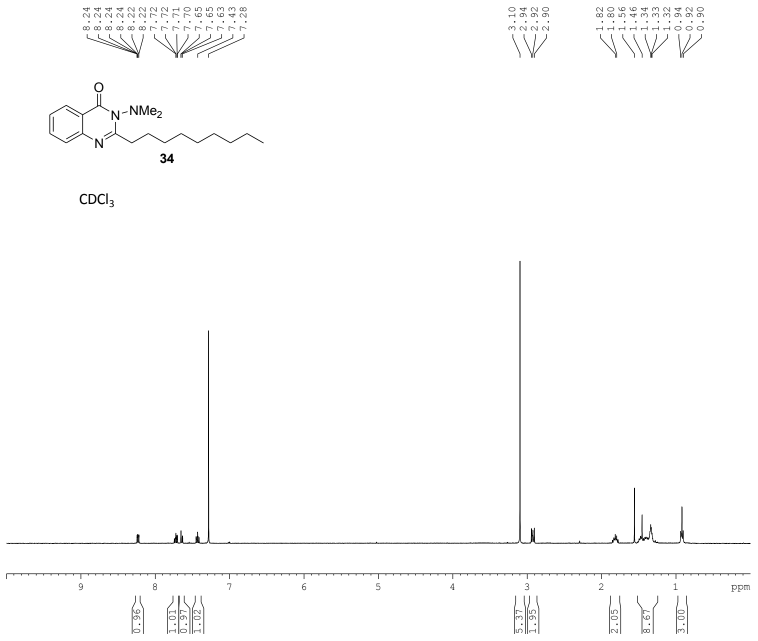
8.24 8.24 8.24 8.24 8.22 8.22 7.72 7.72 7.71 7.70 7.65 7.65 7.63 7.43 7.28
3.10 2.94 2.92 2.90
1.82 1.80 1.56 1.46 1.34 1.33 1.32 0.94 0.92 0.90
O
N
NMe2
N
34
CDCl3
9 8 7 6 5 4 3 2 1 ppm
0.96 1.01 0.97 1.02 5.37 1.95 2.05 8.67 3.00

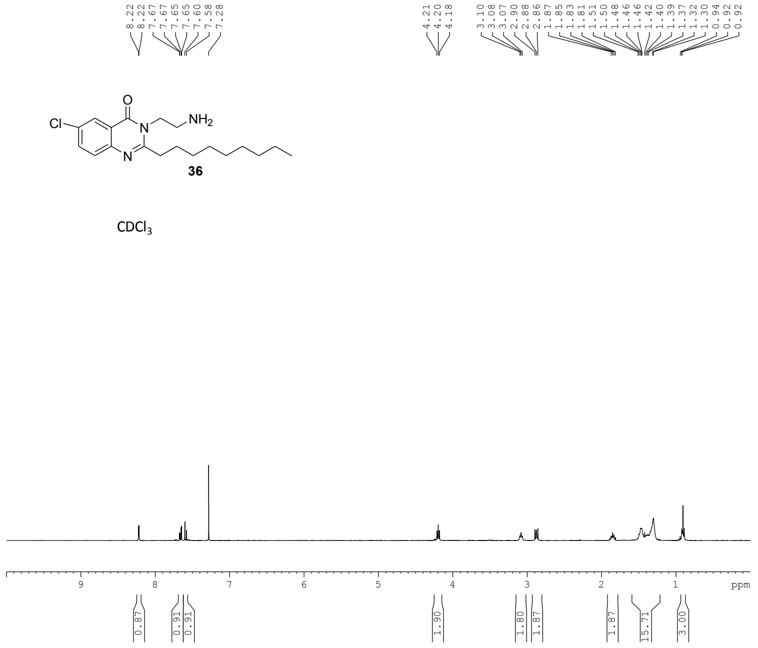
8.22 8.22 7.67 7.67 7.65 7.65 7.60 7.58 7.28
4.21 4.20 4.18
3.10 3.08 3.07 2.90 2.88 2.86 1.87 1.85 1.83 1.81 1.51 1.50 1.48 1.46 1.42 1.40 1.39 1.37 1.32 1.30 0.94 0.92 0.92
Cl
O
N
NH2
N
36
CDCl3
9 8 7 6 5 4 3 2 1 ppm
0.87 0.91 0.91 1.90 1.80 1.87 1.87 15.71 3.00

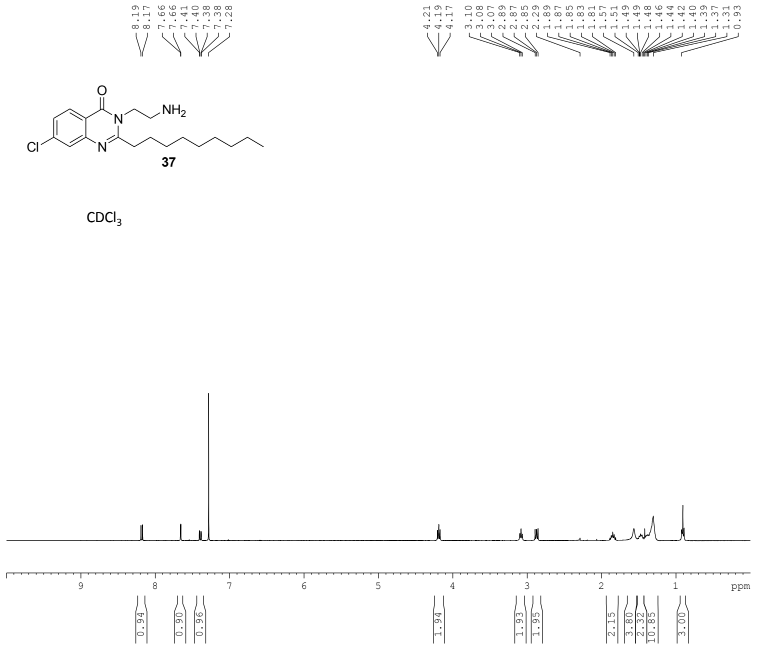
8.19 8.17 7.66 7.41 7.40 7.38 7.28
4.21 4.19 4.17 3.10 3.08 3.07 2.89 2.87 2.85 2.29 1.89 1.87 1.85 1.83 1.81 1.57 1.51 1.49 1.48 1.46 1.44 1.42 1.40 1.39 1.37 1.31 0.93
O
N
NH2
Cl
37
CDCl3
9 8 7 6 5 4 3 2 1 ppm
0.94 0.90 0.96 1.94 1.93 1.95 2.15 3.80 2.32 10.85 3.00

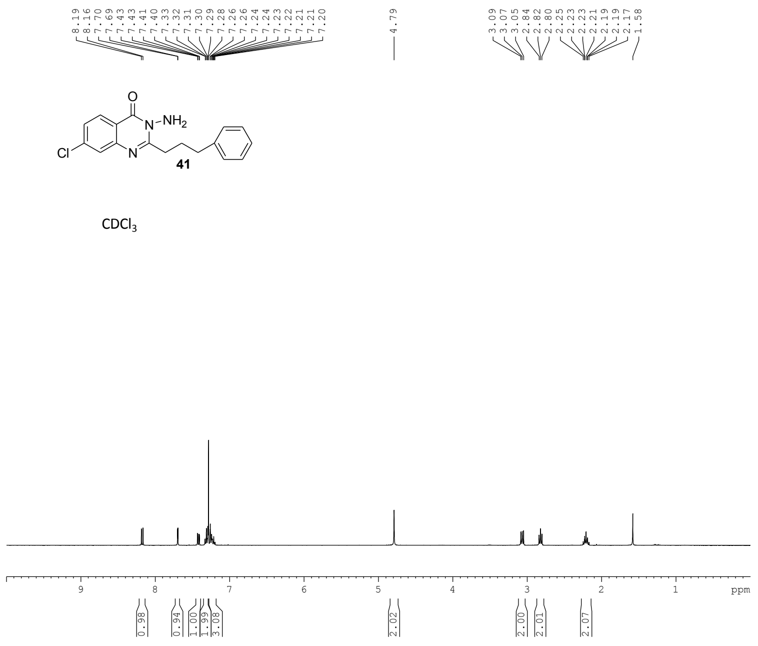
8.19 8.16 7.70 7.69 7.43 7.41 7.40 7.33 7.32 7.31 7.30 7.29 7.28 7.26 7.24 7.24 7.23 7.22 7.21 7.21 7.20
4.79
3.09 3.07 3.05 2.84 2.82 2.80 2.25 2.23 2.21 2.19 2.17 1.58
O
N
NH2
Cl
41
CDCl3
9 8 7 6 5 4 3 2 1 ppm
0.98 0.94 1.00 1.99 3.08 2.02 2.00 2.01 2.07

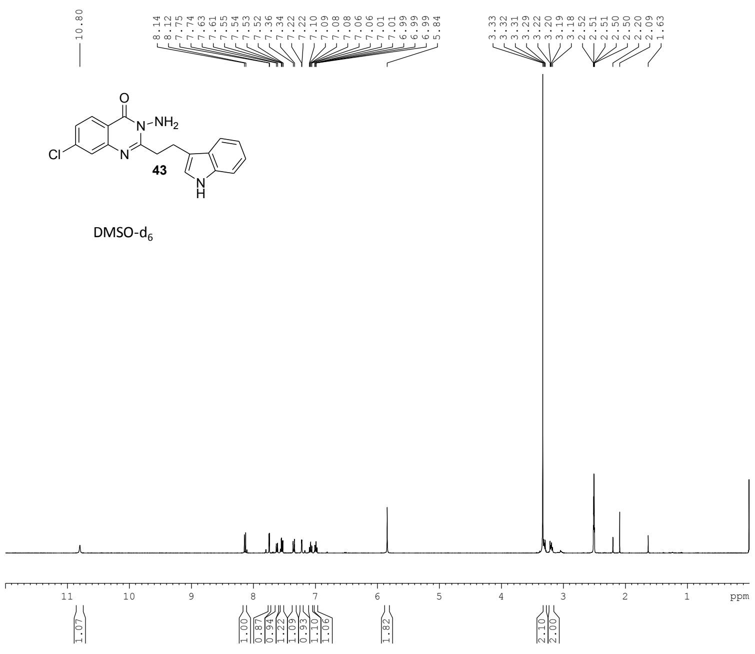
43
DMSO-d6
ppm

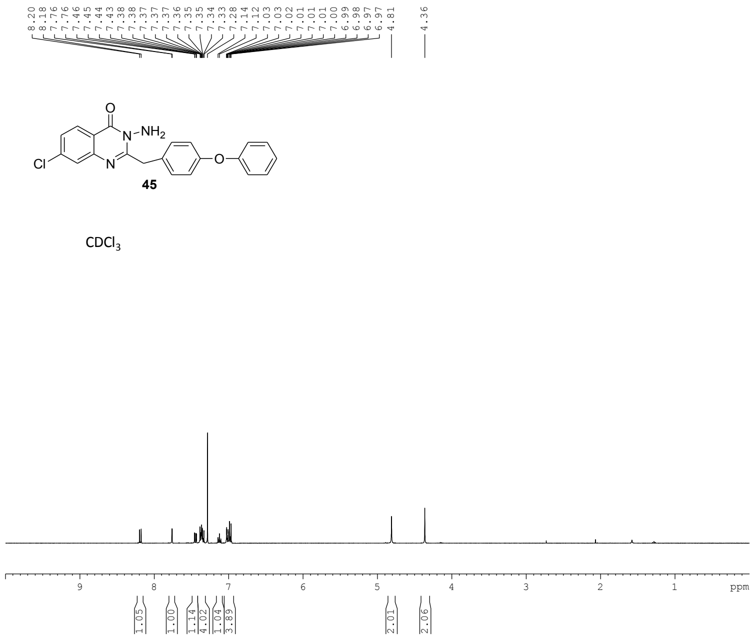
45
CDCl3
ppm

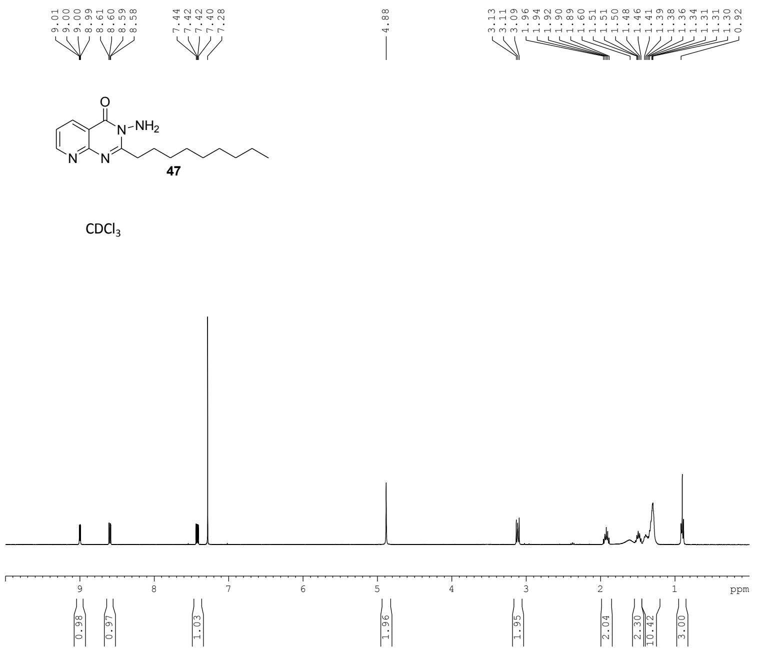
9.01
9.00
9.00
8.99
8.61
8.60
8.59
8.58
7.44
7.42
7.42
7.40
7.28
4.88
3.13
3.11
3.09
1.96
1.94
1.92
1.90
1.89
1.60
1.51
1.51
1.50
1.48
1.46
1.41
1.39
1.38
1.36
1.34
1.31
1.30
0.92
O
N
NH2
N
N
47
CDCl3
9
8
7
6
5
4
3
2
1
ppm
0.98
0.97
1.03
1.96
1.95
2.04
2.30
10.42
3.00
